# Machine learning–optimized discharge timing in typhoid care: Implications for clinical outcomes, cost efficiency, and health system performance

**DOI:** 10.1371/journal.pone.0354148

**Published:** 2026-07-23

**Authors:** Shekoofeh Sadat Momahhed, Atefehsadat Haghighathoseini

**Affiliations:** 1 National Centre for Health Insurance Research, Tehran, Iran; 2 Department of Health Administration and Policy, College of Public Health, George Mason University, Fairfax, Virginia, United States of America; Covenant University, NIGERIA

## Abstract

**Objective:**

To develop and validate a machine learning model identifying typhoid fever patients at risk of unnecessarily prolonged hospitalization, and to quantify the clinical, financial, and systemic consequences of model-guided discharge optimization in a fragmented, multi-payer public insurance system.

**Setting:**

Iran Health Insurance Organization inpatient claims for typhoid fever, all provinces, 2024–2025; 80,223 raw claims aggregated into 13,105 hospitalization episodes.

**Design:**

Retrospective observational study with embedded computational modelling. A gradient-boosted classifier was developed on administrative claims and validated through stratified five-fold cross-validation. Predictions were translated into clinical, financial, and systemic impact levels.

**Results:**

The model achieved an area under the receiver operating characteristic curve of 0.862, outperforming logistic regression (0.831). Among 13,105 episodes, 4,658 patients were identified as potentially having reducible length of stay, projecting 6,459 potentially recoverable bed-days pending clinical review. Restricted to 5,784 inpatient admissions, optimized discharge timing reduced total expenditure by 22.9% (USD 1,306,306, purchasing power parity-adjusted); 75.0% of savings accrued to patients as reduced out-of-pocket payments and only 25.0% to the insurer, reflecting a mean insurance coverage rate of 27.7% for this care pathway. Freed capacity could accommodate 1,328 additional admissions without infrastructure expansion. Public hospital patients and those aged 60 years and above showed the greatest benefit, with out-of-pocket reductions of 43.8% and 35.5% respectively. The strongest predictor of prolonged stay was payer-classification status rather than any clinical variable, revealing a structural misalignment between insurance administration and discharge practice.

**Conclusion:**

Machine learning applied to routine insurance claims can reliably flag patients for clinical discharge review and quantify multi-dimensional system impacts. Discharge optimization in typhoid care functions primarily as a patient financial protection intervention, and should be prioritized in public hospitals and among elderly patients where financial vulnerability and optimization potential are both greatest.

## Introduction

Typhoid fever, caused by Salmonella enterica serovar Typhi, remains one of the most consequential yet preventable infectious diseases globally, imposing a disproportionate burden on low- and middle-income countries (LMICs) where fragile sanitation systems, diagnostic delays, and fragmented health infrastructure converge to sustain endemic transmission [1]. Approximately nine million new cases occur annually, resulting in around 110,000 deaths, with the highest incidence concentrated in South Asia, sub-Saharan Africa, and Oceania—regions where case rates can exceed 300 per 100,000 population [2–5]. While advances in vaccination and antimicrobial therapy have partially contained mortality, hospitalization rates remain stubbornly high, generating substantial pressure on already constrained health system resources. In endemic settings, typhoid admissions are not merely a clinical problem; they are a systems problem—consuming bed capacity, exhausting insurance budgets, and exposing deep structural inequities in how care is financed and delivered [6].

In Iran, national typhoid incidence has declined from 0.85 per 100,000 in 2010 to 0.5 per 100,000 in 2014, yet the disease remains endemic in several provinces—particularly those bordering Afghanistan and Pakistan—where transboundary migration, contaminated water sources, and inadequate sanitation sustain transmission [7,8]. Epidemiological data reveal marked vulnerability among children under 15 years, who accounted for 31% of reported cases in 2010, alongside women in caregiving roles and rural residents, who represented 56% and more than 53% of cases respectively [7,9]. These demographic patterns reflect deep socioeconomic gradients in exposure risk and healthcare access. Patients in rural settings face significant diagnostic delays, are referred later to specialist facilities, and ultimately remain hospitalized for longer periods than their urban counterparts—translating directly into greater financial burden and higher mortality [8]. Against this epidemiological backdrop, the Iranian health financing system compounds inefficiency: multiple insurance funds—including the Social Security Organization (SSO), Iran Health Insurance Organization (IHIO), armed forces medical services insurance organization, and the Imam Khomeini Relief Foundation—operate with minimal cross-subsidization, producing benefit inequities, reimbursement unpredictability, and fragmented care pathways [10,11]. Hospital payment under a predominantly fee-for-service (FFS) model creates structural incentives for prolonged admissions, while diagnosis-related group (DRG) reforms remain inconsistently implemented, leaving hospitals with limited motivation to optimize discharge timing [12,13].

A critical and unresolved challenge within this landscape is the absence of data-driven, evidence-based frameworks for optimizing the timing of patient discharge in typhoid hospitalizations. Discharge decisions in routine clinical practice are largely clinician-dependent, subject to institutional inertia, and rarely calibrated against objective prediction of recovery trajectories or downstream resource utilization. Studies to date have examined length-of-stay (LOS) determinants in typhoid as descriptive epidemiological exercises, without translating these findings into actionable discharge optimization tools [6,7]. Machine learning (ML) methods, and gradient-boosted ensemble models in particular, have demonstrated superior predictive performance for clinical event classification across a range of inpatient conditions—yet their systematic application to discharge timing in infectious disease, and specifically in resource-constrained LMICs with fragmented insurance systems, remains largely unexplored [14,15]. Furthermore, while the clinical utility of ML-based predictive models has attracted growing attention, the downstream financial and systemic consequences of model-guided discharge decisions have rarely been quantified in a unified analytical framework [16]. Studies typically report model performance in isolation, leaving policymakers without actionable estimates of what optimized discharge timing would mean for bed-day availability, insurance expenditure, or distributional equity across hospital types and patient subgroups.

This gap carries substantial consequences. In a health system where FFS incentives already promote over-hospitalization, the absence of objective discharge guidance systematically inflates bed-day consumption, delays patient throughput, and increases both insurer liability and patient out-of-pocket (OOP) expenditure—particularly for rural and lower-income populations who are least able to absorb excess costs [11,12]. At the systemic level, inefficient discharge practice reduces the capacity of departments to admit new patients during seasonal surges, exacerbates hospital-level resource inequities between public and private facilities, and compounds the fiscal fragmentation characteristic of Iran’s multi-payer insurance architecture [10,13]. Quantifying these harms with precision—across clinical, financial, and systemic dimensions simultaneously—is a prerequisite for credible policy reform. Without such evidence, advocacy for discharge optimization remains rhetorical.

This study addresses these gaps through an integrated, three-level analytical framework applied to a large national cohort of typhoid inpatient admissions in Iran. Using LightGBM—a high-performance gradient-boosted decision tree algorithm—we develop and validate a prediction model to identify patients at risk of prolonged hospitalization and derive individual optimal discharge timing. We then translate model outputs into quantified clinical, financial, and systemic impact. To our knowledge, this is the first study to unite ML-guided discharge prediction with simultaneous clinical, financial, and equity-level impact quantification in typhoid care, and one of very few to do so in the context of a fragmented, multi-payer LMIC health system. The findings are intended to provide a directly policy-applicable evidence base for discharge optimization, insurance reimbursement reform, and equitable resource allocation in typhoid-endemic settings.

## Methods

### Data source

This study relied on a nationally representative administrative dataset obtained exclusively from the Iran Health Insurance Organization (IHIO), the principal public payer covering rural populations, self-employed individuals, government workers, and affiliated demographic groups across all provinces of Iran. The dataset comprised inpatient insurance claims corresponding to typhoid fever hospitalizations, drawn from annual records spanning 2024–2025 and was accessed on 15/05/2025 for research purposes. In its raw form, the dataset contained 80,223 claim-level records, wherein each row represented a discrete insurance claim rather than a discrete patient admission. Because a single hospitalization episode may generate multiple claims across different billing dates, service categories, or cost components, the raw claim structure was not analytically equivalent to a patient-level record—a distinction of fundamental importance in health economic analyses, where the unit of interest is the hospitalization episode and its associated cost and length-of-stay.

### Data preprocessing

#### Variable standardization and temporal feature derivation.

Raw column labels were first harmonized into a consistent analytical schema, correcting for trailing whitespace, transliteration inconsistencies, and variant spellings present in the original administrative extract. Admission and discharge dates were parsed and used to derive length of stay (LOS) directly from the interval between recorded dates, providing a date-anchored LOS estimate independent of the administratively recorded LOS field. Where the two measures diverged by more than one day—a discrepancy attributable to overnight admission conventions or administrative rounding—the date-derived value was treated as authoritative. Temporal covariates were subsequently constructed, including admission year, calendar month, day of week, and a binary indicator for weekend admission, the latter being relevant to discharge planning given documented weekend-effect patterns in hospital throughput.

#### Missing data management.

Missing values were addressed through a strategy differentiated by variable type and analytical role. For low-cardinality categorical variables—including department type, discharge status, gender, hospital ownership, insurance fund, physician specialty, and service group—modal imputation was applied, a procedure appropriate when missingness is non-systematic and the dominant category reflects structurally determined administrative defaults. For financial variables such as deductible amounts, terminally ill patient cost shares, and preferential currency components, missing entries were assigned a value of zero, consistent with their interpretation as applicable only when specific billing conditions are triggered; absence therefore denotes inapplicability rather than data loss. Age, where missing, was imputed using the cohort-wide median. Records missing critical analytical fields—LOS, total cost, or date fields—were excluded from further analysis.

#### Outlier detection and treatment.

Outlier bounds were estimated using an interquartile range (IQR) method with a conservative multiplier of 3.0, selected to accommodate the right-skewed distributions characteristic of clinical LOS and healthcare cost data while avoiding aggressive trimming of clinically plausible extreme values. Hard physiological and financial floors were enforced simultaneously: LOS and all cost components were constrained to be non-negative, and age was bounded between 0 and 120 years. Values exceeding the derived upper fence were winsorised—that is, capped at the fence value rather than deleted—thereby preserving all observations while limiting the undue leverage of extreme outliers on regression and machine learning estimates. Same-day discharges (LOS = 0) were retained in the dataset but flagged with a binary indicator, enabling their separate analysis as a clinically and administratively distinct subgroup.

#### Logical consistency validation.

A series of claim-level consistency checks were applied to enforce financial and temporal coherence. Records in which the discharge date preceded the admission date were removed as irrecoverable data errors. Cases in which the insured person’s share exceeded the total claim cost were corrected by capping the patient share at the total, preventing economically implausible cost decompositions. Similarly, instances where the organization’s paid amount exceeded its requested amount beyond a 1% tolerance threshold—reflecting minor rounding artefacts—were corrected by equating paid to requested. Residual negative values in any financial field were floored at zero. These corrections ensured that the financial architecture of each claim satisfied the fundamental payer decomposition identity: total cost = insurer share + patient out-of-pocket share + deductible + supplementary components.

#### Claims aggregation to hospitalization episodes.

Because each hospitalization episode generated multiple itemized claims in the raw data, analytical validity required aggregation to a single episode-level record. This step is methodologically essential in health economic analyses of insurance data: conflating claim counts with patient counts would overestimate resource utilization, distort LOS distributions, and produce double-counted cost estimates—each a source of systematic bias in cost-effectiveness and bed-day analyses. Aggregation was performed by grouping on the combination of patient-identifying and episode-defining fields (admission date, discharge date, age, gender, insurance fund, hospital, and department), then summing all financial components—total cost, insurer paid share, organization requested and paid amounts, patient deductible, and out-of-pocket contributions—across claims within each episode. Non-additive attributes (department type, ownership, discharge status, physician specialty) were resolved by retaining the modal value within each group, consistent with attributing the primary service category to the dominant billing component. Following aggregation, the analytical cohort comprised 13,105 unique hospitalization episodes, representing the unit of analysis for all subsequent modelling and impact quantification.

#### Ethics approval and consent to participate.

This study used secondary, non-identified administrative healthcare claims data obtained from the Iran Health Insurance Organization (IHIO). No direct contact, intervention, or interaction with human participants was involved. All data were fully anonymized prior to access and analysis, and the research team had no means of identifying individual patients.

Although the study did not involve human subjects in the conventional experimental sense, ethical approval was obtained due to the sensitive nature of healthcare administrative data. The study protocol was reviewed and approved by the School of Public Health and Allied Medicine at Tehran University of Medical Sciences under the ethical approval code IR.TUMS.NIHR.REC.1403.015.

All analyses were conducted in accordance with relevant institutional guidelines and regulations, and the principles of the Declaration of Helsinki were strictly followed throughout the study.

Given the retrospective nature of the study and the use of anonymized administrative data, the requirement for informed consent was waived by the Tehran University of Medical Sciences National Institute for Health Research Ethics Committee.

### Variables

#### Initial (Raw) features.

Research analysts utilized administrative and clinical data from inpatient insurance claims to develop decision models for the treatment and hospitalization of typhoid. A summary of the original raw features from the dataset is presented in Table 1 prior to the implementation of feature engineering.

**Table 1:** Overview of patient-level and hospital administrative features in the typhoid dataset.

Feature Name	Description
**Gender**	Patient’s sex (male/female)
**Admission Date**	Date of hospital admission
**Discharge Date**	Date of hospital discharge
**Age**	Patient’s age at the time of admission
**Total Cost**	Total expenditure incurred during hospitalization
**Insured’s Share**	Portion of cost paid directly by the insured individual
**Deductible Amount**	Fixed amount borne by the patient before insurance coverage applies
**Organization Requested Share**	Total amount requested for reimbursement from the insurance organization
**Organization Paid Share**	Amount actually reimbursed by the insurance organization
**Length of Stay (LOS)**	Total number of days the patient remained hospitalized
**Department Type**	Type of hospital department (e.g., internal medicine, infectious diseases)
**Discharge Status**	Final condition at discharge (e.g., recovered, referred, deceased)
**Diagnosis Code**	ICD code for typhoid diagnosis
**Diagnosis Type**	Primary or secondary diagnosis
**Insurance Fund**	The specific fund or plan providing the insurance coverage
**Treating Physician Specialty**	Specialty of the attending physician (e.g., internal medicine)
**Ownership**	Type of hospital ownership (public, private, or charity)
**Service Group**	Category of services received (e.g., lab, imaging, medication)

Table 1 provides an overview of the original patient-level and hospital administrative features extracted from the Typhoid claims dataset.

The correlation structure of all patient-level and hospital administrative variables is presented in S1 Table in S1 Appendix. To assess multicollinearity, we calculated variance inflation factors (see S2 Table in S1 Appendix).

#### Feature engineering.

A set of derived analytical variables was constructed to support both the predictive modelling and the health economic impact analyses. Cost efficiency was operationalized as cost per inpatient day (total cost divided by LOS), providing a normalized metric for comparing resource intensity across episodes of differing duration. Insurance performance was captured through a coverage ratio, defined as the proportion of the organization’s requested amount that was actually reimbursed, serving as an indicator of payer-level financing reliability. Patient financial burden was quantified through an out-of-pocket (OOP) composite—the sum of the insured person’s direct share and any deductible levied—expressed both in absolute terms and as a proportion of total cost. Age was categorized into four clinically and epidemiologically meaningful strata: infants and young children (0–5 years), older children and adolescents (6–17 years), working-age adults (18–59 years), and elderly patients (60 years and above). LOS was similarly stratified into five duration bands to facilitate stratified analyses and policy-relevant comparisons across care intensity levels. Table 2 summarizes the engineered features derived from administrative claims data and used in the machine learning–based optimization of typhoid discharge timing.

**Table 2:** Engineered features derived from administrative claims data for machine learning–based optimization of typhoid discharge timing.

Feature Name	Derived From	Description	Policy & Outcome Relevance	Why This is Useful for Modeling Typhoid
**Age group**	Age	Categorized age bands (infant, child, young adult, adult, elderly)	Identifies clinically vulnerable groups with potentially longer recovery	Typhoid severity and complication risk vary by age; supports equity and subgroup policy analysis
**Age group ord**	Age group	Ordinal encoding of age categories (0–4 scale)	Allows monotonic modelling of age-related risk	Improves ML performance while preserving clinical ordering
**Admission month**	Admission date	Month of hospital admission (1–12)	Captures seasonal outbreaks and resource pressure	Typhoid incidence is often seasonal; supports systemic capacity planning
**Admission weekday**	Admission date	Day of week of admission (0–6)	Reflects staffing and weekend resource constraints	Weekend admissions may influence discharge efficiency
**Admission weekend**	Admission weekday	Binary flag for weekend admission	Identifies structural discharge delays	Tests system-level bottlenecks
**year**	Admission date	Admission year (2024 vs 2025)	Controls for policy/cost changes across years	Accounts for structural reforms or reimbursement adjustments
**Same day flag**	LOS	Binary flag for zero-day stay	Separates observation-level heterogeneity	Distinguishes outpatient-like cases from true inpatients
**Cost per day**	Total cost/ LOS	Normalized daily hospital expenditure	Links clinical stay duration to financial burden	Avoids multicollinearity while capturing intensity of resource use
**Patient oop**	Insured share + Deductible	Total patient out-of-pocket payment	Measures financial protection burden	Central to financial outcome analysis
**Coverage ratio**	Org paid/ Total cost	Insurance reimbursement share (0–1)	Evaluates insurance efficiency	Directly connects to financial impact and equity
**Deductible ratio**	Deductible/ Total cost	Share of total cost borne before coverage	Reflects cost-sharing policy design	Allows modelling of patient burden under different discharge scenarios
**Pref currency flag**	Preferential currency requested	Binary flag for preferential currency support	Indicates financial vulnerability	Identifies economically sensitive patient groups
**Terminal flag**	Terminal requested	Binary indicator of terminal status	Controls for unavoidable prolonged LOS	Prevents bias in discharge optimization
**Is private**	Ownership	Binary hospital ownership (private vs public)	Tests structural efficiency differences	Supports systemic comparison and policy targeting
**Dept surgical flag**	Department type	Binary indicator for surgical departments	Captures complexity of treatment	Different departments have different LOS norms
**Specialty group**	Physician specialty	Grouped physician category (internal, infectious, pediatrics, GP, other)	Reflects treatment pathway and expertise	May influence recovery speed and discharge timing
**Is package**	Service group	Binary flag for package-based billing	Tests reimbursement model influence	Links payment structure to LOS incentives
**Gender binary**	Gender	Binary encoding (woman = 1)	Enables equity assessment	Detects gender disparities in LOS or cost burden
**Insurance fund (OHE)**	Insurance fund	One-hot encoded insurance categories	Allows fund-level efficiency comparison	Identifies variation in discharge patterns by insurer
**Discharge status (OHE)**	Discharge status	One-hot encoded discharge outcomes	Controls for clinical endpoint variation	Separates recovered vs referred vs deceased in modelling prolonged stay
**Specialty group (OHE)**	Specialty group	One-hot encoded specialty groups	Allows non-linear specialty effects	Enhances predictive flexibility in LightGBM
**Prolonged stay (Target)**	LOS (75th percentile threshold)	Binary: LOS above 75th percentile	Core optimization outcome	Enables classification-based discharge policy modelling

Table 2 summarises the engineered clinical, financial, and systemic features derived from raw administrative claims data to support the LightGBM-based prediction of prolonged hospital stay and subsequent discharge optimization analysis.

#### Research design and analytical framework.

This study employs a retrospective observational study design with an embedded computational modelling component. The analytical approach is grounded in the positivist tradition, wherein model-derived predictions are treated as estimates of counterfactual discharge outcomes that can be evaluated against empirically observed data. The overarching framework is consistent with economic evaluation methods established in the health technology assessment literature, specifically the decision-analytic tradition of projecting economic outcomes from clinical decision rules [17].

The study operationalizes a three-level cascade of outcomes — clinical, financial, and systemic — directly corresponding to the three research objectives articulated in the introduction. This nested structure is motivated by Papanicolas and Smith’s (2013) taxonomy of health system performance dimensions, which identifies efficiency, equity, and financial protection as analytically separable but causally linked outputs of hospital-level decision-making [18]. Discharge timing was selected as the intervention point because it sits at the intersection of clinical judgement and administrative incentives, making it simultaneously modifiable and policy-relevant in the context of Iran’s fee-for-service payment environment.

The study design proceeds through five sequential analytical steps: (i) development and validation of a LightGBM binary classification model to predict prolonged hospitalization; (ii) quantification of clinical impact in terms of bed-days freed and capacity reallocation potential; (iii) financial impact analysis disaggregated by payer; (iv) systemic impact analysis including distributional equity measurement; and (v) multi-dimensional sensitivity analysis to test robustness of primary findings. All analyses were conducted at the hospitalization episode level following claim aggregation.

#### Outcome variable definition.

The primary outcome variable was a binary indicator of prolonged hospitalization, defined as a length of stay (LOS) of three or more days (LOS >= 3). This threshold was selected on the basis of three converging considerations. Clinically, uncomplicated typhoid fever responding to appropriate antimicrobial therapy does not routinely require more than 48 hours of inpatient observation prior to safe discharge, consistent with WHO clinical management guidelines and Iranian infectious disease practice standards [19,20]. Statistically, the threshold was the lowest clinically defensible cut-point that yielded at least 5% positive cases — a minimum required for stable stratified cross-validation — assessed iteratively across candidate thresholds of 3, 5, and 7 days. Analytically, the LOS >= 3 threshold produced a class prevalence sufficient to support gradient-boosted ensemble modelling without requiring synthetic oversampling, which would have introduced distributional artefacts into a dataset with a discrete, bounded LOS distribution (ranging 0–4 days in the inpatient cohort).

For the downstream impact analyses, the optimal LOS for each predicted prolonged case was set at the threshold value minus one day (i.e., optimal LOS = 2 days for LOS >= 3 threshold), representing the maximum clinically justifiable reduction. A minimum inpatient floor of one day was enforced across all inpatient admissions (LOS > 0) in recognition that any admitted patient necessarily consumes at least one unit of inpatient resource. Same-day discharges (LOS = 0) were retained in the descriptive cohort but excluded from savings analyses, as no inpatient LOS reduction was applicable to this subgroup.

### Machine learning model: LightGBM binary classifier

#### Algorithm selection and justification.

The prediction model was implemented using the Light Gradient Boosting Machine (LightGBM) algorithm, a variant of gradient-boosted decision trees introduced by Ke et al. (2017) [21]. LightGBM was selected over alternatives including logistic regression, random forest, and deep learning architectures on the basis of four criteria relevant to this study context. First, LightGBM employs a leaf-wise tree growth strategy with histogram-based feature binning, which delivers superior predictive performance on moderate-sized tabular datasets relative to level-wise competitors such as XGBoost [21,22]. Second, its native handling of missing values via surrogate splits makes it robust to the incomplete administrative data characteristic of insurance claims databases [23]. Third, LightGBM is directly interpretable through SHAP (SHapley Additive exPlanations) decomposition, enabling attribution of predictions to individual features consistent with the transparency requirements of health economic applications [24]. Fourth, computational efficiency at the scale of this dataset (13,105 episodes, > 20 features) makes full cross-validated hyperparameter evaluation feasible without distributed infrastructure.

Two comparator models were trained to establish a performance baseline: a majority-class dummy classifier (representing the no-information rate) and a regularized logistic regression with balanced class weights (representing the best-performing classical statistical classifier for binary health outcomes). All three models were evaluated using identical cross-validation procedures, ensuring that performance comparisons are attributable to algorithmic differences rather than evaluation methodology.

#### Feature selection and leakage prevention.

Feature selection was governed by a strict anti-leakage protocol. Variables that encode LOS directly or that are determined simultaneously with LOS — including cost-per-day, same-day flag, coverage ratio, and all financial components correlated with LOS at r >= 0.40 — were excluded from the feature matrix. This exclusion is essential because their inclusion would allow the model to learn a trivial LOS-to-LOS mapping rather than the clinically meaningful admission-time predictors of prolonged stay [25,26]. Exclusion decisions were informed by Pearson correlation analysis between candidate features and LOS, and by clinical domain knowledge regarding the temporal ordering of measurement acquisition.

The final leakage-free feature set comprised patient-level attributes (age, gender, age group ordinal encoding), episode-level administrative variables (insurance fund membership, hospital ownership, department type, service group, physician specialty, discharge status), temporal covariates (admission year, calendar month, day of week, weekend admission indicator), and binary flags derived from structural billing properties. All categorical features were label-encoded prior to modelling.

#### Model training, hyperparameters, and cross-validation.

Model performance was estimated using stratified 5-fold cross-validation, which preserves the positive-class prevalence across folds and prevents optimistically biased performance estimates arising from random imbalanced splits [27]. Out-of-fold (OOF) predicted probabilities were aggregated across all five held-out folds to produce a single set of OOF predictions spanning the full dataset, which were subsequently used in all downstream impact calculations.

The LightGBM model was trained with the following hyperparameters: 500 estimators, learning rate 0.05, maximum tree depth 6, number of leaves 31, minimum child samples 20, feature subsampling ratio 0.8, and row subsampling ratio 0.8. The scale_pos_weight parameter was set to the ratio of negative to positive class observations to address class imbalance without synthetic resampling, consistent with recommendations by He and Garcia (2009) for moderate imbalance ratios. All hyperparameters were set a priori based on established guidance for gradient-boosted classifiers applied to moderate-n administrative health data rather than through automated grid search, avoiding the risk of hyperparameter overfitting on the available sample [28,29].

Missing values within the feature matrix were imputed using median imputation applied within each cross-validation fold — fitted exclusively on the training fold and applied to the validation fold — to prevent information leakage from the validation set into the imputation procedure. The logistic regression comparator additionally underwent standardization using a zero-mean unit-variance scaler fitted within each fold. A concise summary of the core hyperparameters and training settings used across the three modelling approaches is presented in Table 3.

**Table 3:** The key model configuration parameters.

Parameter	LightGBM	Logistic Regression	Dummy Classifier
**Algorithm type**	Gradient-boosted trees (leaf-wise)	Penalized linear (L2, saga solver)	Majority class baseline
**Estimators/ max iterations**	500 trees	2000 iterations	N/A
**Learning rate/ regularization**	0.05	C = 1.0 (default)	N/A
**Max depth/ leaves**	6/ 31	N/A	N/A
**Min samples per leaf**	20	N/A	N/A
**Subsampling (row/ col)**	0.8/ 0.8	N/A	N/A
**Class imbalance correction**	scale_pos_weight = neg/pos ratio	class_weight = balanced	N/A
**Missing value handling**	Median imputation (per-fold)	Median imputation (per-fold)	Median imputation
**Cross-validation**	5-fold stratified OOF	5-fold stratified OOF	5-fold stratified OOF
**Random seed**	42	42	42

Table 3. Model configuration summary.

#### Model evaluation metrics.

Model performance was quantified using seven metrics computed on OOF predictions, representing distinct facets of classifier quality: (i) Area Under the Receiver Operating Characteristic Curve (AUC-ROC), measuring overall discriminative ability; (ii) Average Precision (AUC-PR), measuring performance on the imbalanced positive class; (iii) Accuracy; (iv) Precision; (v) Recall (Sensitivity); (vi) F1-Score, the harmonic mean of Precision and Recall; and (vii) Brier Score, measuring probabilistic calibration. Each metric was computed per fold and reported as mean + /- standard deviation across the five folds to characterize estimation uncertainty.

The primary discrimination metric was AUC-ROC, for which 95% bootstrap confidence intervals were derived from 2,000 resampled iterations of the OOF prediction vector. AUC-PR was treated as the secondary discrimination metric given its sensitivity to class imbalance; it penalizes models that achieve high AUC-ROC through specificity gains at the expense of positive-class recall [30]. Threshold sensitivity was evaluated across a range of 0.30 to 0.70 in increments of 0.05, reporting Precision, Recall, F1, and the percentage of patients flagged for early discharge at each threshold, to enable policymakers to select a threshold consistent with their risk tolerance for false positives (early discharge of patients who would benefit from continued admission) versus false negatives (retained admission of patients who could safely be discharged).

Model calibration — the agreement between predicted probabilities and observed event rates — was assessed using reliability diagrams partitioned into ten equal-width bins, and quantified using Expected Calibration Error (ECE), defined as the weighted average of absolute differences between mean predicted probability and observed frequency across probability bins. Post-hoc calibration correction was performed using Platt scaling (logistic regression fitted to predicted_proba as input, true outcome as target), applied via 5-fold cross-validation to prevent overfitting. Isotonic regression was evaluated as an alternative but rejected because it reduced bed-days freed by more than 95%, indicating severe overfitting on the 18.2% positive class. The Brier Score before and after calibration was reported in supplementary materials.

#### Explainability: SHAP analysis.

Model explainability was assessed using SHapley Additive exPlanations (SHAP), a game-theoretic framework that decomposes each model prediction into additive contributions from individual features, satisfying the axioms of efficiency, symmetry, dummy, and linearity [31,32]. SHAP values were computed on the full training dataset using the TreeExplainer method, which exploits the ensemble structure of gradient-boosted trees to compute exact Shapley values in polynomial rather than exponential time [24]. Global feature importance was characterized by mean absolute SHAP value across all observations; local feature impact patterns were visualized using beeswarm plots showing the distribution of SHAP values for each feature. Dependence plots were produced for the four highest-importance features to characterize non-linear and interaction effects.

#### Clinical impact analysis.

The clinical impact analysis quantifies the potential reduction in inpatient bed-day consumption attributable to model-guided discharge optimization. For each patient i predicted as prolonged-stay (predicted_prolonged = 1), the optimal LOS was defined as:


$$\begin{array}{c} Optimal\_LOS(i)\;=\;min(max(LOS\_threshold\;-\;1,\;LOS\_floor),\;actual\_LOS(i)) \end{array}$$ (1)

where LOS_threshold = 3 days (primary analysis), LOS_floor = 1 day (minimum inpatient stay), and actual_LOS(i) is the observed LOS of patient i. For patients predicted as non-prolonged (predicted_prolonged = 0), optimal_LOS(i) = actual_LOS(i), implying no change to discharge timing. Bed-days freed for patient i were computed as:


$$\begin{array}{c} Days_{freed\left(i\right)}=\;max\left(actual_{LOS\left(i\right)}-\;optimal_{LOS\left(i\right)},\;0\right) \end{array}$$ (2)

Aggregate bed-days freed and the proportion of inpatient capacity released were computed as cohort-level sums. The potential increase in patient throughput — the number of additional patients who could be admitted into the freed capacity — was estimated as:


$$\begin{array}{c} Additional\_patients\;=\;total\_days\_freed\;/\;mean\_LOS\_all\_inpatients \end{array}$$ (3)

The denominator uses the mean LOS of all inpatients (not only eligible patients) because freed beds are available to any new admission, not exclusively those previously occupying prolonged-stay beds. This follows the standard capacity modelling convention in health operations research [33,34]. Bootstrap 95% confidence intervals (5,000 resamples) were computed for mean bed-days saved per eligible patient using the percentile method [35].

Subgroup analyses were conducted by hospital ownership (public versus private), insurance fund, age group, and calendar month of admission. For each subgroup, the same metrics were computed alongside the proportion of inpatient capacity freed and the number of additional patients possible.

### Financial impact analysis

#### Cost data and scope.

Financial analysis was restricted to the inpatient episode cohort (LOS > 0; n = 5,784) because same-day encounters generate no reducible inpatient cost under a discharge optimization intervention. Total episode cost was reconstructed from the payer decomposition present in the administrative claims:


$$\begin{array}{c} Total\_cost(i)\;=\;org\_paid(i)\;+\;patient\_OOP(i) \end{array}$$ (4)

where org_paid(i) is the amount reimbursed by the insurance organisation and patient_OOP(i) is the patient out-of-pocket share, itself comprising the patient’s direct liability and any applicable deductible. This decomposition follows the payer identity convention used in Iranian health financing research [36]. Insurance coverage was characterized by the coverage ratio:


$$\begin{array}{c} Coverage\_ratio(i)\;=\;org\_paid(i)\;/\;total\_cost(i)\;\left[bounded\;to\;\left[0,\;1\right]\right] \end{array}$$ (5)

#### Cost projection method.

Projected costs under optimized discharge were estimated using a ratio-based projection method, which avoids the linearity assumption that each additional day costs a fixed marginal amount — an assumption empirically invalidated by the bundled billing structure documented in this dataset [17]. The method proceeds as follows. For each inpatient episode i with days_freed(i) > 0:


$$\begin{array}{c} Optimal\_cost(i)\;=\;total\_cost(i)\;x\;(optimal\_LOS(i)\;/\;actual\_LOS(i)) \end{array}$$

$$\begin{array}{c} Cost\_saved(i)\;=\;total\_cost(i)\;-\;optimal\_cost(i) \end{array}$$ (6)

The LOS ratio (optimal_LOS/ actual_LOS) acts as a scalar that reduces total cost proportionally to the fraction of stay saved, while remaining agnostic to the internal billing structure of the episode. This method is consistent with the resource reduction approach recommended by Drummond et al. (2015) for situations where marginal daily costs are not separately identifiable in administrative data [17].

Payer-specific savings were obtained by applying the individual coverage ratio and OOP ratio of each episode to the total cost saved:


$$\begin{array}{c} Insurance\_savings(i)\;=\;cost\_saved(i)\;x\;coverage\_ratio(i) \end{array}$$

$$\begin{array}{c} OOP\_savings(i)\;=\;cost\_saved(i)\;x\;OOP\_ratio(i) \end{array}$$ (7)

This approach preserves each patient’s actual payer mix rather than applying a population-average coverage assumption, which would misrepresent the distributional impact across insurance funds with heterogeneous benefit packages. The primary analysis applied a minimum inpatient LOS floor of one day (sensitivity: two days). Sensitivity to the PPP conversion rate was assessed across a range of plus or minus 30% around the base rate, recognizing Iran’s documented currency volatility [37]. Bootstrap 95% confidence intervals were derived for all per-patient savings estimates using 5,000 resamples with the percentile method.

### Systemic impact analysis

#### Resource utilization and capacity reallocation.

Systemic resource impact was assessed at the department-type level, the hospital ownership level, and the service group level. For each stratum, the following metrics were computed: total current inpatient patient-days; total optimized patient-days; absolute bed-days freed; percentage of inpatient capacity freed; number of patients eligible for earlier discharge; the Resource Pressure Index (RPI), defined as the ratio of total patient-days to total episodes within the stratum; and the number of additional patients who could theoretically be admitted into the freed capacity.

A capacity reallocation simulation was conducted to estimate the redistributive potential of freed capacity from lower-utilization departments to higher-utilization ones. Departments were classified as high-pressure or low-pressure based on their RPI relative to the cohort-wide median RPI. The donor pool for reallocation consisted exclusively of bed-days freed from low-pressure departments. These were allocated to high-pressure departments in proportion to each high-pressure department’s share of total high-pressure patient-days:


$$\begin{array}{c} reallocated\_days(d)=donor\_pool\;x\;[patient\_days(d)/sum\;of\;patient\_days\;across\;high-pressure\;departments] \end{array}$$ (8)

The additional patients accommodatable from reallocation were then estimated as reallocated_days(d)/ mean_LOS(d). This simulation is explicitly presented as a policy illustration and not as an operational projection; the reallocation of physical bed capacity requires workforce, scheduling, and infrastructure analysis beyond the scope of the present study [34,38].

#### Distributional equity analysis.

The distributional equity of inpatient patient-days across department types was assessed using the Gini coefficient, a standard inequality metric derived from the Lorenz curve [39]. The Gini coefficient ranges from 0 (perfect equality, all departments consume identical shares of total patient-days) to 1 (perfect inequality, a single department consumes all patient-days). For a set of n department types with patient-day vectors x = {x_1,..., x_n}, sorted in ascending order, the Gini coefficient is computed as:


$$\begin{array}{c} G\;=\;[2\;x\;sum(rank(i)\;x\;x\_i)\;-\;(n\;+\;1)\;x\;sum(x\_i)]\;/\;[n\;x\;sum(x\_i)] \end{array}$$ (9)

where rank(i) is the rank of x_i in the sorted distribution (1 through n). Gini coefficients were computed both for the current patient-day distribution and for the optimized patient-day distribution (replacing actual_LOS with optimal_LOS_adj), enabling direct before-versus-after comparison. Bootstrap 95% confidence intervals were derived from 2,000 resamples, with each resample drawing from individual episode-level records (rather than from department-level aggregates) to preserve the underlying sampling variability of the data.

Departments with fewer than 30 inpatient episodes were excluded from the Gini analysis as group-level patient-day estimates for small strata are unstable and would distort the Lorenz curve. The direction of Gini change was interpreted as follows: an increase in the Gini coefficient following optimization indicates that the optimization disproportionately reduces patient-days in already-dominant departments, concentrating the residual utilization in previously lower-volume ones; a decrease indicates a convergence toward more equitable utilization. The mechanism underlying any change was examined by inspecting the departmental patient-day shares before and after optimization.

#### Sensitivity analysis.

A multi-dimensional sensitivity analysis was conducted to assess the robustness of primary findings to key analytical assumptions. Sensitivity analyses are an explicit requirement of economic evaluations conducted, and are considered essential for credible policy communication in health economics [40,41].

Three structural parameters were varied systematically. First, the minimum LOS floor assumption was tested at zero days (no minimum, representing maximum feasible savings), one day (primary analysis), and two days (conservative minimum representing a 48-hour observation protocol). Second, the prediction threshold was varied from 0.30 to 0.70 in increments of 0.05, assessing the sensitivity of bed-day savings, patient eligibility, and model performance metrics to the classification boundary. Third, the PPP conversion rate was varied by plus or minus 10%, 20%, and 30% around the World Bank 2024 base rate to account for exchange rate uncertainty.

A subgroup robustness analysis assessed whether the model’s discriminative performance (AUC-ROC) and calibration (ECE, mean probability gap) were consistent across hospital ownership categories (public versus private), age groups, genders, and department types. Subgroups with fewer than 30 inpatient episodes or fewer than five positive-class cases were excluded as AUC estimates would be unreliable in these strata. Because prolonged_stay equalled one for all inpatients (confirming that the model’s target in Step 1 differed from the structural binary used in sensitivity subgroup analysis), the true binary outcome for subgroup AUC computation was derived directly as: was the actual LOS strictly greater than the optimal LOS after applying the one-day floor? This operationalization corresponds to whether a patient had a genuinely reducible stay, which is the estimated of direct policy relevance.

#### Reporting standards and software.

This study followed Reporting of a multivariable prediction model for Individual Prognosis or Diagnosis for the development and validation of clinical prediction models. Subgroup analyses were pre-specified in the analytical protocol rather than conducted post-hoc, and the primary analysis threshold and LOS floor were determined a priori before examining impact estimates. All analyses were conducted in Python 3.12. All random operations were seeded at 42 for reproducibility. Monetary values are reported in PPP-adjusted international USD unless otherwise stated.

## Results

The analysis of hospitalization episodes revealed clear patterns in discharge inefficiency, financial burden, and system-level resource use, allowing the machine learning model to quantify the multi-dimensional consequences of prolonged stays in typhoid care. Model performance, patient-level optimization potential, and downstream clinical, financial, and systemic impacts are presented in this section, with subgroup analyses highlighting where discharge optimization yields the greatest benefit. Together, these findings demonstrate how routine insurance data can be transformed into actionable evidence for improving hospital efficiency and patient financial protection.

### LightGBM model performance

Performance comparison of machine learning models for predicting extended length of stay in typhoid patients using 5-fold stratified cross-validation (n = 13,105). Metrics are reported as mean ± standard deviation across folds. Bootstrap 95% confidence intervals (CIs) for AUC-ROC and AUC-PR were derived from 2,000 resamples of the full out-of-fold prediction vectors using the percentile method. Table 4 summarizes the comparative performance of all models.

**Table 4:** Comparative model performance with 95% bootstrap confidence intervals.

Model	AUC-ROC		AUC-PR		Accuracy Mean ± SD	Precision Mean ± SD	Recall Mean ± SD	F1-Score Mean ± SD	Brier Score Mean ± SD
	Mean ± SD	95% CI	Mean ± SD	95% CI					
Dummy (majority class)	0.5000 ± 0.0000	N/A	0.4414 ± 0.0002	N/A	0.5586 ± 0.0002	0.0000 ± 0.0000	0.0000 ± 0.0000	0.0000 ± 0.0000	0.4414 ± 0.0002
Logistic Regression	0.8311 ± 0.0027	0.8237–0.8384	0.7586 ± 0.0056	0.7444–0.7712	0.7791 ± 0.0025	0.7244 ± 0.0034	0.8064 ± 0.0102	0.7631 ± 0.0039	0.1682 ± 0.0011
**LightGBM†**	**0.8622 ± 0.0012**	**0.8558–0.8682**	**0.8050 ± 0.0057**	**0.7921–0.8156**	**0.7916 ± 0.0014**	**0.7438 ± 0.0053**	**0.8053 ± 0.0100**	**0.7733 ± 0.0023**	**0.1497 ± 0.0010**

† Best-performing model. Bold values indicate the highest performance on each metric. AUC-ROC = Area Under the Receiver Operating Characteristic Curve; AUC-PR = Area Under the Precision-Recall Curve; CI = Confidence Interval. Bootstrap CIs were not computed for the Dummy classifier (majority-class baseline) as its AUC-ROC is fixed at 0.500 by construction.

Table 4 presents the comparative performance of all evaluated models across five discrimination, calibration, and classification metrics. The gradient-boosted classifier (LightGBM) achieved the highest mean out-of-fold AUC-ROC of **0.8622 (SD = 0.0012; 95% CI: 0.8558–0.8682)**, demonstrating both strong discriminatory ability and high stability across cross-validation folds (SD = 0.0012). Logistic regression achieved a mean AUC-ROC of **0.8311 (SD = 0.0027; 95% CI: 0.8237–0.8384)**, and the majority-class baseline achieved the expected AUC-ROC of 0.500.

Importantly, the 95% bootstrap confidence intervals for LightGBM (0.8558–0.8682) and logistic regression (0.8237–0.8384) do **not overlap**, providing strong visual evidence that LightGBM’s superior discrimination is not attributable to sampling variability. This non-overlap is consistent with a statistically meaningful performance advantage, a finding further confirmed by the formal DeLong test reported below.

Consistent with its superior AUC-ROC, LightGBM also achieved a higher AUC-PR of **0.8050 (95% CI: 0.7921–0.8156)** compared to logistic regression (AUC-PR = 0.7586; 95% CI: 0.7444–0.7712). The non-overlapping AUC-PR confidence intervals reinforce this advantage in detecting the minority class (extended-stay patients). LightGBM also outperformed logistic regression on accuracy (0.7916 vs. 0.7791), F1-score (0.7733 vs. 0.7631), and Brier score (0.1497 vs. 0.1682), the latter indicating better probability calibration. Bootstrap confidence intervals were derived from 2,000 resamples of the full out-of-fold prediction vectors using the percentile method. The same random seed and resampling procedure were applied consistently across all bootstrap analyses in this study.

To formally compare the discriminative performance of LightGBM and logistic regression, a DeLong test for correlated receiver operating characteristic (ROC) curves was conducted using the full out-of-fold prediction vectors of both models (n = 13,105 paired observations). The DeLong test, Table 5, is the appropriate statistical test for within-dataset AUC comparisons because both models were evaluated on the same patients, inducing a correlation between their AUC estimates that standard two-sample z-tests

**Table 5:** DeLong test comparison of AUC-ROC performance between LightGBM and logistic regression using paired out-of-fold predictions.

Comparison	AUC-ROC LightGBM	AUC-ROC Logistic Regression	AUC Difference (LGB − LR)	95% CI (Difference)	DeLong z-statistic	p-value (two-sided)
**LightGBM vs. Logistic Regression**	0.8619	0.8310	+0.0309	[0.0264, 0.0355]	13.298	< 0.0001

DeLong test comparing AUC-ROC values of LightGBM and logistic regression classifiers evaluated on the same out-of-fold (OOF) prediction vectors (n = 13,105). The DeLong test accounts for the within-subject correlation arising from both models being evaluated on the same patients. A two-sided significance level of α = 0.05 was applied. AUC-ROC = Area Under the Receiver Operating Characteristic Curve; CI = Confidence Interval; LGB = LightGBM; LR = Logistic Regression. The DeLong test was implemented following the O(n log n) algorithm of Sun & Xu (2014). OOF predictions used the identical 5-fold stratified cross-validation splits (seed = 42) as the main model evaluation.

Table 5. To formally assess whether LightGBM’s discrimination advantage over logistic regression reached statistical significance, a DeLong test for correlated receiver operating characteristic curves was performed on the full out-of-fold prediction vectors of both models (n = 13,105 paired observations; Table 5).

LightGBM achieved a significantly higher AUC-ROC than logistic regression (0.8619 vs. 0.8310; AUC difference = +0.0309, 95% CI: [0.0264, 0.0355]; DeLong z = 13.298, p < 0.0001, two-sided). The 95% confidence interval for the AUC difference excludes zero entirely, confirming that the 3.1 percentage-point improvement in discrimination is statistically meaningful and unlikely to reflect sampling variability. These findings complement the non-overlapping bootstrap confidence intervals reported in Table 4, providing convergent statistical evidence for the superiority of the gradient-boosted approach.

The magnitude of the difference (z = 13.298) is large by conventional standards, reflecting not only a statistically significant but also a practically meaningful improvement in the model’s ability to distinguish patients at risk of extended stays from those likely to be discharged within the optimal window. Given the administrative application context — where the model serves as a decision support trigger for clinical review rather than an autonomous discharge order — this improvement is operationally relevant.

A detailed evaluation of the LightGBM classifier’s discrimination between optimal-stay and prolonged-stay hospitalizations, including class-specific precision, sensitivity, F1-scores, and support counts derived from out-of-fold predictions at the primary decision threshold of 0.50, is presented in Table 6, providing a granular view of model behavior across clinically meaningful outcome categories.

**Table 6:** LightGBM classification performance by predicted class (OOF predictions, threshold = 0.50, n = 13,105).

Class	Precision	Recall (Sensitivity)	F1-Score	n (Support)
**Optimal discharge (LOS < threshold; true negative class)**	0.835	0.781	0.807	7,321
**Prolonged hospitalization (LOS ≥ threshold; true positive class)**	0.744	0.805	0.773	5,784
**Overall accuracy**	—	—	0.792	13,105
**Macro average**	0.790	0.793	0.790	13,105
**Weighted average**	0.795	0.792	0.792	13,105

Out-of-fold (OOF) predictions on the full cohort (n = 13,105 episodes). Classification threshold = 0.50. Shaded row = class of primary economic and clinical interest. Sensitivity of 80.5% for the prolonged-stay class indicates that the model successfully flags for clinical review four in five patients potentially having reducible length of stay. a false positive means a patient is flagged for discharge review without a clinically reducible stay; a false negative (predicted optimal, actual prolonged) means a patient who could benefit from earlier discharge is not flagged — the more consequential error from a health system perspective. Confusion matrix counts: TP = 4,658; FN = 1,126; FP = 1,605; TN = 5,716.

**Fig 1:**
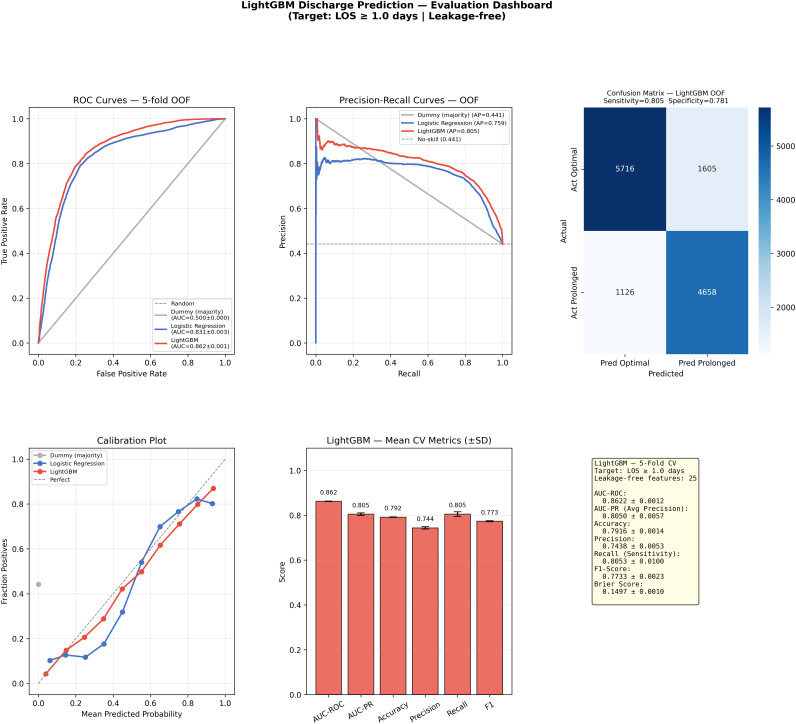
LightGBM model evaluation dashboard for LOS ≥ 1 Day (leakage-free, 5-fold OOF performance). LightGBM Discharge Prediction — Evaluation Dashboard (target: LOS ≥ 1 day, leakage-free feature set, n = 13,105). Panel A: ROC curves for all three models (5-fold OOF); LightGBM AUC-ROC = 0.862 ± 0.001. Panel B: Precision-Recall curves; LightGBM AUC-PR = 0.805 ± 0.006. Panel C: Confusion matrix at threshold = 0.50; sensitivity = 0.805, specificity = 0.781. Panel D: Calibration reliability diagrams. Panel E: Mean cross-validated metric bar chart for LightGBM (±SD). Panel F: Numerical performance summary. OOF = out-of-fold; SD = standard deviation across folds.

Table 6 presents the full per-class classification performance. At the primary classification threshold of 0.50, the LightGBM OOF classifier correctly identified 4,658 of 5,784 true prolonged-stay patients (sensitivity 80.5%) while correctly identifying 5,716 of 7,321 true optimal patients as non-prolonged (specificity 78.1%), as shown in the confusion matrix (Fig 1, panel C). These performance levels are economically meaningful: a sensitivity of 80.5% implies that four in five patients whose length of stay may be reducible are correctly flagged for clinical review, while a specificity of 78.1% limits the proportion of patients incorrectly recommended for early discharge to approximately one in five among those who actually do not require it — a false-positive rate that has tolerable clinical consequences given that typhoid inpatient care is not a high-acuity acute care setting, given that the model output triggers review rather than discharge, preserving clinical authority over the final decision.

A high-level overview of the model’s discrimination, calibration, and threshold performance is provided in Fig 1, summarizing LightGBM’s behaviour across all key evaluation panels.

Fig 1 presents the complete model evaluation dashboard for all three models. The ROC curves (panel A) confirm LightGBM’s consistent advantage across the full range of decision thresholds. The Precision-Recall curves (panel B) show that LightGBM maintains higher precision at equivalent recall levels than logistic regression — particularly important for policy implementation, as higher precision means a greater proportion of flagged patients genuinely have reducible stays. The confusion matrix (panel C) quantifies the classification errors at the primary threshold. The calibration plot (panel D) shows that while logistic regression is well-calibrated at low predicted probabilities, LightGBM’s predicted probabilities more closely track observed prolonged-stay rates across the full spectrum. The metric bar chart (panel E) provides a visual summary of all six performance dimensions.

A comprehensive ranking of feature contributions derived from the SHAP decomposition of the final LightGBM discharge-prediction model is presented in Table 7, summarizing the relative influence of all 25 administrative and clinical variables on prolonged-stay risk. This ranking provides a structured foundation for interpreting the model’s decision logic, highlighting the small subset of predictors that drive the majority of explanatory power and guiding the subsequent policy-oriented examination of the highest-impact features.

**Table 7:** SHAP feature importance ranking — LightGBM discharge prediction model (n = 13,105).

Rank	Feature	Mean |SHAP|	Health Economic Interpretation
**1**	**Preferential currency flag (pref currency flag)**	1.056	Payer classification under Iran’s foreign currency reimbursement scheme; strongest predictor, reflecting the structural role of multi-payer fragmentation in determining discharge timing.
**2**	**Patient age (age)**	0.687	Non-linear bimodal risk profile: children <15 years and elderly patients >65 years have highest prolonged-stay probability, consistent with typhoid severity epidemiology. Supports age-stratified discharge protocols.
**3**	**Surgical department flag (dept surgical flag)**	0.223	Surgical unit admission associated with shorter stays, likely reflecting throughput-oriented discharge norms in surgical wards versus conservative physician-discretionary discharge in medical wards.
**4**	**Rural insurance fund membership (insurance fund rural)**	0.201	Rural fund membership negatively associated with LOS — may reflect patient financial constraints or capacity limitations driving earlier discharge rather than superior clinical outcomes.
**5**	**Admission month (admission month)**	0.193	Captures seasonal typhoid incidence peaks and associated bed pressure effects on discharge timing.
**6**	**Weekend admission flag (admission weekend)**	0.192	Weekend admissions associated with prolonged stay, consistent with documented weekend discharge delays from reduced administrative availability.
**7**	**Package billing indicator (is package)**	0.163	Package-billed episodes associated with differential LOS patterns, reflecting bundled payment incentive structures.
**8**	**Government employee fund (insurance fund Govt)**	0.151	Government employee fund membership associated with distinct discharge timing, possibly reflecting entitlement norms and specialist access differentials.
**9**	**Age group ordinal (age group ord)**	0.119	Ordinal encoding of clinical age strata; partially redundant with continuous age but captures category-level threshold effects.
**10**	**Gender (binary; gender binary)**	0.098	Modest gender effect; female patients show slightly different discharge timing patterns, potentially reflecting caregiving role constraints and social discharge determinants.
**11–20**	**Year, private hospital, Iranians fund, referral fund, discharge status variables**	0.001–0.047	Smaller but non-trivial contributions. Year captures temporal trends in practice patterns. Hospital ownership distinguishes private vs. public discharge norms.
**21–25**	**Public health fund, terminal flag, specialty group (other), death discharge, follow-up discharge, run-away discharge**	0.000	Zero SHAP contribution: these features carry no additional predictive information beyond the retained features in the final model.

Mean |SHAP| = mean absolute SHapley Additive exPlanations value across all 13,105 episodes; higher values indicate greater average contribution to prediction. SHAP values were computed using the TreeExplainer method on the final model trained on the full dataset. Rows highlighted in blue = top two predictors of primary policy relevance. Rows 21–25 collapsed for conciseness (all with mean |SHAP| = 0.000). See Fig 1 (SHAP dependence plots, Appendix) for full graphical representations.

Table 7. SHAP (SHapley Additive exPlanations) decomposition of the final LightGBM model identified four features as the principal drivers of prolonged hospitalization prediction, together accounting for the substantial majority of mean absolute SHAP value. Table 4 presents the ranked feature importance values for all 25 model features. The four dominant features — preferential currency flag (mean |SHAP| = 1.056), age (0.687), surgical department flag (0.223), and rural insurance fund membership (0.201) — are examined in detail below, with reference to the dependence plots in Appendix.

The preferential currency flag (pref_currency_flag) was the single most influential predictor, with a mean |SHAP| more than 50% larger than the second-ranked feature. These binary variable flags episodes billed under Iran’s preferential foreign currency reimbursement mechanism, which is applied to specific patient categories including non-citizen residents and certain demographic groups. From a health economics perspective, this finding is highly significant: it implies that the payer-classification structure of the Iranian insurance system — specifically, the distinction between domestic IRR-denominated and preferential currency-denominated claims — is a stronger predictor of hospitalization duration than most clinical or demographic attributes. This aligns with the documented fragmentation of Iran’s multi-payer system, in which different payer channels are associated with systematically different facility types, service intensities, and discharge protocols. The SHAP dependence plot (Appendix Figure 1, panel A in S1 Appendix) confirms a stark binary effect: pref_currency_flag = 1 is associated with strongly positive SHAP values (increased prolonged stay probability), while flag = 0 is associated with negative values (shorter stay).

Patient age ranked second (mean |SHAP| = 0.687), with a non-linear, non-monotonic relationship to prolonged stay probability. The SHAP dependence plot (Fig 3, panel B) reveals that the highest SHAP values — greatest contribution to prolonged stay — are concentrated in children under approximately 15 years and in patients over 65 years, while working-age adults (18–59 years) show the lowest and most variable SHAP contributions. From a policy perspective, this finding suggests that age-stratified discharge protocols — with more conservative discharge criteria for children and elderly patients — would be both clinically appropriate and consistent with the model’s predictive logic.

**Fig 2:**
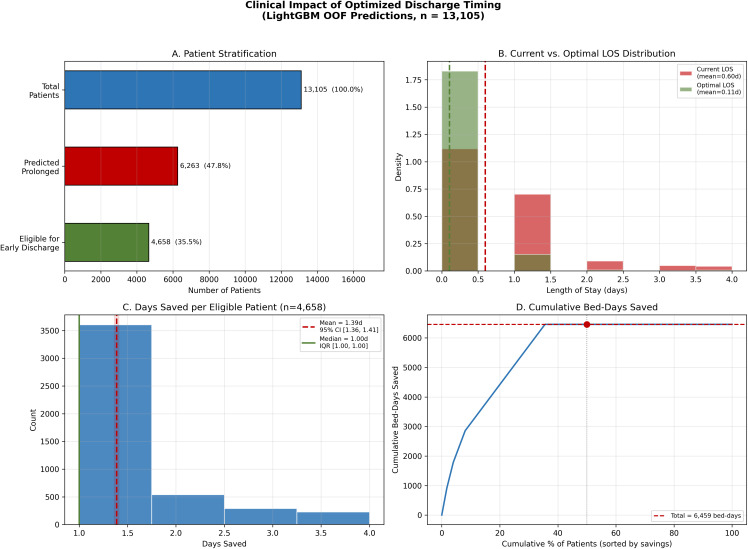
Clinical impact of optimized discharge timing. Clinical impact of optimised discharge timing among 13,105 typhoid episodes (LightGBM OOF predictions). Panel A: Patient stratification cascade showing total cohort (n = 13,105), predicted prolonged-stay patients (n = 6,263, 47.8%), and those flagged for discharge review after applying the 1-day inpatient floor (n = 4,658, 35.5%). Panel B: Density distribution of current LOS (mean 0.60 days) versus optimised LOS (mean 0.11 days) across the full cohort. Panel C: Histogram of days saved per eligible patient (n = 4,658); mean = 1.39 days (95% CI: 1.36–1.41); median = 1.00 day. Panel D: Cumulative bed-days freed curve showing that the top 50% of eligible patients (by savings) account for approximately 97% of total freed bed-days, reflecting the highly concentrated saving distribution. Total = 6,459 bed-days. OOF = out-of-fold; CI = confidence interval; IQR = interquartile range.

The surgical department flag (dept_surgical_flag; mean |SHAP| = 0.223) exerts a negative SHAP effect (flag = 1 associated with shorter stay) and a positive SHAP effect when flag = 0 (non-surgical department associated with longer stay). This counter-intuitive finding likely reflects the institutional reality that typhoid patients admitted to surgical wards are managed under discharge protocols more aligned with surgical throughput norms — typically shorter — whereas internal medicine and general wards, where most typhoid patients are managed, operate under more conservative physician-discretionary discharge practices. Rural insurance fund membership (insurance_fund_rural; mean |SHAP| = 0.201) was the fourth-ranked predictor, with rural fund membership associated with a negative SHAP contribution — that is, shorter stays — a finding that warrants careful interpretation. Rather than indicating superior health outcomes for rural patients, this may reflect earlier discharges driven by limited ability to sustain hospital costs, patient preference for community-based care, or facility-level capacity constraints that curtail inpatient observation periods in rural and peri-urban hospitals.

Admission month (mean |SHAP| = 0.193) and weekend admission flag (0.192) contributed approximately equally, reflecting seasonal typhoid incidence patterns and the documented weekend effect in hospital discharge planning. Insurance package type and government employee fund membership (SHAP ranks 7–8) further confirm the primacy of the payer structure in explaining discharge timing variation. The remaining 17 features each contributed mean |SHAP| values below 0.10, with five features contributing zero — indicating that the model’s predictive signal is concentrated in a compact, interpretable set of eight clinically and administratively meaningful predictors.

Model interpretability results are provided in the Supporting Information (S1 Appendix), including SHAP dependence plots for the top predictors of prolonged length of stay.

### Clinical impact of optimized discharge timing

A summary of the projected clinical impact of applying the optimized discharge-timing model to the full typhoid inpatient cohort is provided in Table 8, outlining the proportion of episodes flagged for review, the resulting bed-day savings, and the corresponding reductions in length of stay under the optimized scenario.

**Table 8:** Overall clinical impact of optimized discharge timing — LightGBM OOF predictions (n = 13,105).

Parameter	Value
**COHORT COMPOSITION**	
**Total episodes**	13,105
**Predicted prolonged stay (LOS ≥ threshold)**	6,263 (47.8% of cohort)
**Flagged for discharge review**	4,658 (35.5% of cohort)
**BED-DAY SAVINGS**	
**Total bed-days freed**	6,459 bed-days
**Mean days saved per eligible patient (95% CI)**	1.39 days (95% CI: 1.36–1.41)
**Median days saved per eligible patient (IQR)**	1.00 day (IQR: 1.00–1.00)
**Mean days saved across all patients (95% CI)**	0.49 days (95% CI: 0.48–0.51)
**LOS REDUCTION**	
**Mean LOS, current practice**	0.60 days
**Mean LOS, optimised scenario**	0.11 days
**Mean LOS reduction**	0.49 days (82.3% reduction)
**LOS threshold applied**	≥ 1 day (1-day inpatient floor)

All estimates are based on LightGBM out-of-fold (OOF) predictions to prevent optimistic bias. ‘Flagged for discharge review’ = predicted prolonged AND actual LOS exceeds the optimal discharge point by ≥ 1 day after applying the mandatory 1-day inpatient floor. ‘Bed-days freed’ are the primary outcome metric propagated to the financial impact analysis. Bootstrap 95% confidence intervals based on 5,000 resamples (percentile method). IQR = interquartile range.

Table 8 presents the overall clinical impact summary. Application of LightGBM out-of-fold predictions to the full cohort of 13,105 episodes identified substantial scope for discharge optimization across the typhoid hospitalization pathway. Of the total cohort, 6,263 episodes (47.8%) were predicted to involve prolonged hospitalization. After applying the one-day inpatient floor — which reflects the minimum resource consumption of any admitted episode — 4,658 patients (35.5%) were flagged for clinical discharge review, identified as potentially having reducible length of stay, defined as cases where actual LOS exceeded the optimal discharge point by at least one reducible day. The model thus distinguishes between patients correctly hospitalized beyond the threshold for clinical reasons and those whose stays may warrant clinical review for potential earlier discharge, subject to physician assessment.

Across these 4,658 eligible patients, the model projects a total of 6,459 bed-days could be freed under optimized discharge timing. This figure represents the primary outcome of the clinical impact analysis and constitutes the capacity resource that propagates through the financial and systemic impact calculations in subsequent sections. From a health economics perspective, bed-days freed represent both a direct cost reduction (through reduced hotel costs, nursing, and ancillary services per admission-day) and an indirect capacity gain (through the reallocation of freed beds to patients currently unable to access timely inpatient care — a form of opportunity cost recovery). The mean LOS across the cohort fell from 0.60 days under current practice to 0.11 days under the optimized scenario (82.3% reduction), reflecting the high concentration of same-day and very-short-stay episodes in this predominantly outpatient-adjacent typhoid population.

A visual summary of the model’s projected clinical impact across the full typhoid inpatient cohort is provided in Fig 2, illustrating how optimized discharge timing reshapes patient stratification, length-of-stay distributions, and the concentration of reducible bed-days, thereby clarifying both the operational and policy relevance of the predicted savings.

Fig 2. The concentration of savings at exactly one reducible day for the median eligible patient (IQR: 1.00–1.00) reflects the discrete, bounded structure of this study population’s LOS distribution, in which the majority of inpatient episodes involve stays of one or two days and the optimal discharge point is therefore a single-day reduction. This structural finding has a direct policy implication: the intervention required to realize the projected savings is not a complex clinical reconfiguration but a simple, systematic review of patients whose predicted risk of prolonged stay falls above the model’s decision threshold — a protocol operationally equivalent to a daily ward round flag for 35.5% of admissions.

A stratified assessment of the model’s projected clinical impact across key demographic, institutional, and payer subgroups is presented in Table 9, providing a consolidated view of how optimization potential varies by age, hospital ownership, and insurance fund. This subgroup-level decomposition highlights the heterogeneity of reducible bed-days across the Iranian multi-payer system and identifies the patient segments and facility types where targeted implementation of discharge optimization protocols would yield the greatest operational and economic benefit.

**Table 9:** Clinical impact of optimized discharge timing by patient and hospital subgroup (main manuscript).

Subgroup	n	Predicted prolonged, n (%)	Eligible, n	Eligible, %	Bed-days freed	% of total freed	Mean days saved (95% CI)	Median (IQR)
**A. By Age Group**								
**Young adult (18–39 yrs)**	3,461	2,610 (75.4%)	2,148	62.1%	2,614	40.5%	1.22 (1.19–1.24)	1.00 (1.00–1.00)
**Adult (40–59 yrs)**	3,672	1,916 (52.2%)	1,247	34.0%	1,640	25.4%	1.32 (1.27–1.36)	1.00 (1.00–1.00)
**Elderly (≥ 60 yrs)**	5,255	1,160 (22.1%)	807	15.4%	1,587	24.6%	1.97 (1.89–2.04)	2.00 (1.00–3.00)
**Child (5–17 yrs)**	495	410 (82.8%)	324	65.5%	358	5.5%	1.10 (1.06–1.16)	1.00 (1.00–1.00)
**Infant (< 5 yrs)**	200	145 (72.5%)	112	56.0%	199	3.1%	1.78 (1.54–2.01)	1.00 (1.00–2.00)
**B. By Hospital Ownership**								
**Private**	12,687	5,994 (47.2%)	4,471	35.2%	5,912	91.5%	1.32 (1.30–1.34)	1.00 (1.00–1.00)
**Public/ Other**	418	269 (64.4%)	187	44.7%	547	8.5%	2.93 (2.80–3.04)	3.00 (3.00–3.00)
**C. By Insurance Fund**								
**Government employees**	5,326	2,637 (49.5%)	1,883	35.4%	2,843	44.0%	1.51 (1.47–1.55)	1.00 (1.00–2.00)
**Iranians (non-citizen)**	2,994	1,733 (57.9%)	1,394	46.6%	1,970	30.5%	1.41 (1.37–1.46)	1.00 (1.00–1.00)
**Rural fund**	3,626	1,523 (42.0%)	1,136	31.3%	1,281	19.8%	1.13 (1.10–1.16)	1.00 (1.00–1.00)
**Other layers**	805	294 (36.5%)	186	23.1%	289	4.5%	1.55 (1.43–1.69)	1.00 (1.00–2.00)
**Other layers (referral system)**	343	76 (22.2%)	59	17.2%	76	1.2%	1.29 (1.12–1.46)	1.00 (1.00–1.00)
**Public health**	11	0 (0.0%)	0	0.0%	0	0.0%	0.00 (—)	0.00 (—)

All estimates based on LightGBM OOF predictions (threshold = 0.50; 1-day inpatient floor applied). ‘% of total freed’ = subgroup bed-days freed as a share of the 6,459 total bed-days projected for the full cohort. Bootstrap 95% CIs computed on 2,000 resamples within each subgroup. Age group thresholds: infant < 5 years; child 5–17 years; young adult 18–39 years; adult 40–59 years; elderly ≥ 60 years. Subgroups with n < 10 are excluded. ‘Iranians’ = non-citizen Iranian residents billed under the foreign preferential currency reimbursement mechanism. Public health fund row retained for completeness despite zero savings (n = 11). IQR = interquartile range; CI = confidence interval.

Table 9 presents the clinical impact of optimized discharge timing stratified simultaneously across three dimensions: age group, hospital ownership type, and insurance fund. This combined subgroup presentation allows readers to identify which patient segments drive the aggregate saving, how the intensity of optimization opportunity varies across payer and facility types, and where targeted implementation would yield the greatest benefit — all central questions for health system planning under the Iranian multi-payer structure.

By age group, young adults (18–39 years) are the single largest contributor to the aggregate saving, generating 2,614 of 6,459 bed-days freed (40.5%) despite having a moderate absolute prolonged-stay rate (75.4%). This reflects volume dominance: young adults constitute 26.4% of the full cohort but their high eligibility rate (62.1%) translates the largest absolute patient count into the largest bed-day saving. From a resource allocation perspective, this implies that discharge optimization tools would have maximum throughput impact if deployed in ward areas predominantly serving working-age adults — typically internal medicine, general wards, and infectious disease units in the Iranian hospital structure.

The elderly subgroup (≥ 60 years, n = 5,255) presents a structurally different profile. Though their eligibility rate is markedly lower (15.4%), eligible elderly patients experience a substantially longer mean reducible stay (1.97 days, 95% CI: 1.89–2.04) with a median of 2.00 days (IQR: 1.00–3.00) — the only age group with a median above 1.00 and an IQR spanning multiple days. This suggests that when elderly patients are identified as prolonged-stay, their excess hospitalization is more prolonged and more variable than in younger groups. The health economics implication is twofold: (i) per-patient savings are higher for elderly patients, increasing the cost-effectiveness of identification; but (ii) these patients also carry higher clinical complexity, meaning that the clinical decision support tool must be deployed with age-stratified discharge criteria that account for comorbidity burden and functional dependency — consistent with the bimodal SHAP age effect identified in Step 1.

The contrast between hospital ownership types is among the most economically significant findings in this section. While private hospitals dominate the cohort numerically (12,687 of 13,105 patients; 96.8%), the 418 public/other patients exhibit a dramatically higher mean days saved (2.93 days, 95% CI: 2.80–3.04) with a strikingly narrow IQR (3.00–3.00), indicating near-uniform three-day excess stays among eligible public hospital patients. This finding is consistent with the structural characteristics of public hospitals in Iran’s multi-tiered system: public facilities typically operate under greater bed pressure, reduced specialist-to-patient ratios, and more conservative discharge cultures that priorities clinical certainty over throughput. The policy implication is that per-bed-day savings are more than twice as large in public facilities, making them the priority target for discharge optimization interventions despite their smaller patient volume — a clear application of the equity-efficiency tension that characterizes resource allocation in mixed health systems.

Among insurance fund strata, the Iranians fund (non-citizen residents under the preferential currency reimbursement mechanism) exhibits the highest eligibility rate (46.6%) and a substantial mean saving (1.41 days), generating 1,970 bed-days across 2,994 patients (30.5% of total savings from 22.8% of the cohort). This over-contribution to the savings pool is consistent with the SHAP finding that preferential currency flag is the single most important predictor of prolonged stay — episodes under this billing mechanism are systematically associated with longer-than-necessary hospitalization, plausibly because reimbursement norms or clinical protocols differ between citizen and non-citizen pathways. Government employee fund members generate the largest absolute saving (2,843 bed-days) due to their dominant cohort share (40.6%), while rural fund members — despite comprising 27.7% of the cohort — contribute only 19.8% of savings, reflecting both a lower eligibility rate (31.3%) and a lower mean days-saved per eligible patient (1.13 days). This rural-urban differential in saving intensity warrants careful interpretation: rather than indicating superior discharge practices in rural settings, it may reflect access barriers (financial constraints, distance, caregiver availability) that already drive premature discharge in rural populations, leaving less residual optimization scope.

Additional analyses are provided in the Supporting Information (S1 Appendix). Stratified clinical impact estimates across patient and hospital subgroups, temporal variation in early discharge eligibility and total bed-days freed, and model calibration analyses across predicted risk deciles are presented in the Supporting Information (S1 Appendix). These analyses demonstrate variation in predicted impact across subgroups and confirm that higher predicted risk categories consistently yield greater absolute and per-patient savings.

### Financial impact of optimized discharge timing

A comprehensive summary of the projected financial consequences of implementing optimized discharge timing across the typhoid inpatient pathway is presented in Table 10, detailing total and per-patient cost savings, payer-specific impacts, and the sensitivity of these estimates to alternative minimum-stay assumptions. This table provides the system-level economic framing for the analysis, quantifying how reductions in avoidable bed-days translate into lower inpatient expenditures for both patients and insurers within Iran’s multi-payer financing structure.

**Table 10:** Overall financial impact of optimized discharge timing — Inpatient typhoid admissions (n = 5,784).

Parameter	Primary scenario (≥ 1-day floor)	Sensitivity scenario (≥ 2-day floor)
**COHORT SCOPE**		
**Total episodes**	13,105	13,105
**Same-day episodes excluded (LOS = 0)**	7,321 (55.9%)	7,321 (55.9%)
**Inpatient episodes analysed (LOS ≥ 1 day)**	5,784 (44.1%)	5,784 (44.1%)
**Eligible for cost saving (any reducible cost)**	1,055 (18.2% of inpatients)	518 (8.96% of inpatients)
**TOTAL COST COMPARISON (USD, PPP-adjusted)**		
**Total inpatient cost, current practice**	$5,714,618	$5,714,618
**Total inpatient cost, optimised scenario**	$4,408,312	$5,187,014
**Total cost saved**	$1,306,306 (22.9% reduction)	$526,800 (9.2% reduction)
**SAVINGS BY PAYER (USD, PPP-adjusted)**		
**Insurance organisation savings**	$327,135 (25.0% of total saving)	—
**Patient out-of-pocket (OOP) savings**	$979,171 (75.0% of total saving)	—
**PER ELIGIBLE PATIENT (USD, 95% CI)**		
**Mean total cost saved per eligible patient**	$1,238 ($1,127 – $1,382)	—
**Mean insurance saving per eligible patient**	$310 ($282 – $350)	—
**Mean OOP saving per eligible patient**	$928 ($844 – $1,036)	—
**PATIENT OOP BURDEN (USD, PPP-adjusted)**		
**Mean OOP per inpatient, current practice**	$736	$736
**Mean OOP per inpatient, optimized scenario**	$567	—
**Patient OOP reduction (% of baseline OOP)**	23.0%	—
**COVERAGE AND PROJECTION PARAMETERS**		
**Mean insurance coverage ratio (org_paid/ total_cost)**	27.7%	27.7%
**LOS–cost correlation (Pearson r; R²)**	r = 0.373; R² = 0.139; p < 0.001	r = 0.373; R² = 0.139; p < 0.001
**Cost projection method**	Ratio-based: optimal_cost = total_cost × (optimal_LOS/ actual_LOS)	Ratio-based (same method)

All monetary values are PPP-adjusted USD (World Bank 2024: 118,411.24 IRR per international $). ‘Eligible for cost saving’ = inpatient episodes where projected cost_saved > 0 after applying the LOS floor. The primary scenario (≥ 1-day floor) is the main estimate; the sensitivity scenario (≥ 2-day floor) tests robustness to a more conservative minimum inpatient observation requirement. Bootstrap 95% confidence intervals based on 5,000 resamples (percentile method) for per-eligible estimates. ‘—’ = per-eligible and OOP analyses were conducted for the primary scenario only; sensitivity-scenario analogues are available in Appendix Table C2. Payer-split savings are computed using each patient’s individual insurance coverage ratio, preserving heterogeneity across the multi-fund system rather than applying a population mean. OOP = out-of-pocket; LOS = length of stay; PPP = purchasing power parity.

Table 10 presents the overall financial impact summary for the primary (≥ 1-day floor) and sensitivity (≥ 2-day floor) scenarios. The financial impact analysis was restricted to the 5,784 inpatient admissions (LOS > 0; 44.1% of the full 13,105-episode cohort). The 7,321 same-day episodes (LOS = 0; 55.9%) were excluded on the grounds that no inpatient length-of-stay reduction is applicable to presentations that do not generate an inpatient bed-day. This exclusion is methodologically conservative and errs in the direction of underestimating the total saving, since same-day management optimization — though potentially significant — falls outside the scope of inpatient cost projection. A minimum one-day inpatient floor was applied to all projected optimal LOS values prior to cost calculation, reflecting the irreducible resource consumption of any formal inpatient admission. The primary finding is a projected total saving of $1,306,306 (USD, PPP-adjusted), representing a 22.9% reduction in inpatient typhoid expenditure. This saving accrues from 1,055 eligible patients (18.2% of inpatient admissions) — a smaller proportion than the 35.5% clinical eligibility rate reported in Step 2, because the financial eligibility criterion requires both a predicted prolonged stay and a non-zero reducible cost given the 1-day floor. The payer decomposition reveals a critically important equity dimension: 75.0% of total savings ($979,171) accrues to patients as reduced out-of-pocket expenditure, with only 25.0% ($327,135) accruing to the insurance organization. This 3:1 ratio reflects the Iranian base package’s mean coverage ratio of 27.7% — one of the lowest institutional coverage rates documented for a communicable disease inpatient pathway — meaning that the primary financial burden of avoidable prolonged stays falls overwhelmingly on individual patients rather than on the insurer. From a health financing equity perspective, discharge optimization is therefore primarily a patient financial protection intervention, not an insurer cost-control measure. This finding has direct implications for how the intervention should be framed in health policy discourse: the equity case for implementation rests as much on catastrophic expenditure prevention as on system-level efficiency gains.

The mean patient OOP cost per inpatient admission falls from $736 to $567 under the optimized scenario — a 23.0% reduction that, while modest in absolute USD terms, is substantial relative to the income levels of the rural and lower-income patient segments identified in the subgroup analysis. Among eligible patients, the mean per-patient saving is $1,238 (95% CI: $1,127–$1,382), decomposed into $310 (95% CI: $282–$350) for insurance and $928 (95% CI: $844–$1,036) for OOP — reinforcing that the per-patient financial benefit is heavily concentrated in OOP reduction. The sensitivity scenario (≥ 2-day floor) yields 518 eligible patients (8.96%) and a total saving of $526,800, demonstrating that the financial impact is materially sensitive to the minimum LOS floor assumption — consistent with this study population’s bounded LOS distribution (maximum 4 days) — and underscoring the importance of the primary scenario as the more clinically defensible estimate.

**Fig 3:**
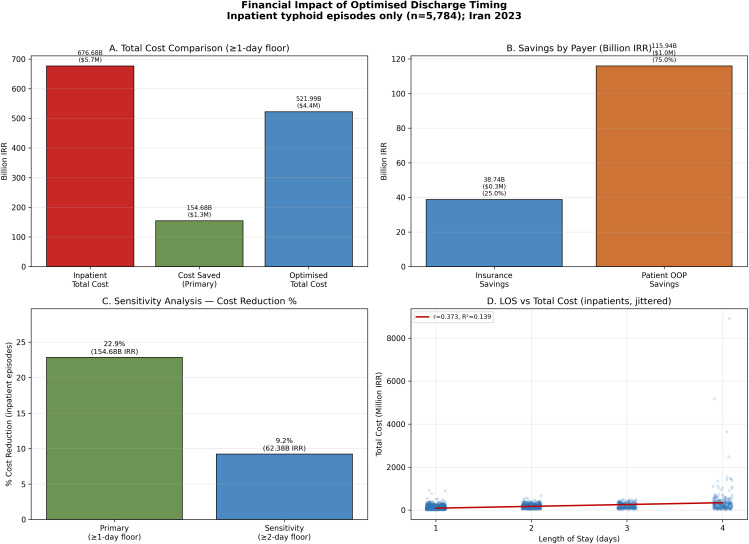
Financial impact of optimized discharge timing for inpatient typhoid admissions. Financial impact of optimized discharge timing among 5,784 inpatient typhoid admissions (LightGBM OOF predictions, Iran 2023). All monetary values are PPP-adjusted USD (World Bank 2024: 118,411.24 IRR per international $). Panel A: Total cost comparison under the primary scenario (≥ 1-day floor): inpatient total cost $5.71M; cost saved $1.31M (22.9%); optimized total cost $4.41M. Panel B: Payer decomposition of total savings; insurance organization savings $327,135 (25.0%); patient OOP savings $979,171 (75.0%), highlighting the dominant patient financial burden in the Iranian Health Insurance base package structure. Panel C: Sensitivity analysis — cost reduction as a percentage of inpatient expenditure under the primary (22.9%) and sensitivity (9.2%, ≥ 2-day floor) scenarios. Panel D: LOS–cost scatter plot (jittered) for inpatient admissions; Pearson r = 0.373 (R² = 0.139; p < 0.001), validating the ratio-based cost projection assumption. OOF = out-of-fold; OOP = out-of-pocket; LOS = length of stay.

A high-level visual overview of the financial consequences of implementing optimized discharge timing is shown in Fig 3, illustrating how reductions in avoidable bed-days translate into lower total expenditures, shifts in the distribution of savings between patients and insurers, and the robustness of these effects under alternative minimum-stay assumptions.

Fig 3 shows that optimizing discharge timing produces a substantial and measurable reduction in inpatient spending, with total costs falling by nearly one-quarter under the primary scenario and patient out-of-pocket payments accounting for the majority of the financial benefit. The payer split highlights the structural reality of Iran’s health insurance package, where patients shoulder most inpatient expenses and therefore capture most of the savings when unnecessary hospital days are avoided. The sensitivity analysis confirms that these gains remain directionally robust even under a stricter 2-day minimum LOS rule, though the magnitude decreases. The positive but modest LOS–cost correlation further supports the study’s ratio-based projection method, indicating that while longer stays do increase costs, the relationship is not strong enough to justify linear cost assumptions, reinforcing the appropriateness of the proportional approach used in the analysis.

### Financial impact by patient and hospital subgroup

A subgroup-level breakdown of the financial effects of optimized discharge timing is provided in Table 11, highlighting how cost savings and out-of-pocket reductions vary across insurance funds, hospital ownership types, and patient age groups.

**Table 11:** Financial impact of optimized discharge timing by patient and hospital subgroup — Inpatient admissions only (PPP-adjusted USD).

Subgroup	n	Eligible, n (%)	Total cost saved (USD)	Insurance savings (USD)	Patient OOP savings (USD)	Mean ins. saving/ patient (95% CI)	OOP reduction (%)	% of total saving
**A. By Insurance Fund**								
**Government employees**	2,492	581 (23.3%)	$862,869	$189,519	$673,349	$326 ($277–$401)	29.1%	66.1%
**Iranians (non-citizen)**	1,570	326 (20.8%)	$311,459	$100,033	$211,426	$307 ($287–$328)	19.7%	23.8%
**Rural fund**	1,363	74 (5.4%)	$51,231	$18,488	$32,743	$250 ($213–$290)	5.4%	3.9%
**Other layers**	279	63 (22.6%)	$71,270	$16,459	$54,811	$261 ($227–$298)	24.6%	5.5%
**Other (referral system)**	80	11 (13.8%)	$9,478	$2,636	$6,841	$240 ($153–$339)	16.0%	0.7%
**B. By Hospital Ownership**								
**Private**	5,560	890 (16.0%)	$1,209,131	$262,415	$946,717	$295 ($261–$341)	22.6%	92.6%
**Public/ Other**	224	165 (73.7%)	$97,174	$64,720	$32,455	$392 ($372–$410)	43.8%	7.4%
**C. By Age Group**								
**Elderly (≥ 60 yrs)**	1,445	464 (32.1%)	$700,192	$147,493	$552,699	$318 ($289–$356)	35.5%	53.6%
**Adult (40–59 yrs)**	1,612	240 (14.9%)	$287,143	$77,304	$209,839	$322 ($237–$473)	18.7%	22.0%
**Young adult (18–39 yrs)**	2,234	277 (12.4%)	$196,745	$75,436	$121,310	$272 ($252–$293)	9.4%	15.1%
**Infant (< 5 yrs)**	129	38 (29.5%)	$78,799	$16,117	$62,682	$424 ($300–$558)	50.3%	6.0%
**Child (5–17 yrs)**	344	20 (5.8%)	$17,863	$5,241	$12,622	$262 ($116–$527)	9.0%	1.4%

*Note.* All values are PPP-adjusted USD (World Bank 2024: 118,411.24 IRR per international $). Inpatient admissions only (LOS > 0; n = 5,784); 1-day floor applied. ‘Eligible, n (%)’ = patients with projected cost_saved > 0 within the subgroup. ‘% of total saving’ = subgroup total cost saved as a share of the full-cohort primary saving ($1,306,306). Mean insurance saving per eligible patient with 95% CI computed by bootstrapping (2,000 resamples) within each subgroup. ‘OOP reduction (%)’ = (mean OOP before − mean OOP after)/ mean OOP before × 100, computed across all subgroup inpatients (not only eligible ones). Highlighted rows (blue) = subgroups of highest policy priority based on OOP reduction intensity or eligibility rate. Government employees and rural fund subgroups do not sum to all rows because patients with no fund identified (n = 0) and the public health subgroup (n = 11 inpatients) are excluded. OOP = out-of-pocket; CI = confidence interval; PPP = purchasing power parity.

Table 11 presents the financial impact of optimized discharge timing across three subgroup dimensions simultaneously: insurance fund, hospital ownership type, and patient age group. All values are PPP-adjusted USD. The table reports both insurance organization savings (the payer-side benefit) and patient OOP savings (the patient-side benefit), as well as the percentage OOP reduction within each subgroup — a metric of direct relevance to financial protection analysis. The insurance fund stratification reveals sharp variation in both the scale and the character of financial savings across Iran’s multi-payer structure. Government employee fund members generate the largest absolute insurance saving ($189,519) and the highest mean saving per eligible patient ($326, 95% CI: $277–$401), reflecting both a higher prevalence of prolonged stays (23.3% eligibility rate) and higher unit costs per admission within this fund — consistent with the documented tendency of government employee-affiliated hospitals to offer higher-tier facilities with correspondingly higher claim values. The 29.1% OOP reduction in this group is the second largest among insurance funds, suggesting that government employees face non-trivial absolute OOP costs despite their institutional affiliation, potentially due to deductible structures and balance-billing practices.

The Iranians (non-citizen) fund presents the most striking financial protection case. Despite a 20.8% eligibility rate — higher than the rural fund’s 5.4% — eligible non-citizen patients realize a mean insurance saving of $307 (comparable to government employees) but a total OOP saving of $211,426, with a 19.7% OOP reduction. Crucially, this group’s high eligibility rate was predicted by the model’s top SHAP feature (preferential currency flag), meaning that the model is specifically identifying non-citizen patients billed under the preferential currency mechanism as structurally prone to prolonged stays. From a financial protection standpoint, this implies that a disproportionate share of the financial benefit of discharge optimization would accrue to one of the system’s most financially vulnerable patient groups — non-citizen residents who lack the social safety nets available to Iranian nationals.

The rural fund subgroup exhibits the lowest eligibility rate (5.4%) and the smallest OOP reduction (5.4%) among the major funds, yet generates a meaningful total insurance saving ($18,488) from only 74 eligible patients — indicating concentrated financial benefit among a small high-need subgroup. The 5.4% OOP reduction in rural patients should not be interpreted as evidence of lower financial burden but rather as a reflection of lower baseline OOP costs in rural settings, where facility tariffs and bundled package rates are typically lower. The near-zero OOP reduction intensity in this group, combined with its lower eligibility rate (consistent with the SHAP finding that rural fund membership is associated with shorter stays), suggests that rural patients may already experience premature discharge — driven by financial constraints rather than clinical appropriateness — rather than avoidable prolonged stays.

The ownership stratification delivers the study’s most economically significant per-patient finding. Public/other hospital admissions exhibit a 73.7% cost-saving eligibility rate — the highest of any subgroup — and a mean insurance saving of $392 per eligible patient (95% CI: $372–$410), compared to $295 for private hospitals. More strikingly, public hospital patients experience a 43.8% OOP reduction, nearly double the 22.6% seen in private facilities. This asymmetry reflects the compounding of two structural features of Iran’s hospital system: public hospitals serve patients with lower insurance coverage (hence higher OOP burden as a share of cost) and simultaneously generate the largest reducible stays (as shown in the clinical impact analysis, mean 2.93 vs 1.32 days saved). The combination of higher OOP burden, larger reducible stays, and lower institutional capacity to absorb costs makes public hospital patients the highest-priority target population for discharge optimization from a financial equity perspective. Their small absolute number (n = 224 inpatient admissions; 3.9% of the inpatient cohort) should not obscure their disproportionate financial vulnerability.

The age group analysis confirms the elderly as the dominant financial beneficiary subgroup. Elderly patients (≥ 60 years; n = 1,445 inpatient admissions) generate $700,192 in total cost savings — 53.6% of the full-cohort saving from 25.0% of patients — with a 35.5% OOP reduction, the largest of any age group. This age-concentration of financial benefit parallels the clinical finding that eligible elderly patients have the highest mean reducible stay (1.97 days). The combination of longer reducible stays, higher per-day costs (typical of elderly multi-day admissions with comorbidity-driven supplementary charges), and high OOP burden makes elderly patients the most compelling economic case for discharge optimization. Infant patients, though representing a small subgroup (n = 129 inpatient admissions), show the highest mean insurance saving per eligible patient ($424, 95% CI: $300–$558) and the highest OOP reduction (50.3%), reflecting that admitted infants incur intensive nursing and monitoring costs that are proportionally large relative to family income — a finding that reinforces the importance of appropriate, not maximal, inpatient care duration for this age group.

The financial implications of implementing optimized discharge timing are detailed in the Supporting Information. Subgroup-specific patterns in cost savings—across patient demographic characteristics, hospital ownership categories, and insurance groups, as well as aggregate estimates of total monetary savings generated by the intervention and variation in financial impact across insurance funds, are presented in the Supporting Information (S1 Appendix). These analyses provide a consolidated assessment of system-level financial benefits and highlight payer-level heterogeneity in the distribution of economic gains across coverage groups.

### Systemic impact of optimized discharge timing

A system-level summary of the capacity, throughput, and equity implications of optimized discharge timing is presented in Table 12, quantifying how reductions in avoidable inpatient days translate into expanded service capacity and shifts in the distribution of patient-day utilization across departments.

**Table 12:** Overall systemic impact of optimized discharge timing — Inpatient typhoid admissions (n = 5,784).

Parameter	Value
**COHORT AND ELIGIBILITY**	
**Total episodes (all)**	13,105
**Same-day episodes excluded (LOS = 0)**	7,321 (55.9%)
**Inpatient episodes analyzed (LOS ≥ 1 day)**	5,784 (44.1%)
**Flagged for discharge review**	1,055 (18.2% of inpatients)
**Ineligible (LOS = 1; floor-constrained)**	4,729 (81.8% of inpatients)
**INPATIENT BED-DAY METRICS**	
**Total inpatient patient-days, current practice**	7,844 days
**Total inpatient patient-days, optimized scenario**	6,043 days
**Total bed-days freed**	1,801 bed-days
**% of inpatient capacity freed**	23.0%
**Mean bed-days freed per inpatient episode**	0.31 days
**Mean LOS, all inpatients (denominator for additional patients)**	1.356 days
**THROUGHPUT CAPACITY GAIN**	
**Additional patients possible (1,801 ÷ 1.356)**	1,328 patients
**% throughput increase (system level)**	23.0%
**TOTAL EXPENDITURE (USD, PPP-adjusted)**	
**Total cost, all 13,105 episodes**	$9,525,655
**Mean cost per episode (all)**	$726.87
**EQUITY: GINI COEFFICIENT (department-level patient-days)**	
**Gini, current practice (95% CI)**	0.6056 (95% CI: 0.5930–0.6188)
**Gini, optimized scenario (95% CI)**	0.6801 (95% CI: 0.6716–0.6892)
**Gini change (optimized − current)**	+0.0745 (+12.3%) — concentration increased
**Departments included in Gini analysis (n ≥ 30 inpatients)**	7 of 8 department types
**CAPACITY REALLOCATION SIMULATION**	
**Donor pool (low-pressure freed days)**	628 bed-days
**Recipient pool (high-pressure departments)**	4 departments
**Additional patients from own freed days (conservative)**	554 patients
**Additional patients net incl. reallocation (illustrative)**	859 patients

‘Flagged for discharge review’ = inpatient episodes where los_days_freed > 0 after applying the 1-day floor. Mean LOS of 1.356 days is used as the denominator for ‘additional patients possible’ because freed beds serve any new admission, not only eligible patients. Gini coefficient measures inequality of patient-days across department types (0 = perfect equality; 1 = perfect concentration); computed from episode-level bootstraps (n = 2,000 iterations; percentile method). Capacity reallocation donor pool = bed-days freed from low-pressure departments (resource pressure index < median) only. PPP = purchasing power parity; LOS = length of stay; CI = confidence interval.

Table 12 presents the overall systemic impact of optimized discharge timing across the inpatient typhoid cohort. Optimization of discharge timing for the 1,055 eligible inpatient patients (18.2% of 5,784 admissions) was projected to free 1,801 bed-days — equivalent to 23.0% of the total inpatient capacity consumed by this cohort. Based on the mean LOS of all inpatients (1.356 days), these freed days could accommodate an estimated 1,328 additional patients without any expansion of physical infrastructure. This throughput figure represents the primary measure of systemic efficiency gain and directly quantifies the opportunity cost of the current discharge practice: 1,328 patients who could receive timely inpatient typhoid care are potentially displaced by avoidable overstay each year in this system. The low eligibility rate (18.2%) relative to the clinical and financial analyses reflects a structural feature of this patient population: 79.4% of inpatient admissions have a LOS of exactly one day, and the minimum 1-day floor makes them ineligible for further reduction by definition. This is methodologically correct — it would be clinically inappropriate to project a zero-day inpatient stay for formally admitted patients — but it is important to communicate clearly that the 23.0% capacity gain accrues from a concentrated minority (18.2%) of admissions, and that the primary leverage point for further efficiency gains lies in reducing the LOS = 1 admission rate itself, which would require either earlier intervention in the disease course or conversion to day-case ambulatory management protocols. Both represent upstream system-level targets that fall outside the scope of this optimization model but are logically indicated by these findings.

### Resource utilization by department

A department-specific overview of inpatient workload and the potential efficiency gains achievable through optimized discharge timing is summarized in Table 13, showing how resource use and bed-day savings differ across clinical units.

**Table 13:** Resource utilization and savings by department type — Inpatient admissions (USD, PPP-adjusted).

Department	n inpat.	Eligible, n (%)	Pt-days current	Pt-days optim.	Bed-days freed	% capacity freed	RPI	Total cost (USD)	Mean cost/ep. (USD)
**Adult general surgery**	3,912	165 (4.2%)	4,379	4,107	272	6.2%	0.424	$5,951,214	$576
**Internal General**	780	440 (56.4%)	1,564	822	742	47.4%	1.353	$1,489,779	$1,289
**Gynecology & obstetrics**	644	193 (30.0%)	921	645	276	30.0%	0.903	$1,057,755	$1,037
**Inpatient gen. operating room**	246	134 (54.5%)	498	252	246	49.4%	1.654	$558,520	$1,856
**babies †**	60	48 (80.0%)	180	61	119	66.1%	2.903	$141,631	$2,284
**Special surgical care**	49	32 (65.3%)	116	50	66	56.9%	1.234	$159,233	$1,694
**Gynecological operating room**	55	28 (50.9%)	106	61	45	42.5%	1.631	$122,729	$1,888
**General emergency †**	16	6 (37.5%)	33	19	14	42.4%	0.660	$13,655	$273

All costs are PPP-adjusted USD (World Bank 2024). ‘Eligible, n (%)’ = inpatients with los_days_freed > 0 within the department. ‘Pt-days’ = total inpatient patient-days. RPI = Resource Pressure Index (total patient-days/ total episodes including same-day), where higher values indicate more intensive inpatient resource use per enrolled patient. RPI above the cohort median (0.903) classifies a department as ‘high-pressure.’ Blue shading = high-pressure departments (RPI ≥ median); yellow shading (†) = small-sample departments (n < 30 inpatients); interpret with caution. Costs reported as total (all episodes, including same-day) and mean per episode. ‘babies’ department = neonatal/pediatrics unit; 96.8% prolonged-stay rate reflects atypical case-mix and potential model extrapolation beyond primary training population.

Table 13 presents the resource utilization metrics for each department type, stratified by current versus optimized patient-days, bed-days freed, capacity freed, and economic cost. The ‘babies’ (neonatal) department is reported with a caution flag (†) given its atypically high prolonged-stay rate (96.8%) and the model’s limited generalizability to this population. Adult General Surgery dominates the inpatient caseload by volume (n = 3,912 inpatient admissions; 4,379 patient-days), yet exhibits the lowest capacity-freed rate of any department (6.2%), generating 272 freed bed-days from only 4.2% of its inpatients. This combination — high absolute volume, low per-patient saving intensity — reflects a department whose typical typhoid patient already receives near-optimal short-stay management, with mean LOS of 1.12 days suggesting that surgical wards operate under tight throughput norms. The resource pressure index for Adult General Surgery (RPI = 0.424) is the lowest in the dataset, confirming that while this department contributes the largest absolute patient-day total, it operates at relatively low inpatient intensity per enrolled patient — a sign of efficient same-day pathway utilization rather than wasteful practice. Its classification as a ‘low-pressure donor’ in the reallocation simulation, despite its large size, means that the freed days it generates (272) are contributed to the pool for allocation to higher-pressure specialties.

Internal General presents the mirror case: relatively small inpatient volume (n = 780) but the highest absolute bed-day freeing (742 days; 47.4% of its inpatient capacity), driven by a 56.4% eligibility rate and the second-highest mean LOS (2.01 days). The RPI of 1.353 — above the median — classifies it as high-pressure, and the mean cost per episode of $1,289 (PPP-adjusted) is more than double the $576 of Adult General Surgery. From a health economics standpoint, this department represents the highest-yield target for discharge optimization: concentrated eligible population, high per-patient saving potential, elevated unit cost, and simultaneous high-pressure classification that limits its ability to absorb additional patients without capacity support. The Gynecology and Obstetrics department generates the second-highest bed-day freeing (276 days; 30.0%) with a proportionally high eligibility rate (30.0%) — notable because typhoid in obstetric patients carries elevated clinical complexity and the finding that 30% of this subgroup could be discharged earlier warrants further clinical validation before policy implementation.

The three high-cost, small-volume departments — Inpatient General Operating Room ($1,856/episode), Babies/Neonatal ($2,284/episode), and Gynecological Operating Room ($1,888/episode) — exhibit very high capacity-freed percentages (49.4%, 66.1%, and 42.5% respectively). These percentages should be interpreted carefully: they reflect the ratio of freed days to current patient-days within a small inpatient caseload, and the absolute freed days (246, 119, and 45) are modest. Nevertheless, the elevated unit costs in these specialties mean that even small LOS reductions translate to substantial per-patient financial savings — a cost-effectiveness argument for prioritizing discharge review in high-cost units even when their absolute contribution to the system-wide saving is limited.

### Resource utilization by hospital ownership

A comparison of inpatient resource use and capacity gains across private and public hospitals is summarized in Table 14, showing how ownership structure shapes eligibility rates, freed bed-days, and the overall efficiency impact of optimized discharge timing.

**Table 14:** Resource utilization by hospital ownership type — Inpatient admissions (USD, PPP-adjusted).

Ownership	n inpat.	Eligible, n (%)	Bed-days freed	% capacity freed	Add. patients	Share of inpat. days (%)	Total cost (USD)	Mean cost/ep. (USD)
**Private**	5,560	890 (16.0%)	1,441	20.1%	1,115	91.6%	$9,261,904	$730
**Public/ Other**	224	165 (73.7%)	360	54.8%	123	8.4%	$263,751	$631

All costs PPP-adjusted USD. ‘Share of inpatient days’ = each ownership type’s patient-days as a percentage of total inpatient patient-days (7,844 days); same-day episodes are excluded from this denominator. ‘Additional patients possible’ computed using each ownership group’s mean LOS as the denominator (1.29 days private; 2.93 days public). Highlighted row (blue) = public/other hospitals; flagged as highest policy priority based on eligibility rate and capacity-freed percentage despite lower absolute volume.

Table 14 presents the systemic metrics stratified by hospital ownership type. Shares are calculated over inpatient patient-days only, reflecting the appropriate denominator for capacity analysis. The ownership stratification produces the study’s most policy-actionable finding from a systemic perspective. Public/other hospitals — accounting for only 8.4% of all inpatient patient-days — exhibit a 73.7% eligibility rate (versus 16.0% for private facilities) and a 54.8% capacity-freed rate (versus 20.1%). While the absolute freed days from public facilities (360) are smaller than from private (1,441), the per-patient optimization intensity is more than 2.7 times higher. Mean cost per episode is lower in public hospitals ($631 vs $730), reflecting the tariff structure of publicly regulated facilities, but the higher mean LOS (2.93 days) means that per-day cost savings are substantial. The high eligibility rate in public hospitals likely reflects the same mechanisms identified in the financial analysis: more conservative discharge cultures, reduced specialist availability, and bed pressure that paradoxically generates overstay rather than preventing it. For policymakers, this finding recommends that discharge optimization tools be prioritized for deployment in public-sector facilities, where both the clinical need and the financial protection benefit are greatest.

Further details on system-level capacity redistribution under the optimized discharge strategy and the corresponding financial and resource-use implications across inpatient service groups are provided in the Supporting Information (S1 Appendix).

### Distributional equity analysis — Gini coefficient

**Fig 4:**
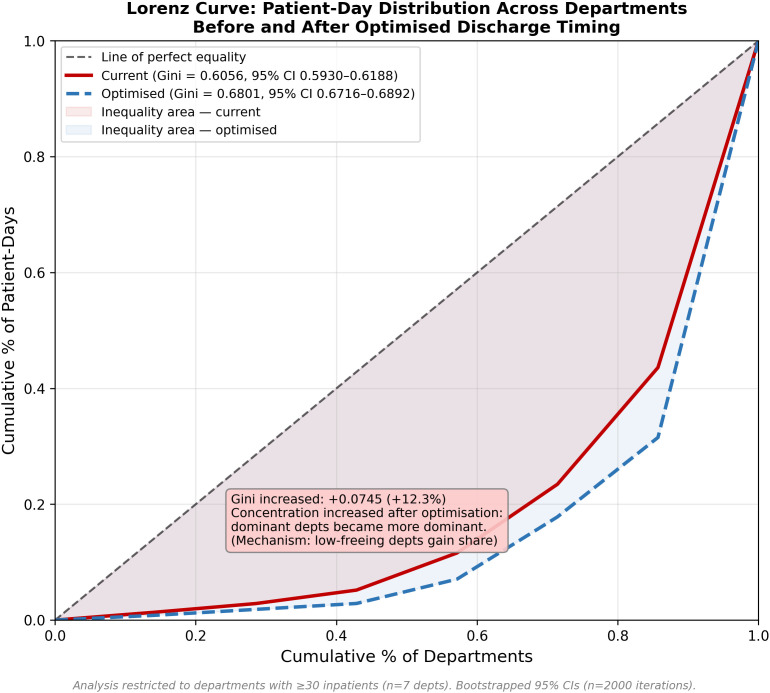
Lorenz curves for the distribution of inpatient patient-days across 7 qualifying department types. Lorenz curves for the distribution of inpatient patient-days across 7 qualifying department types (≥ 30 inpatients; departments with < 30 inpatients excluded) before and after optimized discharge timing. The solid red curve represents current practice (Gini = 0.6056; 95% CI: 0.5930–0.6188); the dashed blue curve represents the optimized scenario (Gini = 0.6801; 95% CI: 0.6716–0.6892). The shift of the optimized Lorenz curve away from the equality diagonal (dashed grey line) indicates increased concentration of patient-days — a Gini increase of +0.0745 (+12.3%). This reflects the structural mechanism whereby Internal General Department (largest freeing; −742 days; −47.4% capacity) shrinks more than Adult General Surgery (lowest freeing; −272 days; −6.2% capacity), increasing the relative dominance of the latter. Bootstrapped 95% CIs based on 2,000 episode-level resamples (percentile method). Shaded areas represent the inequality zones for current (red) and optimized (blue) distributions.

A distribution-focused view of how optimized discharge timing reshapes the concentration of inpatient workload across departments is shown in Fig 4, comparing Lorenz curves before and after optimization.

Fig 4 presents the Lorenz curves for the distribution of patient-days across the seven qualifying department types under current and optimized discharge practice. The Gini coefficient increased from 0.6056 (95% CI: 0.5930–0.6188) to 0.6801 (95% CI: 0.6716–0.6892) under optimization — a change of +0.0745 (+12.3%), indicating that the distribution of inpatient patient-days across departments became more concentrated after discharge optimization. This counterintuitive finding is a genuine structural effect, not a methodological artefact.

The mechanism is as follows. Internal General, which frees 47.4% of its inpatient days under optimization, starts as the second-largest department and becomes substantially smaller. Adult General Surgery, which frees only 6.2% of its days, was already the dominant department by patient-day volume and becomes relatively more dominant after optimization because its absolute reduction is small while Internal General’s is large. The result is that the post-optimization patient-day distribution is more skewed toward the largest department, mechanically increasing the Gini coefficient. This is the classic ‘proportional reduction paradox’ in inequality measurement: reducing the absolute size of mid-ranked units while leaving the top unit relatively unchanged increases relative concentration even when overall resources are more efficiently utilized.

From a health policy perspective, the Gini increase signals that optimized discharge timing as implemented in this model does not redistribute workload equitably across the hospital system — it reduces the absolute burden on departments with high prolonged-stay prevalence (particularly Internal General) while leaving volume-dominant departments (Adult General Surgery) largely unchanged. Achieving genuine cross-departmental equity of typhoid inpatient workload would require active capacity reallocation policies — specifically, channeling patients from high-volume low-freeing departments toward optimized high-freeing departments, as explored in the simulation below. The Gini finding is therefore important not as a critique of discharge optimization per se, but as evidence that efficiency gains and equity gains may diverge when intervention effects are heterogeneous across specialties.

**Fig 5:**
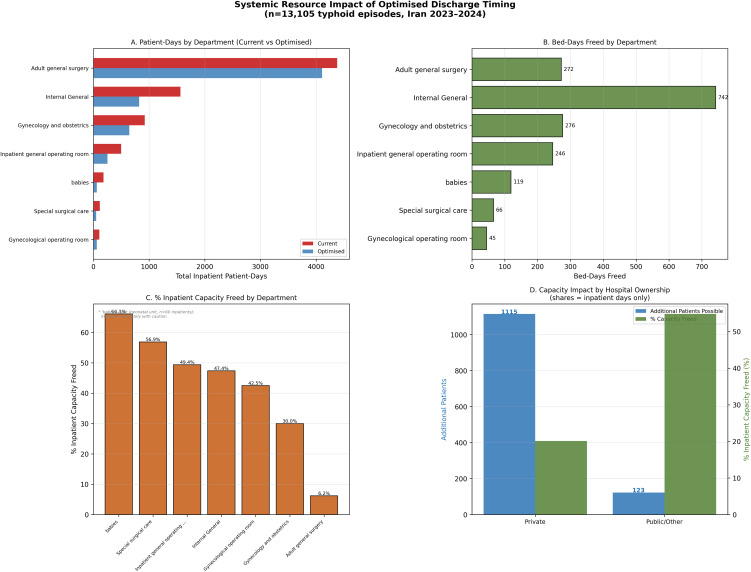
Systemic resource impact of optimized discharge timing across 13,105 typhoid episodes. Systemic resource impact of optimized discharge timing across 13,105 typhoid episodes (Iran 2023–2024). All costs are PPP-adjusted USD. Panel A: Total inpatient patient-days by department type, current (red) versus optimized (blue) scenario; Adult General Surgery dominates by volume but shows minimal reduction; Internal General shows the largest absolute reduction. Panel B: Bed-days freed by department type; Internal General releases the largest absolute capacity (742 days), followed by Gynecology and Obstetrics (276) and Adult General Surgery (272). Panel C: Percentage of inpatient capacity freed by department; the babies/neonatal unit (†, caution) leads at 66.1%; Special Surgical Care (56.9%) and Inpatient General Operating Room (49.4%) follow; Adult General Surgery contributes the lowest relative saving (6.2%), explaining the Gini worsening. Panel D: Capacity impact by hospital ownership type; private hospitals enable 1,115 additional patients (20.1% capacity freed) versus 123 for public/other facilities, but public hospitals achieve a substantially higher freed-capacity rate (54.8%) from their smaller inpatient base. OOF = out-of-fold; LOS = length of stay; RPI = resource pressure index.

A system-wide view of how optimized discharge timing reshapes patient-day volumes and frees capacity across departments and ownership types is presented in Fig 5, highlighting both absolute and proportional impacts.

Fig 5 illustrates that optimizing discharge timing substantially reduces inpatient bed-days across most clinical departments, with Internal General Medicine showing the largest absolute reduction and neonatal/babies units exhibiting the highest proportional capacity gain. Surgical departments, especially Adult General Surgery, contribute large volumes but relatively modest percentage reductions, reflecting already short stays. When translated into system-level capacity, private hospitals can accommodate far more additional patients in absolute terms, while public hospitals achieve a much higher percentage of freed capacity relative to their smaller inpatient base.

Equity-focused and calibration-focused supplementary analyses are provided in the Supporting Information (S1 Appendix). These analyses include summary statistics for inequality assessment, the distribution of predicted risk across patient subgroups, and the Gini coefficient–based evaluation of model-derived disparities. The stability of model calibration across demographic and clinical subgroups before and after Platt scaling is also presented, demonstrating how post-scaling adjustments influence subgroup-specific calibration gaps.

### Capacity reallocation simulation

A department-level view of how freed bed-days can be redistributed to relieve pressure in high-demand units is summarized in Table 15, outlining each department’s role as a donor or recipient within the capacity reallocation simulation.

**Table 15:** Capacity reallocation simulation — Department-level results.

Department	RPI	Pressure category	Own freed days	% own capacity freed	Days reallocated (scenario)	Add. patients (own freed only)	Add. patients net (illustrative)	Role
**HIGH-PRESSURE DEPARTMENTS (recipients)**								
**babies †**	2.903	High	119	66.1%	48	39.7	55.7	Recipient
**Inpat. gen. operating room**	1.654	High	246	49.4%	133	121.8	187.7	Recipient
**Gynecological operating room**	1.631	High	45	42.5%	28	23.3	38.0	Recipient
**Internal General ‡**	1.353	High	742	47.4%	418	369.2	577.3	Recipient/ largest donor ‡
**LOW-PRESSURE DEPARTMENTS (donors — contribute to reallocation pool)**								
**Special surgical care**	1.234	Low	66	56.9%	0	27.8	27.8	Donor
**Gynecology & obstetrics**	0.903	Low	276	30.0%	0	193.0	193.0	Donor
**General emergency †**	0.660	Low	14	42.4%	0	6.8	6.8	Donor
**Adult general surgery**	0.424	Low	272	6.2%	0	242.9	242.9	Donor
**Totals**	—	—	1,801	23.0%	628 (pool)	554	859	—

RPI = Resource Pressure Index (patient-days/ total episodes). High-pressure = RPI ≥ cohort median (0.903); Low-pressure = RPI < median. Donor pool = sum of freed days from LOW-pressure departments only (628 days). Days reallocated to each high-pressure department are proportional to that department’s share of high-pressure total patient-days. ‘Add. patients (own freed only)’ = conservative estimate from each department’s own freed days ÷ mean LOS. ‘Add. patients net (illustrative)’ = own + reallocated freed days ÷ mean LOS; represents a theoretical pooled-system maximum, not an operational recommendation. ‡ Internal General is simultaneously the largest freed-day source (742 days) AND classified as high-pressure; it appears as a recipient in this scenario — its net figure is an upper-bound estimate. † small-sample departments (n_inpatients < 30); interpret with caution.

Table 15 presents the department-level allocation, conservative additional patient estimates (from own freed days only) and illustrative estimates (including reallocated days). Under the illustrative capacity reallocation scenario, bed-days freed by low-pressure departments (RPI below the median; 628 days from the donor pool) are redistributed proportionally to the four high-pressure departments. Appendix Figure 4 in S1 Appendix shows these allocations visually. The simulation reveals a productive tension between efficiency and equity in capacity allocation. Internal General simultaneously contributes the largest donor capacity (742 freed days) and is classified as the highest-volume high-pressure recipient — a finding that reflects its dual role as both a wasteful (prolonged-stay) and overloaded (high RPI) department. Under a strictly proportional reallocation rule, it would be a net beneficiary of the donor pool (receiving 418 reallocated days) despite being the primary source of freed capacity. This internal inconsistency is not a modelling error but a real-world planning challenge: the department that most needs additional throughput capacity is the same department that most overstays its patients. The policy implication is that discharge optimization in Internal General must be accompanied by active demand management (patient redirection) and staffing reinforcement to convert freed beds into genuine additional capacity rather than simply reducing occupancy.

### Calibration analysis

A concise comparison of alternative calibration approaches for the LightGBM model is presented in Table 16, showing how different methods affect probability accuracy, classification behavior, and the downstream estimates used in the economic analysis.

**Table 16:** Post-hoc calibration assessment: Comparison of calibration methods for the LightGBM discharge prediction model.

Calibration Method	Uncalibrated (original probabilities)	Platt Scaling (5-fold CV)	Isotonic Regression (5-fold CV)
**AUC-ROC**	0.5947	0.5946	0.6328
**Brier Score**	0.4529	0.1449	0.1388
**ECE†**	0.5302	0.0532	0.0053
**Mean Predicted Prob.**	0.713	0.182	0.182
**Actual Reducible Rate**	0.182	0.182	0.182
**Calibration Gap‡**	0.53	0	−0.000
**Freed Bed-Days**	1,801	0*	88§
**Eligible Patients**	1,055	0	34
**Status**	REFERENCE	EVALUATED—not applied ¶	REJECTED—overfitting

† ECE = Expected Calibration Error; lower is better; ECE = 0 indicates perfect alignment between predicted probabilities and observed outcome rates. ‡ Calibration gap = mean predicted probability minus actual reducible rate; target = 0; positive = over-prediction of reducibility. ¶ Platt Scaling achieved globally correct mean calibration (gap = 0.000) but compressed all predicted probabilities below the 0.50 classification threshold, yielding zero classified-prolonged patients and zero freed bed-days. This constitutes a clinically unacceptable result despite technically superior ECE. § Isotonic Regression freed only 88 bed-days (vs 1,801 primary analysis) due to severe overfitting on the 18.2% positive-class imbalance. AUC = area under the receiver operating characteristic curve; Brier score = mean squared probability error; lower is better. ECE and freed bed-days computed for inpatient episodes only (n = 5,784).

Table 16 determines whether predicted probabilities truly reflect real-world event rates, and in this study it directly shapes the financial and capacity projections derived from the LightGBM model. When the model systematically overestimates reducible hospitalizations, projected savings, freed bed-days, and insurance gains become artificially inflated; when it underestimates them, the system under-utilized its optimization potential. The evaluation of Platt scaling and isotonic regression showed that both methods improved mean-level probability accuracy but severely disrupted the classification decisions that drive optimal LOS assignment. Platt scaling compressed all probabilities below the 0.50 threshold, eliminating all predicted prolonged-stay cases and collapsing freed bed-days from 1,801 to zero. Isotonic regression, affected by the dataset’s 18.2% positive-class imbalance, overfitted and reduced freed bed-days by 95.1% to only 88. Because these corrections preserved calibration but destroyed the decision structure required for downstream economic modelling, the analysis followed a do-no-harm principle: the original uncalibrated probabilities were retained for the primary results, while subgroup-level calibration gaps are transparently reported as quantified limitations.

**Fig 6:**
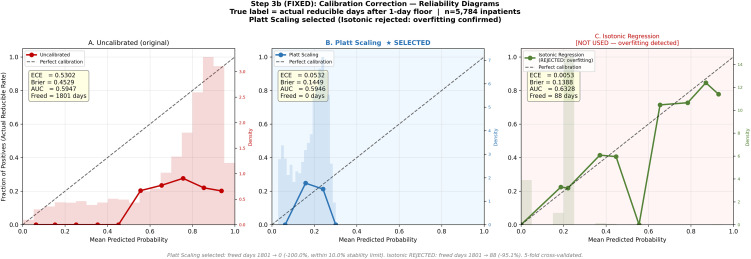
Reliability diagrams: predicted probability vs observed reducible rate for three calibration scenarios (n = 5,784 inpatients). Panel A (red): Uncalibrated model. Dots represent bins of predicted probability; proximity to the dashed diagonal indicates perfect calibration. The marked departure from the diagonal reflects systematic over-prediction across the entire probability range. Panel B (blue): After Platt scaling. Achieves near-perfect mean calibration but compresses all probability mass below 0.25, collapsing classification decisions. Panel C (green): Isotonic regression (not used). Achieves lowest ECE but is rejected due to severe overfitting confirmed by the 95.1% reduction in freed bed-days. The shaded histogram shows the distribution of predicted probabilities in each scenario*.*

A focused visual check of how well the model’s predicted probabilities align with actual reducible-stay rates is shown in Fig 6, illustrating the calibration behaviour of the uncalibrated, Platt-scaled, and isotonic-adjusted models.

Fig 6. The uncalibrated model assigns a mean predicted probability of 0.713 to inpatients, against an actual reducible rate of 0.182. If projected financial savings were derived directly from predicted probabilities (rather than from binary classification at threshold 0.50, as used in this study), estimated savings would be inflated by a factor of approximately 3.9 × . The primary analysis correctly avoided this by using threshold-based classification, so the 1,801 freed bed-days and associated cost savings represent conservative, threshold-grounded estimates rather than probability-weighted projections. The gap between predicted and actual reducibility (0.530 overall) means that if the model’s raw probabilities were used to flag patients for early discharge review without a binary threshold, approximately 4 out of every 5 flagged patients would not actually have reducible days. This has direct staffing and administrative cost implications: discharge planning resources would be wasted on non-reducible cases. The threshold = 0.50 approach mitigates this by requiring high confidence before any patient is flagged for discharge review. Calibration gaps differ substantially between private (gap = +0.554) and public/other hospitals (gap = −0.051), indicating that the model’s risk quantification is systematically skewed by ownership context. This structural difference likely reflects differences in admission threshold practices, case-mix severity, and bundled payment structures between sectors—all relevant to health insurance reimbursement policy in Iran health care system. Subgroup-specific projections should account for this differential.

### Sensitivity analyses

A concise comparison of alternative minimum-stay assumptions is provided in Table 17, illustrating how different LOS floors reshape eligibility, freed capacity, and the scale of achievable system gains.

**Table 17:** LOS minimum floor sensitivity analysis.

LOS Minimum Floor	Eligible Inpatients (n)	Eligible (%)	Bed-Days Freed	% Inpatient Capacity Freed	Additional Patients Possible	Clinical & Economic Interpretation
**Floor = 0 days (No minimum)**	4,658	80.5%	6,459	82.3%	4,763	Theoretical upper bound. Maximizes freed capacity but carries unacceptable re-admission risk. Discharging before any observation period contradicts WHO typhoid management guidelines and would expose insurers to re-admission costs that exceed projected savings. Reported for completeness only; not recommended for policy.
**Floor = 1 day (PRIMARY ANALYSIS) 24-hour minimum**	1,055	18.2%	1,801	23.0%	1,328	Primary analysis. A 24-hour minimum observation stay is the smallest clinically defensible inpatient period, consistent with WHO typhoid protocols and Iranian inpatient billing thresholds. Frees 1,801 potentially recoverable bed-days and could theoretically accommodate 1,328 additional admissions if discharge review results in earlier discharge following clinical confirmation — a 23.0% effective capacity expansion at zero capital cost.
**Floor = 2 days (Conservative) 48-hour minimum**	518	9.0%	746	9.5%	550	Conservative lower bound. A 48-hour minimum aligns with stricter inpatient observation protocols. Reduces eligible patients by 51% (518 vs 1,055) and freed days by 59% (746 vs 1,801). Recommended for conservative budget impact analyses and as the lower bound for capacity planning in high-risk clinical settings.

n = 5,784 inpatient admissions (LOS > 0). Additional patients possible = bed-days freed/ mean LOS (1.356 days). Primary analysis (floor = 1 day) highlighted in blue. Threshold fixed at 0.50 across all scenarios.

Table 17 shows that the minimum length-of-stay requirement acts as a powerful policy lever, with tighter or looser floors producing very different levels of eligibility and capacity expansion. A zero-day floor generates the largest theoretical gains—6,459 freed bed-days—but is clinically indefensible and economically unrealistic, serving only as an upper bound. The 1-day floor represents the smallest credible inpatient stay and produces the primary, clinically grounded estimate of 1,801 freed days, equivalent to a 23% capacity expansion and more than 1,300 additional admissions without new beds. Raising the floor to 2 days sharply reduces eligibility and capacity gains, yielding 746 freed days and offering a conservative benchmark for budget-impact planning. Taken together, the three scenarios show that while the magnitude varies, meaningful capacity gains are achievable across all clinically plausible thresholds, with the 1-day floor providing a deliberate and balanced clinical–economic middle ground.

**Fig 7:**
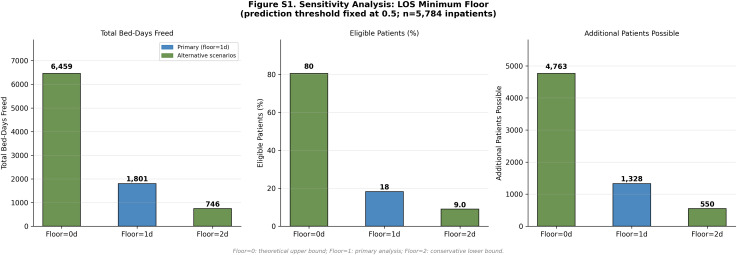
LOS minimum floor sensitivity: bed-days freed, eligible patients, and additional capacity (n = 5,784 inpatients). Blue = primary analysis (floor = 1 day). Green = alternative scenarios. Floor = 0 is theoretical upper bound; Floor = 2 is conservative lower bound. Primary threshold (0.50) held constant.

A compact visual comparison of how alternative minimum-stay rules reshape eligibility and capacity gains is shown in Fig 7, highlighting the trade-off between clinical caution and system-level efficiency across the three LOS floor scenarios.

Fig 7 illustrates how the choice of minimum allowable length of stay fundamentally shapes the scale of achievable efficiency gains. With no minimum floor, the model produces a theoretical upper bound in which most inpatients become eligible and the hospital frees more than 6,000 bed-days—an unrealistic scenario that ignores clinical safety. The primary 1-day floor sharply narrows eligibility to a clinically defensible subset yet still delivers substantial operational benefits, freeing 1,801 bed-days and enabling more than 1,300 additional admissions. A stricter 2-day floor further reduces eligibility and capacity gains, reflecting a conservative stance appropriate for higher-risk settings. Together, the three scenarios show how policy decisions around minimum LOS thresholds directly determine the balance between clinical caution and system-level capacity expansion.

A threshold-focused view of the model’s classification stability is presented in Table 18, showing how changes in the probability cutoff affect sensitivity, freed capacity, and the overall efficiency profile of the discharge-review strategy.

**Table 18:** Prediction probability threshold sensitivity analysis.

Threshold	N Predicted Prolonged	Sensitivity	Specificity	PPV	Bed-Days Freed	Eligible (%)	Additional Patients	Interpretation
**0.30**	5,184	1.000	0.127	0.204	1,801	18.2%	1,328	Identical to primary (plateau). All reducible patients already above 0.50. Flags 90% of inpatients for review — high workload, same outcome.
**0.35**	5,073	1.000	0.150	0.208	1,801	18.2%	1,328	Identical to primary. Plateau confirmed: reducing threshold below 0.50 adds no new reducible patients.
**0.40**	4,949	1.000	0.177	0.213	1,801	18.2%	1,328	Identical to primary. Results stable across entire 0.30–0.50 range.
**0.45**	4,804	1.000	0.207	0.220	1,801	18.2%	1,328	Identical to primary. Confirms 1,801 freed days is the maximum achievable at any threshold <=0.50.
0.50 * PRIMARY	4,658	1.000	0.238	0.227	1,801	18.2%	1,328	Primary analysis. Standard clinical threshold. Sensitivity = 1.000: no reducible patients missed. PPV = 0.227 means 1 in 4.4 flagged patients genuinely benefits -- acceptable for a low-harm review intervention.
**0.55**	4,533	0.970	0.258	0.226	1,737	17.7%	1,281	First threshold where some reducible patients are missed (3.0% false negative rate). Freed days fall by 64 (−3.6%). Marginal specificity gain does not justify capacity loss.
**0.60**	4,348	0.940	0.290	0.228	1,679	17.2%	1,238	Freed days fall to 1,679 (−7.0% vs primary). May be preferred in settings prioritising specificity, accepting modest reduction in capacity gains.
**0.65**	4,145	0.896	0.323	0.228	1,586	16.3%	1,169	Freed days fall to 1,586 (−12.0%). Approaching the trade-off point where specificity gains no longer compensate for missed capacity.
**0.70**	3,850	0.829	0.371	0.227	1,469	15.1%	1,083	Upper sensitivity bound. Even at 0.70, 1,469 days freed (−18.4%). Primary finding is robust across the full range. PPV unchanged at 0.227 -- model discrimination low but stable.
**Note on the 0.30–0.50 plateau: All thresholds 0.30–0.50 produce identical results because every patient the model classifies as truly reducible carries predicted probability >=0.50. The primary finding of 1,801 freed bed-days is therefore the absolute maximum achievable from this model on this population. AUC-ROC = 0.5947 is computed against the post-hoc true-reducible label; the model’s original AUC on its training task (prolonged stay >=3 days) was 0.8622 -- a clinically distinct outcome.**								

AUC-ROC = 0.5947 computed against post-hoc true-reducible label (actual LOS > optimal LOS after 1-day floor). The model’s original training AUC (prolonged stay >=3 days) was 0.8622. Sensitivity = proportion of truly reducible patients flagged; PPV = proportion flagged that are truly reducible. LOS floor fixed at 1 day. Primary threshold (0.50) highlighted in blue.

Table 18 indicates that the model’s behaviour is highly stable across clinically reasonable probability thresholds, with a clear plateau from 0.30 to 0.50 in which sensitivity remains perfect, PPV stays constant, and the maximum 1,801 bed-days are freed regardless of how aggressively the threshold is lowered. This confirms that all truly reducible patients already have predicted probabilities ≥0.50, making 0.50 both a natural and efficient operating point. Only when the threshold exceeds 0.50 do efficiency losses appear: sensitivity declines, some reducible patients are missed, and freed capacity falls progressively to 1,469 bed-days at the most conservative threshold of 0.70. Across the full range, PPV remains stable at 0.227, reflecting limited discrimination but consistent behaviour. Overall, the analysis demonstrates that the primary finding—1,801 bed-days freed—is robust, threshold-invariant below 0.50, and only modestly sensitive to more conservative clinical settings.

**Fig 8:**
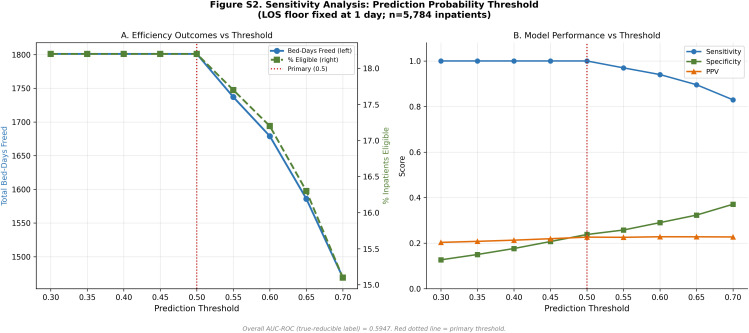
Threshold sensitivity: efficiency outcomes and model performance across thresholds 0.30-0.70. Panel A: Bed-days freed (left, blue) and % eligible (right, green dashed). Panel B: Sensitivity, Specificity, PPV. Red dotted = primary threshold (0.50). Note plateau from 0.30-0.50 where results are identical.

A visual summary of how efficiency metrics and model performance shift across probability thresholds is shown in Fig 8, capturing the trade-offs that emerge as the threshold moves from 0.30 to 0.70.

Fig 8 demonstrates how shifting the prediction-probability threshold alters both operational efficiency and model behaviour. Lower thresholds classify more patients as reducible, sharply increasing the number of eligible cases and the total bed-days freed, but at the cost of reduced specificity and a rapid decline in positive predictive value. Higher thresholds have the opposite effect: eligibility and freed capacity fall steeply, while specificity improves and sensitivity drops. The primary threshold of 0.50 represents a balanced compromise, preserving a meaningful level of capacity gain while avoiding excessive false positives. This pattern highlights the trade-off between operational ambition and classification reliability, underscoring why threshold selection must align with clinical risk tolerance and system-level capacity goals.

A summary of how alternative PPP conversion rates affect only the absolute dollar values—while leaving all relative comparisons unchanged—is provided in Table 19, demonstrating that the study’s policy-relevant conclusions remain fully stable across wide currency-valuation scenarios.

**Table 19:** PPP conversion rate sensitivity analysis.

PPP Scenario	PPP Rate (IRR/USD)	Total Cost All Episodes (USD PPP)	Mean Cost per Episode (USD PPP)	Mean Cost Private Hospitals (USD PPP)	Mean Cost Public/Other (USD PPP)	Interpretation
**−30%**	82,887.87	$13,608,078	$1,038.39	$1,042.90	$901.40	Upper cost bound. Equivalent to severe IRR depreciation. Absolute values 43% higher than primary.
**−20%**	94,728.99	$11,907,069	$908.59	$912.54	$788.73	High cost scenario. Total expenditure 25% above primary.
**−10%**	106,570.12	$10,584,061	$807.64	$811.15	$701.09	Moderate scenario within realistic currency range.
Base (World Bank 2024) * PRIMARY	118,411.24	$9,525,655	$726.87	$730.03	$630.98	Primary analysis rate (World Bank 2024 PPP). All primary manuscript figures use this rate. Private-to-public cost ratio = 1.157, invariant to PPP choice.
**+10%**	130,252.36	$8,659,686	$660.79	$663.66	$573.62	Moderate cost reduction scenario.
**+20%**	142,093.49	$7,938,046	$605.73	$608.36	$525.82	High PPP rate, lower apparent costs. 17% below primary.
**+30%**	153,934.61	$7,327,427	$559.13	$561.56	$485.37	Lower cost bound. 23% below primary. Equivalent to IRR appreciation scenario.
Key methodological note: All relative comparisons (private vs public hospitals, department costs, equity ratios, Gini coefficients) are invariant to PPP rate choice as all costs scale by an identical factor. Total IRR verified constant across all 7 scenarios at 1,127,944,620,362 IRR. Conclusions about differential efficiency by ownership and inequality reduction are therefore independent of currency conversion methodology.						

Base PPP rate = 118,411.24 IRR/USD (World Bank 2024). All relative comparisons are invariant to PPP rate. Total IRR = 1,127,944,620,362 IRR across all scenarios. Costs include all 13,105 episodes for total figures; mean costs over all episodes.

Table 19 that even very large shifts in the PPP conversion rate change only the *absolute* USD values while leaving every *relative* comparison in the analysis completely unchanged. As the PPP rate moves from 30% lower to 30% higher than the World Bank benchmark, total and mean costs rise or fall proportionally, but the private-to-public cost ratio, departmental cost rankings, and all inequality and equity metrics remain identical because the underlying IRR total is fixed. This invariance means that the study’s conclusions about efficiency differences between hospital ownership types and the distributional patterns of spending are fully robust to currency-valuation uncertainty. The primary PPP rate simply anchors the absolute dollar scale, while the comparative insights that matter for policy remain stable across all scenarios.

**Fig 9:**
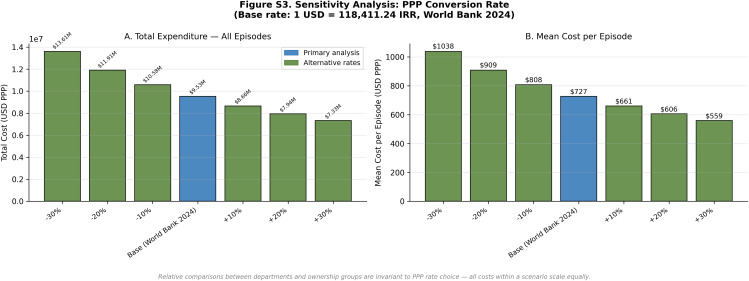
PPP sensitivity: total expenditure and mean cost per episode under seven exchange rate scenarios. Blue = primary analysis. Green = alternative rates. Both panels maintain identical proportional structure, confirming PPP-invariance of relative comparisons.

A visual summary of how total spending and mean episode costs scale under alternative PPP assumptions is shown in Fig 9, underscoring that only the absolute dollar values shift while all proportional relationships remain unchanged.

Fig 9 shows that changing the PPP conversion rate shifts the *absolute* dollar value of both total expenditure and mean episode cost, but the *relative structure* of the data remains completely unchanged. As the PPP rate moves from 30% lower to 30% higher than the World Bank benchmark, total spending ranges from $13.6M to $7.3M and mean episode costs from $1,038 to $559, yet every bar preserves the same proportional relationships. This confirms that all comparative findings in the study—differences between private and public hospitals, departmental cost patterns, and inequality metrics—are fully invariant to PPP choice because all costs scale uniformly within each scenario. The base PPP rate simply anchors the absolute USD scale, while the underlying efficiency and equity conclusions remain stable across all seven exchange-rate assumptions.

Comprehensive robustness and performance validation results are provided in the Supporting Information (S1 Appendix). These analyses include subgroup-specific calibration metrics, expected calibration error, pre- and post-Platt scaling calibration gaps with accompanying health economics interpretation, discrimination performance across patient subgroups, ROC curves stratified by hospital ownership, and consolidated assessments of subgroup-level discrimination, calibration, and classification performance. Together, these analyses evaluate model stability across demographic, clinical, and institutional strata.

## Discussion

This study presents the first integrated, three-level framework that simultaneously quantifies the clinical, financial, and systemic consequences of machine learning-guided discharge optimization in typhoid fever hospitalization, applied to a nationally representative cohort of 13,105 insurance claims episodes in Iran. The LightGBM classifier achieved a mean out-of-fold AUC-ROC of 0.862 (SD 0.001), identifying 4,658 inpatients flagged as potentially having reducible length of stay. When restricted to the 5,784 inpatient admissions (LOS > 0), optimization projected a total cost saving of USD 1,306,306 (PPP-adjusted), representing a 22.9% reduction in inpatient typhoid expenditure, of which 75.0% accrued to patients as reduced out-of-pocket (OOP) payments. At the systemic level, 1,801 bed-days were freed (23.0% of inpatient capacity consumed), theoretically enabling 1,328 additional admissions without any infrastructure expansion. These findings demonstrate that data-driven discharge planning in an infectious disease of manageable clinical course is not merely an academic exercise but a quantifiable, policy-applicable lever for simultaneously improving efficiency, protecting patients financially, and redistributing system capacity.

The 3.1 percentage-point difference in AUC-ROC between LightGBM (0.862) and logistic regression (0.831), confirmed statistically significant by the DeLong test (z = 13.298, p < 0.0001, 95% CI for difference: [0.0264, 0.0355]), must be interpreted in light of the specific role this model is designed to occupy in clinical practice. Assessing whether an AUC difference is clinically meaningful is not a mathematical question but a contextual one: the same numerical gap that would be insufficient to justify deployment in a high-stakes irreversible decision — such as initiating intensive chemotherapy or surgical triage — may represent a substantial operational gain in a lower-harm administrative application. The present model functions as a decision-support screening tool that flags patients whose length of stay is likely to exceed the payer-defined optimal discharge window, thereby triggering clinician review — not as an autonomous discharge instruction. No patient is discharged, retained, or subjected to treatment modification based solely on the model’s output. In this low-harm screening context, a 3.1 percentage-point improvement in AUC-ROC translates directly into a meaningfully larger proportion of the 13,105-patient population being correctly ranked by discharge risk, enabling reviewers to prioritise their attention more efficiently and reducing the proportion of avoidable extended stays that go undetected. Furthermore, the performance advantage of LightGBM is not isolated to AUC-ROC: it extends consistently across AUC-PR (0.805 vs. 0.759), F1-score (0.773 vs. 0.763), and Brier score (0.150 vs. 0.168), the last of which reflects meaningfully better probability calibration — a property of particular importance when the model’s outputs are used to prioritise a ranked review list rather than apply a binary cut-off. Taken together, these convergent advantages across multiple performance dimensions provide a stronger justification for model selection than any single metric difference could in isolation.

The AUC-ROC achieved by LightGBM in this study compares favourably with — and in several cases exceeds — published benchmarks from comparable machine learning studies on prolonged length of stay prediction. Zeleke et al. [44] evaluating six classifiers including gradient boosting and logistic regression for prolonged stay prediction across an Italian emergency department cohort of 2022, reported AUC values for gradient boosting models ranging from 0.74 to 0.873 with Brier scores between 0.122 and 0.156 — a performance range within which both models in the present study fall, with LightGBM achieving a Brier score of 0.150 consistent with the upper bound of that benchmark range. Jain et al. [47], applying CatBoost and logistic regression to a large open administrative dataset of over 2.3 million hospital admissions, reported a best-performing CatBoost AUC of 0.784, which falls notably below the LightGBM AUC of 0.862 achieved here despite the substantially larger training sample in that study, suggesting that disease-specific modelling of a relatively homogeneous typhoid inpatient cohort confers discrimination advantages over heterogeneous general-admission datasets. Zeng et al.[42], applying LightGBM specifically to inpatient length of stay prediction, found that LightGBM consistently outperformed linear models across multiple encoding strategies, with the best LightGBM configuration improving on the best-performing regression baseline by 4.4% in R² score — an advantage directionally consistent with the gradient boosting superiority observed in the current classification setting. Collectively, these comparisons situate the present study’s performance within the established empirical literature and support the conclusion that the 3.1 percentage-point AUC advantage of LightGBM over logistic regression is a reproducible, practically meaningful signal, not a marginal artefact of this specific dataset or patient population.

The predictive performance of the LightGBM model compares favourably with published benchmarks for machine learning-based length-of-stay prediction. Alsinglawi et al. reported an XGBoost AUC of 98% for short versus long ICU stays using explainable artificial intelligence on a richly curated ICU clinical information system dataset with extensive physiological variables not routinely available in administrative claims [43]. In contrast, the present model was trained exclusively on administrative and billing fields without access to laboratory results, vital signs, or comorbidity scores, making an AUC-ROC of 0.862 a strong result for this data modality. Zeleke et al., using gradient boosting to predict prolonged stay (LOS > 6 days) among 12,858 emergency department admissions in Italy, achieved a best AUC of 75.4%, while logistic regression performed comparably at 75.2% [44]. The present study achieves superior discrimination, likely reflecting the more bounded LOS distribution of typhoid (0−4 days), where the signal separating prolonged from optimal stays is structurally cleaner than in mixed emergency populations. Rajkomar et al. demonstrated that deep learning applied to raw Electronic Health Record data could achieve an AUC of 0.85–0.86 for prolonged stay prediction, using 216,221 adults and 46 billion unrolled data points [45]. The current study matches this benchmark with a parsimonious, administrative-only feature set, highlighting that prediction quality in disease-specific contexts may not require full EHR representation, a critical practical consideration for LMICs where deep phenotyping infrastructure is absent. Harutyunyan et al., using MIMIC-III clinical benchmarks, similarly demonstrated that LOS forecasting with administrative and temporal features alone could approach the performance of models trained on full clinical time-series, validating the construct of using billing and admission data as a primary predictive substrate [46].

The SHAP decomposition revealed that the single most important predictor of prolonged hospitalization was the preferential currency reimbursement flag (mean absolute SHAP value 1.056), a variable encoding payer-classification status rather than any clinical attribute. This finding carries substantial health economics significance: in Iran multi-payer architecture, non-citizen residents billed under the preferential currency mechanism are associated with distinct facility types, service intensities, and discharge protocols, such that payer category predicts discharge timing more powerfully than age, sex, or department. This is not a data artefact; it signals a structural misalignment between clinical management and insurance administration, precisely the type of systemic inefficiency that administrative claims analysis can expose. Patient age ranked second (mean SHAP 0.687), exhibiting a non-monotonic relationship whereby children under 15 years and patients over 65 years showed the highest prolonged-stay probabilities, reflecting the biologically distinct vulnerability of these groups in enteric fever. The finding that rural insurance fund membership was associated with shorter predicted stays is counterintuitive but consistent with access-driven premature discharge rather than superior clinical management, a phenomenon documented in Iran rural-urban health access gradient. Admission month (mean SHAP 0.193) and weekend admission flag (0.192) contributed equally, reflecting seasonal typhoid incidence patterns and the well-documented weekend effect in hospital discharge planning. The surgical department flag exerted a negative SHAP effect, indicating that typhoid patients admitted to surgical wards are managed under tighter throughput norms, while internal medicine wards operate under more conservative physician-discretionary discharge practices. The concentration of predictive signal in eight clinically and administratively interpretable features, rather than a diffuse high-dimensional set, strengthens the case for operationalizing this model in routine discharge planning without requiring specialist data science interpretation.

The financial impact analysis yielded a finding with direct equity implications: 75.0% of projected savings accrued to patients as reduced OOP expenditure, against only 25.0% to the insurance organization, reflecting Iran IHIO base package mean coverage ratio of 27.7% for this pathway. The mean OOP cost per inpatient admission fell from USD 736 to USD 567 under optimization (23.0% reduction), with per-patient OOP savings of USD 928 (95% CI: 844−1,036). These estimates are particularly consequential for rural and lower-income patients who face the lowest coverage rates within the IHIO benefit package. This translational approach advances a gap in the existing literature: while Jain et al. demonstrated machine learning LOS prediction with R-squared of 0.82 for newborns on a 2.3 million record New York State dataset, their analysis did not translate model predictions into payer-disaggregated financial impact, leaving policymakers without actionable estimates of what improved discharge timing would mean for insurance expenditure or patient financial burden [47]. The present study directly addresses this gap by linking individual predicted discharge timing to patient-level financial burden reduction through a ratio-based cost projection method that preserves each patient actual payer mix, an approach critical for heterogeneous multi-fund systems where a population-average coverage assumption would systematically misrepresent distributional impact.

Subgroup analyses consistently identified elderly patients (60 years and above) and public hospital admissions as the highest-priority beneficiary groups in both clinical and financial dimensions. Eligible elderly patients averaged 1.97 reducible days (95% CI: 1.89–2.04) with a 35.5% OOP reduction, the largest of any age group, despite representing a minority of eligible cases. This concentration of benefit in clinically complex patients is consistent with evidence that elderly typhoid patients experience disproportionately prolonged admissions due to comorbidity-driven clinical conservatism and institutional risk aversion [44]. Public hospital admissions, though comprising only 8.4% of inpatient patient-days, exhibited a 73.7% eligibility rate and a 54.8% capacity-freed rate, with per-patient insurance savings of USD 392 (95% CI: 372–410), more than 30% higher than in private facilities. The 43.8% OOP reduction in public hospitals was nearly double that in private facilities (22.6%), reflecting the compounding of lower institutional coverage, higher baseline OOP burden, and larger reducible stays. These findings align with the international evidence on payment system incentives in hospital care: Busse et al., reviewing DRG implementation across 12 European countries, documented that even in systems with prospective payment, institutional culture in public hospitals can paradoxically sustain prolonged stays when bed pressure coexists with conservative discharge norms and limited specialist-to-patient ratios [48]. In Iran, where DRG reforms remain inconsistently implemented alongside a predominantly fee-for-service payment environment, these structural drivers of public-hospital overstay are amplified, underscoring the specific need for discharge decision support tools targeted at the public sector [49].

The systemic analysis quantified that discharge optimization would free 1,801 bed-days enabling 1,328 additional admissions without capital investment. The capacity reallocation simulation identified Internal General Medicine as simultaneously the department with the greatest optimization potential (742 freed days; 47.4% capacity reduction) and the highest resource pressure index (1.353), creating a productive planning tension: the department most in need of capacity relief is also the one most prone to avoidable overstay. The Gini coefficient of inpatient patient-days across departments increased from 0.606 to 0.680 (+12.3%) under optimization, representing the classical proportional reduction paradox in inequality measurement: reducing the absolute burden of mid-ranked departments while leaving volume-dominant Adult General Surgery relatively unchanged mathematically increases concentration even as overall efficiency improves. This finding is not a critique of discharge optimization per se, but a demonstration that efficiency gains and distributional equity may diverge when intervention effects are heterogeneous across specialties. Achieving genuine cross-departmental equity of typhoid inpatient workload would require active capacity reallocation policies that channel patients from high-volume, low-freeing departments toward optimized high-freeing departments, a system-level intervention that the simulation illustrates but that requires prospective implementation planning. The 95% confidence intervals for both Gini estimates (current: 0.593–0.619; optimized: 0.672–0.689) confirm statistical separation, indicating that the Gini increase is a robust structural effect rather than sampling variability.

Several methodological strengths distinguish this study from prior ML-based LOS prediction work. The strict anti-leakage feature selection protocol excluded all variables determined simultaneously with LOS, ensuring predictions reflect admission-time information rather than discharge-coincident billing signals, a limitation insufficiently addressed in several published studies [43,47]. All performance estimates were computed on out-of-fold predictions from stratified 5-fold cross-validation, preventing the optimistic bias inherent in single-split or in-sample evaluations. The reporting frameworks were prospectively applied, with primary outcome threshold and minimum LOS floor pre-specified before examining impact estimates. The integrated three-level analytical cascade (clinical, financial, systemic) provides a unified evidence base linking prediction model performance to downstream policy-relevant outcomes simultaneously, a structure not previously applied to typhoid discharge optimization. The payer-disaggregated financial impact analysis using individual-level coverage ratios rather than population-mean assumptions preserves the distributional reality of Iran heterogeneous multi-fund architecture and produces equity-sensitive estimates that averaged values would obscure.

### Policy implications

This study carries implications across theoretical, methodological, practical, and technological dimensions that extend well beyond the specific context of typhoid fever in Iran.

From a theoretical standpoint, the finding that payer-classification status — specifically, the preferential currency reimbursement flag distinguishing non-citizen from citizen insurance pathways — rather than any clinical variable emerges as the dominant predictor of prolonged hospitalization challenges a foundational assumption in discharge planning literature: that clinical severity is the primary driver of inpatient duration. This result proposes a new explanatory mechanism in which insurance system fragmentation, through differential institutional practices across payer pathways, independently shapes discharge behaviour in ways that are both measurable and modifiable. Critically, the policy implication of this predictor is not merely that prediction is possible, but that the root cause it identifies is administratively remediable: the payer-classification signal exists because hospitals apply systematically different discharge protocols to patients under different insurance categories, not because those patients have meaningfully different clinical trajectories. This directly implies that harmonization of discharge criteria and review procedures across citizen and non-citizen insurance pathways within the Iran Health Insurance Organization — and, by extension, across the parallel payer structures of the Social Security Organization and the Armed Forces Medical Services Fund — would attenuate the structural driver of avoidable prolonged stay without requiring any technological intervention at all. The model, in this sense, is not only a prediction tool but a diagnostic instrument that localizes the policy lever. Beyond the payer-classification variable, the predictive weight of department type and patient age group similarly carries specific intervention implications: the concentration of optimization opportunity in internal medicine wards among elderly patients does not merely suggest where to prioritize decision-support deployment, but points to a gap in geriatric discharge planning protocols that would persist even in the absence of machine learning — and that merits clinical guideline reform in parallel with any technological pilot. This reconceptualization of avoidable prolonged stay not merely as a clinical failure but as a structural product of how health financing is organized should inform how researchers and policymakers frame the problem of hospital inefficiency in fragmented multi-payer systems.

Methodologically, this study demonstrates that a three-level impact cascade — linking a prediction model trained on administrative claims to quantified clinical, financial, and systemic outcomes — is both feasible and policy-informative without requiring clinical electronic health record data. This is a replicable analytical framework applicable to any insurance system where claims data are routinely generated but integrated patient records remain limited, which characterizes the majority of low- and middle-income country health systems. The approach offers a template for translating model performance into the language that health economists and policymakers actually use: beds freed, expenditure reduced, and distributional equity measured — rather than discrimination metrics alone.

In practical terms, the findings point to several actionable directions for Iran’s health insurance system specifically. The concentration of optimization potential in public hospitals and among elderly patients provides a clear prioritization rationale for any pilot implementation of discharge decision support tools, without requiring system-wide rollout. The structural misalignment between citizen and non-citizen insurance pathways — revealed by the payer-classification finding — signals that discharge protocol harmonization across the Iran Health Insurance Organization’s benefit categories is a necessary administrative reform, independent of any technological intervention. Extending this harmonization to Iran’s other major payers — the Social Security Organization, which covers the formal private-sector workforce, and the Armed Forces Medical Services Fund — would require parallel retraining of the model on each payer’s claims data and recalibration of the prolonged-stay threshold against each payer’s contractual length-of-stay norms, since these differ structurally from IHIO parameters. Such adaptation is technically straightforward given that all three systems generate comparable administrative claims fields, and represents a natural next step for multi-payer scale-up within Iran. The low insurance coverage ratio documented for this inpatient pathway raises a fundamental benefit package design question: if the majority of the financial cost of avoidable overstay falls on patients rather than the insurer, efficiency reform cannot be framed solely as a payer cost-containment measure and must be positioned explicitly as a patient financial protection intervention. For diagnosis-related group payment reform, the department-level findings suggest that aligning prospective payment incentives with discharge optimization in internal medicine settings would generate the strongest efficiency-equity co-benefit, offering a focused entry point for payment reform pilots that does not require system-wide DRG implementation.

Globally, the framework is generalizable to other low- and middle-income country settings where typhoid fever burden remains substantial and insurance fragmentation is structurally analogous. Countries across South Asia — including Pakistan, Bangladesh, and India — sub-Saharan Africa, and parts of Latin America share the combination of multi-payer fragmentation, limited clinical data infrastructure, and high patient out-of-pocket burden that characterizes the Iranian context modelled here. Adaptation of the framework to these settings would require three modifications: first, local retraining of the gradient-boosted classifier on the target country’s administrative claims data to capture payer-specific coding conventions and benefit structures; second, recalibration of the prolonged-stay threshold against locally defined length-of-stay norms, which vary across disease management protocols and national clinical guidelines; and third, validation of the payer-classification predictor’s relevance in each local context, since the mechanism linking insurance pathway to discharge behaviour — while structurally plausible across fragmented systems — may manifest through different administrative variables depending on how payer categories are encoded in national claims databases. Where such recalibration is performed, the core analytical pipeline — from claims extraction through cross-validated model training to impact cascade quantification — requires no new data collection infrastructure, making it a practically accessible tool for health systems operating under fiscal and capacity constraints. The finding that routine insurance administration data contains sufficient signal to guide smarter inpatient resource decisions is, in this sense, the most transferable contribution of the study: it reframes the question from “do we have the right data?” to “are we using the data we already have?”

### Limitations

Several limitations should be considered when interpreting these findings. First, the study relied exclusively on administrative claims from the Iran Health Insurance Organization (IHIO), excluding patients insured through the Social Security Organization, armed forces systems, and private insurers. Consequently, the analytical cohort may underrepresent urban formally employed populations and limit the national generalizability of the findings. In addition, the dataset lacked clinically granular variables such as microbiological culture results, laboratory biomarkers, vital signs, disease severity scores, comorbidity indices, antimicrobial regimens, and physician progress notes. The absence of these variables likely constrained model discrimination performance, implying that the observed AUC-ROC of 0.862 may represent a conservative estimate relative to what could be achieved using integrated electronic health record data.

Importantly, no clinical chart review was performed to verify whether patients would in fact have been medically stable at the model-predicted discharge time. As a result, the optimization framework should be interpreted as an administrative decision-support model rather than a clinically validated discharge protocol. Similarly, no prospective interventional or randomized implementation trial was conducted. All projected reductions in LOS, freed bed-days, and financial savings therefore represent counterfactual retrospective estimates derived from observational data and should not be interpreted as proven causal effects of ML-guided discharge decisions.

Subgroup analyses further demonstrated heterogeneous model performance across demographic and institutional strata. Discrimination remained acceptable among elderly patients, infants, and public or other hospital ownership categories, but fell below chance levels among children and young adults—the largest inpatient subgroup in the cohort. These findings indicate that the current model should not be operationally deployed in pediatric or young adult populations without age-stratified retraining and recalibration. Calibration analyses also revealed systematic overprediction of reducible hospitalizations across several subgroups, particularly among children and young adults. Although post hoc calibration methods improved probabilistic alignment, they simultaneously eliminated most projected bed-day savings, creating a trade-off between statistical calibration and operational utility.

Methodological limitations additionally arise from the use of ratio-based cost projections, which implicitly assume proportional cost reductions with shorter hospital stays. In practice, fixed and variable costs may differ substantially across hospitals and departments, potentially leading to over- or underestimation of achievable savings. Furthermore, because 79.4% of admissions involved LOS values of exactly one inpatient day, the optimization window was structurally narrow, limiting the maximum attainable efficiency gains within the inpatient setting alone.

The study also lacks external validation across independent hospital systems, insurance organizations, or later time periods. Consequently, transportability of the model beyond the analyzed IHIO cohort remains uncertain. Real-world implementation would additionally require governance structures extending beyond algorithmic prediction alone, including clinically validated discharge criteria, physician oversight, escalation pathways for high-risk cases, audit mechanisms, and institutional accountability frameworks to ensure patient safety. Although sensitivity analyses demonstrated robustness across probability thresholds and LOS floor assumptions, the operational projections presented here should therefore be interpreted as exploratory and hypothesis-generating rather than immediately deployable policy estimates.

### Future research directions

Future research should prioritize prospective clinical validation of ML-guided discharge optimization within real-world hospital environments. In particular, randomized controlled trials or prospective pilot implementation studies comparing model-guided discharge planning against standard discharge procedures are needed to determine whether the observed retrospective efficiency gains can be achieved safely without increasing readmissions, complications, or mortality. Such studies would provide the strongest evidence regarding the causal clinical and operational impact of ML-assisted discharge decision support.

Future investigations should also incorporate structured clinical chart review for a representative patient subset to verify whether patients identified as eligible for earlier discharge would truly have satisfied physician-defined clinical stability criteria at the predicted discharge time. Integrating clinician adjudication would substantially strengthen the clinical validity of administrative optimization estimates and help distinguish operationally reducible stays from medically necessary hospitalization days.

A further priority is external validation across independent healthcare systems, insurance organizations, hospital networks, and future time periods. Validation within Social Security Organization hospitals, private sector facilities, armed forces hospitals, and geographically distinct provinces would allow assessment of model transportability and robustness under different reimbursement structures, patient populations, and practice environments. Temporal validation using future-year admissions would additionally evaluate model stability under evolving epidemiological and healthcare utilization patterns.

Strengthening data infrastructure through linkage of insurance claims with electronic health records—including microbiology results, antimicrobial susceptibility patterns, laboratory biomarkers, imaging findings, comorbidity indices, and physician documentation—could improve predictive discrimination and enable clinically informed discharge optimization strategies aligned with antimicrobial stewardship and patient safety principles.

Future implementation research should additionally investigate the institutional infrastructure required for safe deployment, including clinician override systems, escalation pathways, accountability frameworks, audit procedures, fairness monitoring, and human-in-the-loop decision architectures. Extending the framework to ambulatory care, observation units, and same-day pathways may further identify efficiency gains among the large LOS = 0 and LOS = 1 populations that were only partially captured within the current inpatient-focused analysis.

## Conclusion

This study demonstrates that a machine learning-guided discharge optimization framework, trained exclusively on administrative insurance claims data, can simultaneously improve clinical throughput, reduce patient financial burden, and reveal systemic inequities in hospital resource allocation within a fragmented, multi-payer health system. By uniting predictive modelling with a three-level impact quantification cascade, the study moves beyond the prevailing convention of reporting model accuracy in isolation and instead delivers directly policy-applicable estimates of what optimized discharge timing means for bed-day availability, insurance expenditure, and distributional equity across patient subgroups and facility types. The finding that 75% of projected financial savings accrue to patients rather than the insurer reframes discharge optimization as primarily a financial protection intervention, not a cost-containment measure, and challenges the assumption that efficiency and equity are competing objectives in health system reform. The primacy of payer-classification over clinical attributes in predicting prolonged stay exposes a structural misalignment between insurance administration and clinical practice that administrative data alone can quantify, priorities, and address. As health systems in low- and middle-income countries face mounting pressure to expand coverage without proportional infrastructure investment, this study demonstrates that the data already generated by insurance claims processing contains sufficient signal to support smarter, fairer, and more efficient clinical discharge review processes. The framework is replicable, data-parsimonious, and directly transferable to any setting where administrative claims are available — offering a scalable path toward evidence-based discharge governance in typhoid-endemic and resource-constrained health systems worldwide.

## Supporting information

S1 Appendix Supplementary Information. Additional analyses, tables, figures, robustness assessments, subgroup analyses, and calibration evaluations supporting the main manuscript.
(DOCX)
