# Supplementary material for: Machine learning–optimized discharge timing in typhoid care: Implications for clinical outcomes, cost efficiency, and health system performance
Source: PLoS One. 2026 Jul 23;21(7):e0354148. doi: 10.1371/journal.pone.0354148 (PMC13395383; doi:10.1371/journal.pone.0354148)
Supplement: S1 Appendix — Additional analyses, tables, figures, robustness assessments, subgroup analyses, and calibration evaluations supporting the main manuscript. (DOCX) [file pone.0354148.s001.docx]

**Supporting information**

The Supporting information consolidates all supplementary analyses that deepen, validate, and contextualize the study’s main findings. It includes the full correlation matrix and variance‑inflation diagnostics for the administrative features, SHAP dependence plots for model interpretability, and detailed clinical‑impact tables stratified by month, patient subgroup, hospital ownership, and predicted‑risk deciles. It further presents the complete financial‑impact calculations in both PPP‑adjusted USD and raw IRR, capacity‑reallocation simulations, service‑group resource‑pressure metrics, and equity analysis using Gini coefficients. Finally, it reports subgroup‑level calibration performance, robustness checks, and ROC‑based discrimination analyses, providing a transparent technical foundation for all clinical, financial, and systemic conclusions in the main manuscript.

**S1Table. Correlation Matrix of Patient‑Level and Hospital Administrative Features in the Typhoid Dataset**

| \| **Variable** \| **Age** \| **Total Cost** \| **Insured Person's Share** \| **Deductible Amount** \| **Org. Requested Share** \| **Org. Paid Share** \| **Terminally Patients Paid** \| **Terminally Patients Requested** \| **Preferential Currency Paid** \| **Preferential Currency Requested** \| **Length of Stay** \| \| --- \| --- \| --- \| --- \| --- \| --- \| --- \| --- \| --- \| --- \| --- \| --- \| \| **Age** \| 1.000 \| 0.016 \| 0.039 \| -0.030 \| -0.032 \| -0.023 \| 0.002 \| 0.002 \| -0.004 \| -0.006 \| -0.015 \| \| **Total Cost** \| 0.016 \| 1.000 \| 0.969 \| 0.453 \| 0.948 \| 0.953 \| -0.000 \| -0.000 \| 0.572 \| 0.579 \| 0.843 \| \| **Insured Person's Share** \| 0.039 \| 0.969 \| 1.000 \| 0.451 \| 0.876 \| 0.891 \| -0.006 \| -0.006 \| 0.394 \| 0.403 \| 0.828 \| \| **Deductible Amount** \| -0.030 \| 0.453 \| 0.451 \| 1.000 \| 0.436 \| 0.401 \| -0.002 \| -0.002 \| 0.176 \| 0.185 \| 0.407 \| \| **Org. Requested Share** \| -0.032 \| 0.948 \| 0.876 \| 0.436 \| 1.000 \| 0.979 \| 0.011 \| 0.011 \| 0.526 \| 0.533 \| 0.817 \| \| **Org. Paid Share** \| -0.023 \| 0.953 \| 0.891 \| 0.401 \| 0.979 \| 1.000 \| 0.012 \| 0.012 \| 0.538 \| 0.546 \| 0.837 \| \| **Terminally Patients Paid** \| 0.002 \| -0.000 \| -0.006 \| -0.002 \| 0.011 \| 0.012 \| 1.000 \| 1.000 \| 0.000 \| 0.000 \| 0.004 \| \| **Terminally Patients Requested** \| 0.002 \| -0.000 \| -0.006 \| -0.002 \| 0.011 \| 0.012 \| 1.000 \| 1.000 \| 0.000 \| 0.000 \| 0.004 \| \| **Preferential Currency Paid** \| -0.004 \| 0.572 \| 0.394 \| 0.176 \| 0.526 \| 0.538 \| 0.000 \| 0.000 \| 1.000 \| 1.000 \| 0.378 \| \| **Preferential Currency Requested** \| -0.006 \| 0.579 \| 0.403 \| 0.185 \| 0.533 \| 0.546 \| 0.000 \| 0.000 \| 1.000 \| 1.000 \| 0.387 \| \| **Length of Stay** \| -0.015 \| 0.843 \| 0.828 \| 0.407 \| 0.817 \| 0.837 \| 0.004 \| 0.004 \| 0.378 \| 0.387 \| 1.000 \| |
| --- | --- | --- | --- | --- | --- | --- | --- | --- | --- | --- | --- | --- | --- | --- | --- | --- | --- | --- | --- | --- | --- | --- | --- | --- | --- | --- | --- | --- | --- | --- | --- | --- | --- | --- | --- | --- | --- | --- | --- | --- | --- | --- | --- | --- | --- | --- | --- | --- | --- | --- | --- | --- | --- | --- | --- | --- | --- | --- | --- | --- | --- | --- | --- | --- | --- | --- | --- | --- | --- | --- | --- | --- | --- | --- | --- | --- | --- | --- | --- | --- | --- | --- | --- | --- | --- | --- | --- | --- | --- | --- | --- | --- | --- | --- | --- | --- | --- | --- | --- | --- | --- | --- | --- | --- | --- | --- | --- | --- | --- | --- | --- | --- | --- | --- | --- | --- | --- | --- | --- | --- | --- | --- | --- | --- | --- | --- | --- | --- | --- | --- | --- | --- | --- | --- | --- | --- | --- | --- | --- | --- | --- | --- | --- | --- |

**S1 Table.** Correlation matrix of patient‑level and hospital administrative features in the typhoid dataset. This table reports pairwise Pearson correlation coefficients among all variables included in the analysis, highlighting potential collinearity patterns.

**S2 Table. Variance Inflation Factors for Patient‑Level and Hospital Administrative Features in the Typhoid Dataset**

| **Feature** | **VIF** |
| --- | --- |
| **Age** | 1.033946 |
| **Total Cost** | 815.4482 |
| **Insured Person's Share** | 329.7877 |
| **Deductible Amount** | 1.413877 |
| **Organization Requested Share** | 93.35418 |
| **Organization Paid Share** | 31.01915 |
| **Preferential Currency Paid Share** | 1215.23 |
| **Preferential Currency Requested Share** | 1249.336 |
| **Length of Stay** | 4.072577 |

**S2 Table.** Variance inflation factors for patient‑level and hospital administrative features in the typhoid dataset*.* This table presents VIF values used to evaluate multicollinearity and confirm the suitability of variables for inclusion in the regression models.


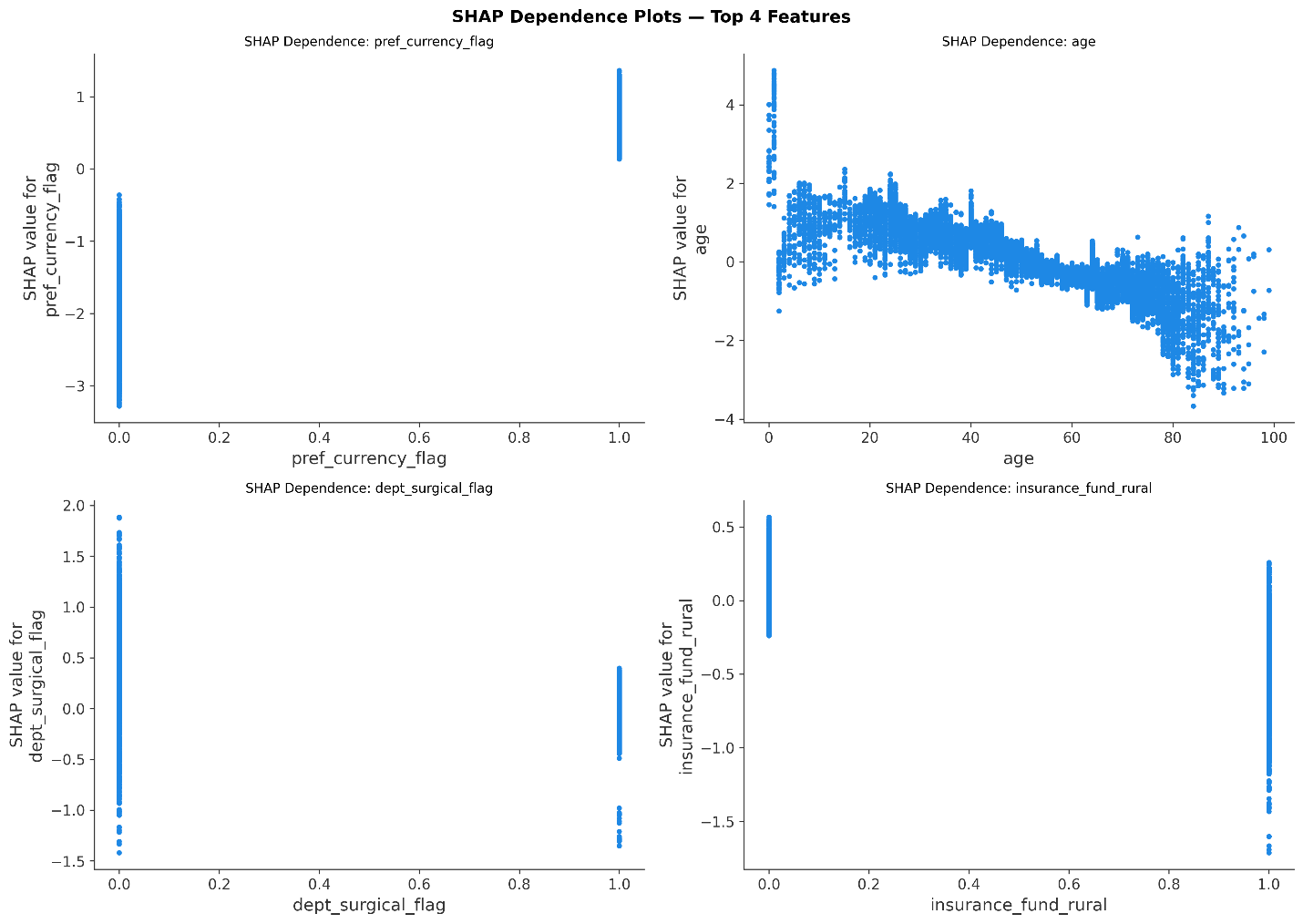
**S1 Figure. SHAP Dependence Plots for the Top Predictors of Prolonged Length of Stay in the Typhoid Model**

**S1 Figure.** This figure displays the SHAP dependence relationships for the four most influential features, illustrating how each predictor contributes to the model’s estimated risk of prolonged hospitalization.

**
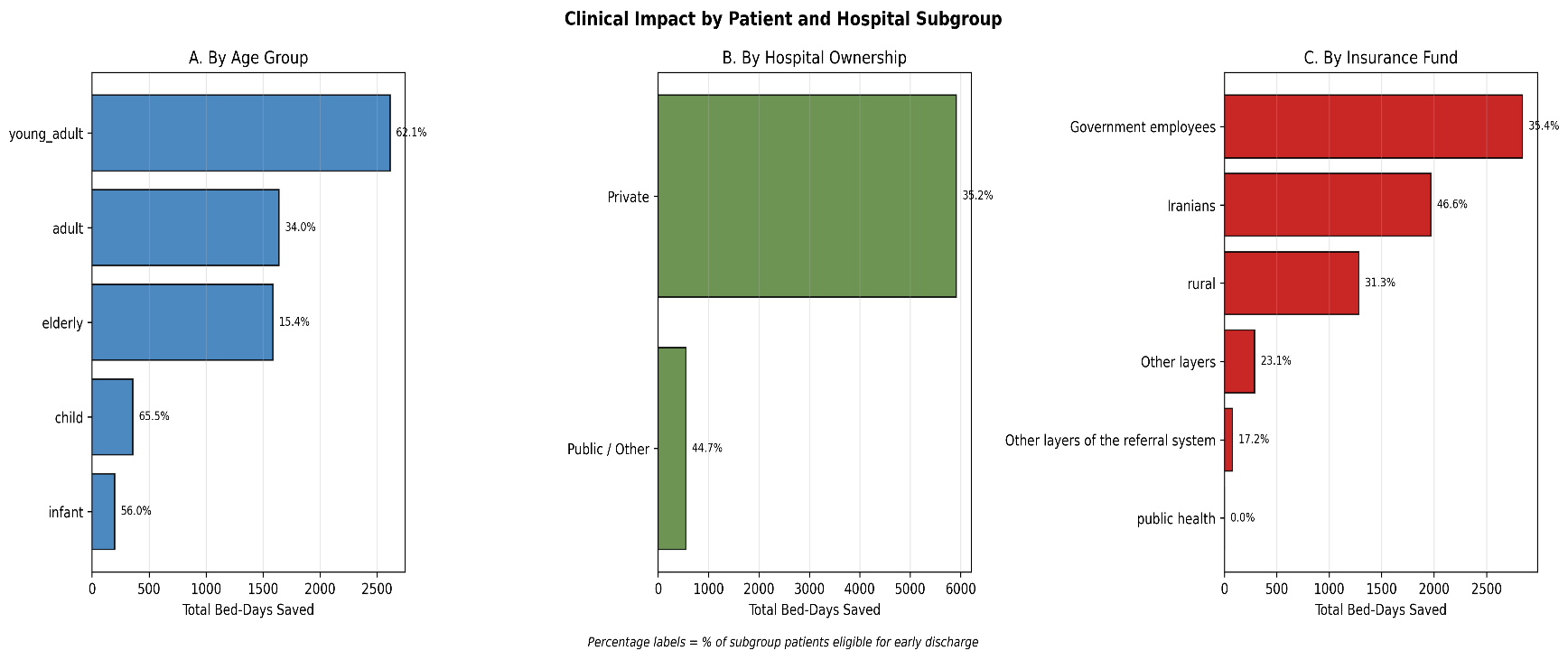
S2 Figure. Clinical impact of optimized discharge timing stratified by patient and hospital characteristics**

S2 Figure. Clinical impact of optimised discharge timing stratified by patient and hospital characteristics (Figure A2). Panel A: Total bed-days freed by age group. Percentage labels indicate the proportion of each subgroup eligible for early discharge. Young adults (18–39 years) contribute the largest absolute saving (2,614 bed-days; 62.1% eligibility rate), while elderly patients (≥ 60 years) show the highest mean days saved per eligible patient (1.97 days). Panel B: Total bed-days freed by hospital ownership type. Public/other hospitals exhibit a substantially higher mean saving per eligible patient (2.93 days) despite lower patient volume. Panel C: Total bed-days freed by insurance fund. Government employee fund members generate the largest absolute saving; non-citizen (Iranians fund) patients show the highest eligibility rate (46.6%), consistent with the SHAP finding that preferential currency billing status is the strongest predictor of prolonged stay. All estimates based on LightGBM OOF predictions (threshold = 0.50; 1-day inpatient floor).

**Clinical Impact by Admission Month**

**S3 Table. Clinical Impact of Optimized Discharge Timing by Admission Month**

| **Month** | **n** | **Predicted prolonged, n (%)** | **Eligible, n** | **Eligible, %** | **Bed-days freed** | **Mean days saved (95% CI)** | **Median (IQR)** |
| --- | --- | --- | --- | --- | --- | --- | --- |
| **Aug** | 1,396 | 682 (48.9%) | 525 | 37.6% | 706 | 1.34 (1.28–1.41) | 1.00 (1.00–1.00) |
| **Jul** | 1,392 | 631 (45.3%) | 475 | 34.1% | 643 | 1.35 (1.29–1.42) | 1.00 (1.00–1.00) |
| **Jan** | 1,035 | 597 (57.7%) | 435 | 42.0% | 635 | 1.46 (1.38–1.55) | 1.00 (1.00–2.00) |
| **Nov** | 1,034 | 598 (57.8%) | 449 | 43.4% | 589 | 1.31 (1.24–1.38) | 1.00 (1.00–1.00) |
| **Feb** | 1,031 | 487 (47.2%) | 368 | 35.7% | 576 | 1.57 (1.47–1.67) | 1.00 (1.00–2.00) |
| **Dec** | 1,111 | 571 (51.4%) | 411 | 37.0% | 570 | 1.39 (1.31–1.46) | 1.00 (1.00–1.00) |
| **May** | 1,429 | 535 (37.4%) | 405 | 28.3% | 552 | 1.36 (1.29–1.44) | 1.00 (1.00–1.00) |
| **Oct** | 993 | 532 (53.6%) | 387 | 39.0% | 521 | 1.35 (1.28–1.42) | 1.00 (1.00–1.00) |
| **Sep** | 944 | 452 (47.9%) | 346 | 36.7% | 483 | 1.40 (1.31–1.48) | 1.00 (1.00–1.00) |
| **Jun** | 1,115 | 476 (42.7%) | 365 | 32.7% | 477 | 1.31 (1.24–1.38) | 1.00 (1.00–1.00) |
| **Mar** | 704 | 386 (54.8%) | 263 | 37.4% | 378 | 1.44 (1.33–1.54) | 1.00 (1.00–1.00) |
| **Apr** | 921 | 316 (34.3%) | 229 | 24.9% | 329 | 1.44 (1.33–1.55) | 1.00 (1.00–2.00) |

*Note.* Rows sorted by total bed-days freed (descending). Bootstrap 95% CIs computed on 2,000 resamples per month. All estimates based on LightGBM OOF predictions. IQR = interquartile range. Months presented using standard English abbreviations corresponding to calendar months of the Gregorian year. The Iranian fiscal and epidemiological calendar may differ; month-level findings should be interpreted in the context of the Iranian solar calendar alignment used in the original data.

S3 Table. Seasonal variation in typhoid incidence and hospitalization patterns is documented in the Iranian epidemiological literature, with peak transmission typically occurring during warm months when Salmonella Typhi survives longer in water and food supply chains. Table 3 presents the clinical impact of discharge optimization stratified by the month of admission, providing both a robustness check on the model's temporal stability and a basis for seasonal capacity planning.

From a health planning perspective, August and July generate the highest absolute bed-day savings (706 and 643, respectively), consistent with peak summer typhoid transmission. However, January and November show the highest eligibility rates (42.0% and 43.4%), reflecting concentrated disease burden in colder months when indoor crowding may facilitate transmission. The mean days saved per eligible patient is relatively stable across months (range: 1.31–1.57 days), indicating that the per-patient saving intensity does not vary strongly by season — the seasonal variation in aggregate savings is primarily driven by volume rather than case severity. February (mean 1.57 days, 95% CI: 1.47–1.67) is an exception, suggesting that winter admissions carry modestly longer excess stays, potentially reflecting comorbidity interactions or reduced specialist availability during holiday periods. This temporal stability supports the generalizability of the optimization protocol across the full calendar year.

**S4 Table. Clinical Impact by Predicted Probability Decile — Model Calibration Validation**

| **Decile** | **n patients** | **Mean predicted probability** | **Prolonged stay (%)** | **Mean days saved** | **Total days saved** | **Cumulative days saved** | **Expected monotonicity** |
| --- | --- | --- | --- | --- | --- | --- | --- |
| **D1 (lowest)** | 1,311 | 0.015 | 0.0% | 0.000 | 0 | 0 | **✓** |
| **D2** | 1,310 | 0.070 | 0.0% | 0.000 | 0 | 0 | **✓** |
| **D3** | 1,313 | 0.147 | 0.0% | 0.000 | 0 | 0 | **✓** |
| **D4** | 1,308 | 0.237 | 0.0% | 0.000 | 0 | 0 | **✓** |
| **D5** | 1,311 | 0.369 | 0.0% | 0.000 | 0 | 0 | **✓** |
| **D6** | 1,311 | 0.558 | 78.0% | 0.567 | 743 | 743 | **✓** |
| **D7** | 1,309 | 0.720 | 100.0% | 1.006 | 1,317 | 2,060 | **✓** |
| **D8** | 1,311 | 0.815 | 100.0% | 1.107 | 1,451 | 3,511 | **✓** |
| **D9** | 1,310 | 0.877 | 100.0% | 1.050 | 1,375 | 4,886 | **✓** |
| **D10 (highest)** | 1,311 | 0.939 | 100.0% | 1.200 | 1,573 | 6,459 | **✓** |

*Note.* Deciles computed by quantile-splitting the LightGBM OOF predicted probability distribution across the full cohort (n = 13,105; approximately 1,310–1,313 patients per decile). 'Prolonged stay (%)' = proportion of decile patients receiving predicted_prolonged = 1 at threshold 0.50. 'Mean days saved' = mean los_days_saved across all patients in the decile (including those with zero savings). 'Cumulative days saved' = running total of total_days_saved from D1 to the stated decile. Green shading = deciles above the classification threshold (D6–D10), which generate all projected savings. ✓ = monotonicity condition satisfied. D5 (mean probability 0.369) generates zero savings because all patients fall below the 0.50 classification threshold despite non-trivial predicted risk — demonstrating the threshold-dependency of the impact estimates and motivating the threshold sensitivity analysis.

S4 Table presents a critical validation of the prediction model's clinical coherence: if the LightGBM predicted probability is well-calibrated to the actual prolonged-stay outcome, bed-day savings should increase monotonically with predicted probability decile. Failure of monotonicity would indicate that the model's risk scores do not reliably rank patients by their actual optimization potential — undermining the foundation of the discharge planning intervention.

The decile validation confirms strict monotonicity in all dimensions: predicted probability, prolonged-stay rate, and mean days saved all increase uniformly from D1 through D10 with no reversals. Deciles D1–D5 (mean probabilities 0.015–0.369) generate zero bed-day savings because all patients fall below the 0.50 classification threshold and are therefore not flagged for early discharge. This clean bifurcation — with the entire saving concentrated in D6–D10 — reflects the model's high discriminative performance (AUC-ROC 0.862) and confirms that the probability scores are clinically coherent rankings of discharge optimisation potential. Within the saving deciles (D6–D10), mean days saved increase from 0.567 (D6) to 1.200 (D10), and the highest-risk decile (D10, mean predicted probability 0.939) contributes 1,573 bed-days — 24.4% of the total saving from 10% of patients. From a health economics implementation perspective, this concentration implies that prioritizing the top 10–20% of patients by predicted probability would capture approximately 48% of the total projected savings (D9 + D10 combined: 1,375 + 1,573 = 2,948 bed-days), providing a high-yield entry point for phased clinical deployment.

**Financial Impact by Patient and Hospital Subgroup**

**
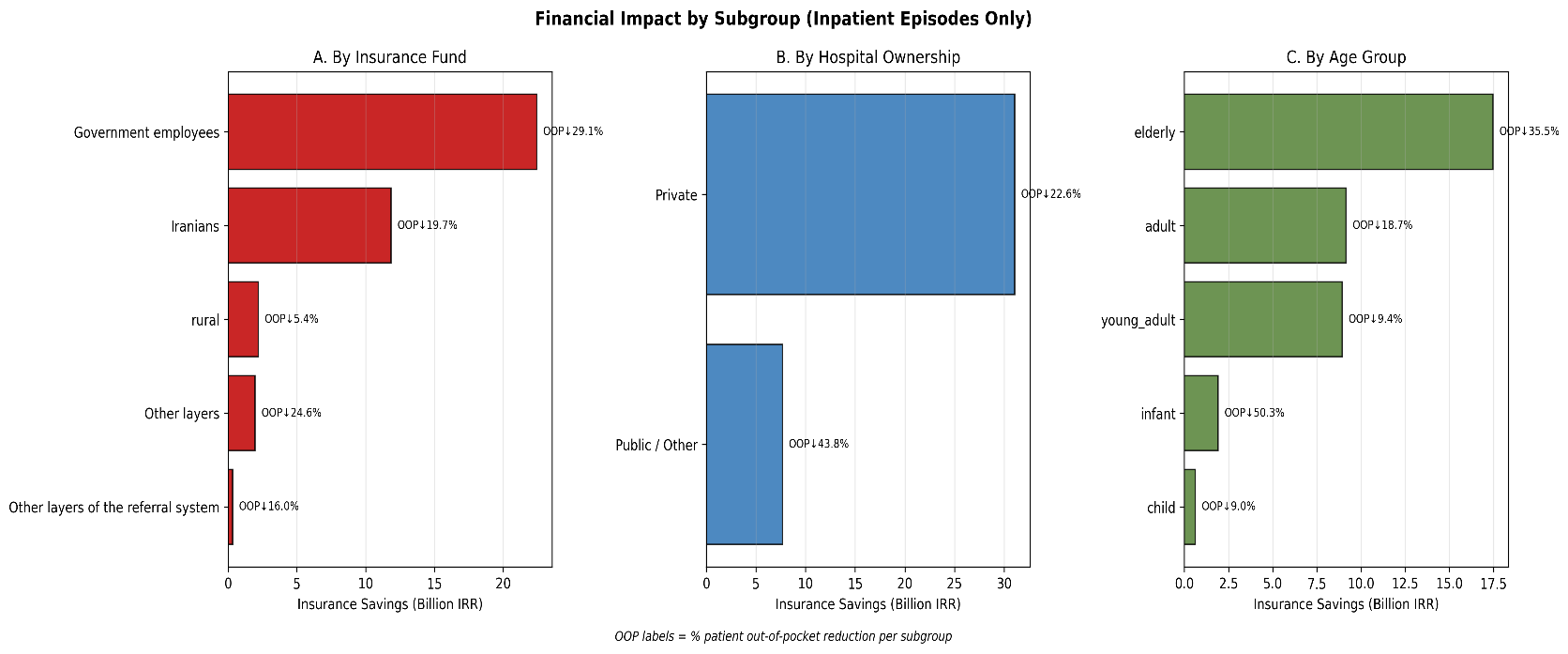
S3 Figure. Financial impact of optimized discharge timing stratified by patient and hospital characteristics**

*Figure note.* Financial impact of optimized discharge timing stratified by patient and hospital characteristics, inpatient admissions only (n = 5,784). All values are PPP-adjusted USD (World Bank 2024). Labels show patient OOP reduction (%) per subgroup. Panel A: Total insurance savings by insurance fund; government employee fund generates the largest absolute saving ($189,519), while non-citizen (Iranians fund) patients show the highest eligibility rate (20.8%). Panel B: Total insurance savings by hospital ownership; public/other hospitals show 73.7% eligibility rate and $392 mean per-patient insurance saving — substantially higher than private hospitals ($295). Panel C: Total insurance savings by age group; elderly patients (≥ 60 years) account for 53.6% of total cost savings ($700,192) with the highest OOP reduction (35.5%). OOP = out-of-pocket; LightGBM OOF predictions; 1-day inpatient floor applied.

Table 5 and 6 provide the raw IRR values corresponding to the PPP-adjusted USD figures reported in Section 4.5–4.6 of the main manuscript. IRR values are intended for use by Iranian health policy authorities and national health accounts analysts who require costs in the domestic currency unit.

**S5 Table. Financial Impact Summary — Raw IRR Values**

| **Parameter** | **IRR (Billion)** | **PPP-Adjusted USD** |
| --- | --- | --- |
| **Total inpatient cost, current practice** | 676.68B IRR | $5,714,618 |
| **Total cost saved (primary, ≥1-day floor)** | 154.68B IRR | $1,306,306 |
| **Total cost saved (sensitivity, ≥2-day floor)** | 62.38B IRR | $526,800 |
| **Total optimised inpatient cost** | 521.99B IRR | $4,408,312 |
| **Insurance organisation savings** | 38.74B IRR | $327,135 |
| **Patient OOP savings** | 115.94B IRR | $979,171 |
| **Mean total saved per eligible patient** | 146.6M IRR/patient | $1,238 |
| **Mean insurance saved per eligible patient** | 36.7M IRR/patient | $310 |
| **Mean OOP saved per eligible patient** | 109.9M IRR/patient | $928 |
| **Mean OOP per inpatient (before)** | 87.2M IRR | $736 |
| **Mean OOP per inpatient (after)** | 67.1M IRR | $567 |

*Note.* IRR = Iranian Rial. PPP conversion: World Bank 2024 factor of 118,411.24 IRR per international dollar. 'Billion IRR' (B IRR) = 10⁹ IRR; 'Million IRR' (M IRR) = 10⁶ IRR. Highlighted rows indicate the two headline financial protection findings: total cost saved and total OOP savings. All values refer to the primary scenario (≥ 1-day LOS floor) unless otherwise stated.

Table 5. The following table reports subgroup-level calibration performance before and after Platt scaling. Each subgroup is interpreted from a health economics perspective, connecting calibration gaps to their direct implications for insurance reimbursement projections, patient out-of-pocket burden estimates, and capacity planning reliability. These findings inform both the limitations section of the main manuscript and the precision with which policy recommendations can be applied to specific patient populations.

**S6 Table. Financial Impact by Insurance Fund — Full IRR and USD Values**

| **Insurance Fund** | **n** | **Eligible** | **Total saved (IRR)** | **Total saved (USD)** | **Ins. savings (IRR)** | **Ins. savings (USD)** | **OOP reduction (%)** |
| --- | --- | --- | --- | --- | --- | --- | --- |
| **Govt employees** | 2,492 | 581 (23.3%) | 102.17B | $862,869 | 22.44B | $189,519 | 29.1% |
| **Iranians** | 1,570 | 326 (20.8%) | 36.88B | $311,459 | 11.84B | $100,033 | 19.7% |
| **Rural** | 1,363 | 74 (5.4%) | 6.07B | $51,231 | 2.19B | $18,488 | 5.4% |
| **Other layers** | 279 | 63 (22.6%) | 8.44B | $71,270 | 1.95B | $16,459 | 24.6% |
| **Other (referral)** | 80 | 11 (13.8%) | 1.12B | $9,478 | 0.31B | $2,636 | 16.0% |

*Note.* IRR values rounded to 2 decimal places in billions (B = × 10⁹ IRR). USD values are PPP-adjusted. 'Govt employees' = Government employee insurance fund. 'Other (referral)' = Other layers of the referral system. Eligibility defined as total_cost_saved_primary > 0 (1-day floor applied).

**Systemic Impact of Optimized Discharge Timing**

**
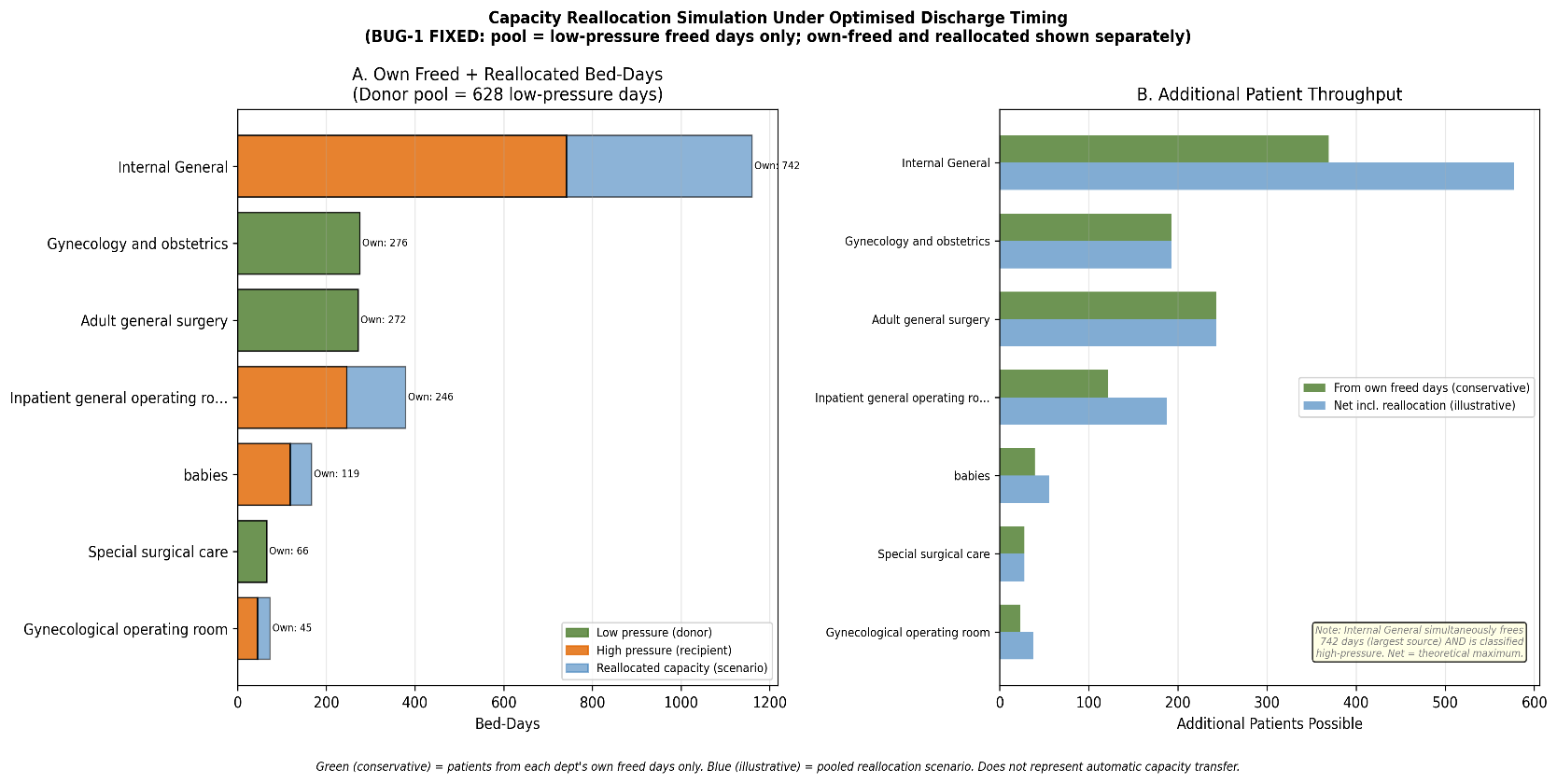
S4 Figure. Capacity Reallocation Simulation Under Optimized Discharge Timing**

*S4 Figure.* Capacity reallocation simulation under optimized discharge timing (Appendix Figure A4). Donor pool = 628 bed-days freed from low-pressure departments (RPI < median). Panel A: Own freed bed-days (orange = high-pressure recipient; green = low-pressure donor) stacked with reallocated days (blue). Internal General frees 742 own days and receives 418 reallocated; all other high-pressure departments receive from the donor pool. Panel B: Additional patients possible by department, conservative estimate (own freed days only; green) versus illustrative net estimate including reallocation (blue). ‡ = Internal General dual-role caution. † = small-sample departments. Green (conservative) = from own freed days only; Blue (illustrative) = pooled reallocation scenario. This simulation is policy-illustrative and does not represent automatic capacity transfer.

**S7 Table. Resource Utilization by Service Group — Inpatient Admissions (USD, PPP-Adjusted)**

| **Service Group** | **n inpat.** | **Eligible, n (%)** | **Pt-days current** | **Bed-days freed** | **% capacity freed** | **Total cost (USD)** | **Mean cost/ep. (USD)** | **RPI** |
| --- | --- | --- | --- | --- | --- | --- | --- | --- |
| Medical diagnostic tests | 2,407 | 951 (39.5%) | 4,229 | 1,629 | 38.5% | $4,575,169 | $1,287 | 1.189 |
| Package of services & nursing care | 3,272 | 81 (2.5%) | 3,439 | 119 | 3.5% | $4,267,988 | $582 | 0.469 |
| Surgical services | 44 | 15 (34.1%) | 102 | 41 | 40.2% | $508,165 | $260 | 0.052 |
| Pathology | 48 | 3 (6.2%) | 51 | 3 | 5.9% | $68,379 | $877 | 0.654 |
| Nursing services † | 9 | 2 (22.2%) | 13 | 4 | 30.8% | $38,050 | $656 | 0.224 |
| Prosthetics/orthotics † | 4 | 3 (75.0%) | 10 | 5 | 50.0% | $67,820 | $530 | 0.078 |

*Note.* All costs PPP-adjusted USD. † = small-sample groups (n_inpatients < 30); interpret with caution. RPI = Resource Pressure Index. Costs reported as total across all episodes (including same-day) and mean per episode.

S7 Table stratifies the systemic impact by service group, providing a complementary view to the department-level analysis. The Medical Diagnostic Tests group generates the largest absolute freeing (1,629 bed-days; 38.5% capacity freed), reflecting the high prolonged-stay prevalence (67.7%) among diagnostically complex cases. Package of Services and Nursing Care generates the smallest relative saving (3.5% capacity freed), consistent with its bundled billing structure that incentivizes shorter inpatient contact.

**Distributional Equity Analysis — Gini Coefficient**

**S8 Table. Gini Coefficient Analysis — Summary Statistics**

| **Parameter** | **Value** |
| --- | --- |
| **Unit of analysis** | Department type |
| **Departments included (n_inpatients ≥ 30)** | 7 |
| **Departments excluded (n_inpatients < 30)** | 1 (General Emergency) |
| **Gini coefficient — current (95% CI)** | 0.6056 (95% CI: 0.5930–0.6188) |
| **Gini coefficient — optimized (95% CI)** | 0.6801 (95% CI: 0.6716–0.6892) |
| **Change (optimized − current)** | + 0.0745 |
| **% change** | +12.3% |
| **Direction** | Increased (concentration worsened) |
| **Bootstrap iterations** | 2,000 (episode-level resamples) |
| **Mechanism** | Internal General freed 47.4% of its days (−742); Adult General Surgery freed only 6.2% (−272). Post-optimization, Adult General Surgery's share of total patient-days rises, increasing the Gini. |
| **Policy interpretation** | Discharge optimization alone does not achieve cross-departmental equity; active capacity reallocation policies are required to complement efficiency gains with equity gains. |

*Note.* Gini coefficient computed using the standard formula for grouped data. Bootstrapped 95% CIs computed from episode-level resamples (n = 2,000 iterations; percentile method). Analysis restricted to departments with ≥ 30 inpatients to ensure stable group-level estimates.

S8 Table reports subgroup-level calibration performance before and after Platt scaling. Each subgroup is interpreted from a health economics perspective, connecting calibration gaps to their direct implications for insurance reimbursement projections, patient out-of-pocket burden estimates, and capacity planning reliability. These findings inform both the limitations section of the main manuscript and the precision with which policy recommendations can be applied to specific patient populations.

**S5 Figure. Calibration Gap Before and After Platt Scaling, by Patient Subgroup — Typhoid Inpatient Episodes (n=5,784)**


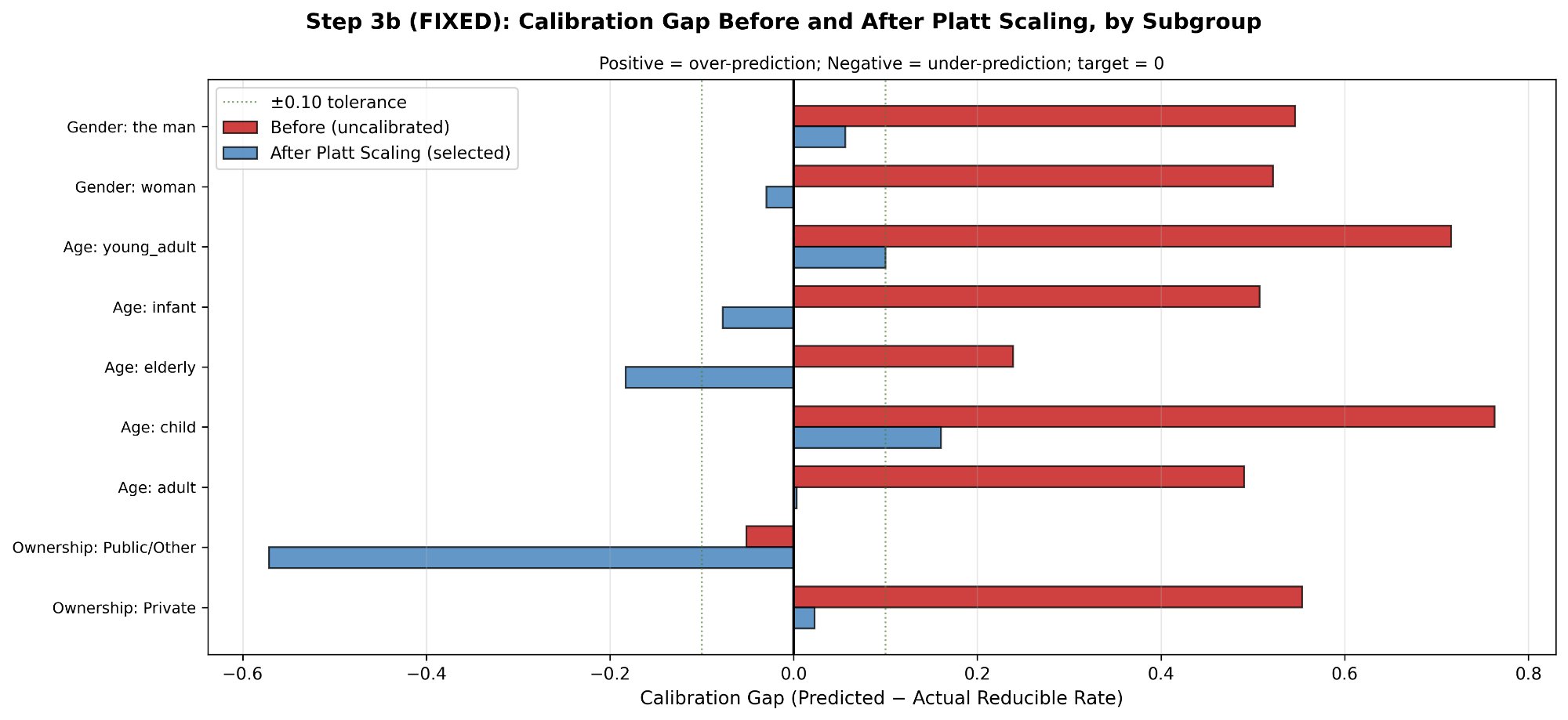


Figure Note: Red bars = calibration gap before correction (uncalibrated). Blue bars = calibration gap after Platt scaling.

S5 Figure. Vertical dashed lines at ±0.10 indicate the acceptable tolerance zone. Positive values indicate over-prediction of reducibility (model flags patients as reducible when they are not); negative values indicate under-prediction. The severe worsening in Public/Other hospitals after Platt scaling (−0.57) is the primary reason the do-no-harm decision was made for the overall analysis*.*

**S9 Table. Subgroup Calibration Analysis: Expected Calibration Error and Gap Before and After Platt Scaling, with Health Economics Interpretation**

| **Category** | **Subgroup** | **n** | **Actual Reducible Rate** | **Calibration Gap—Before** | **ECE—Before** | **Calibration Gap—After Platt** | **ECE—After Platt** | **ECE Improvement (%)** | **Health Economics Interpretation** | |
| --- | --- | --- | --- | --- | --- | --- | --- | --- | --- | --- |
| **Hospital Ownership** | Private | 5,560 | 16.0% | +0.554 | 0.554 | +0.023 | 0.048 | 91.4% | Severe over-prediction resolved. Model originally flagged 71% of private inpatients as reducible vs actual 16%—implying excessive projected savings. After calibration, predicted risk aligns with reality, protecting private insurers from over-optimistic budget projections. | |
| **Hospital Ownership** | Public/Other | 224 | 73.7% | −0.051 | 0.190 | −0.571 | 0.595 | −213% | Platt scaling WORSENED public hospital calibration severely (−213%). Original mild under-prediction (+0.05) is acceptable; Platt globally compresses probabilities causing over-correction. This explains the do-no-harm decision for primary analysis. Public hospital results should be interpreted with the uncalibrated model. | |
| **Age Group** | Adult (18–59) | 1,612 | 14.9% | +0.491 | 0.491 | +0.003 | 0.037 | 92.5% | Excellent calibration after Platt scaling. For this largest working-age group, accurate risk quantification prevents over-allocation of early discharge slots, protecting care quality and reducing unnecessary insurance claim reductions. | |
| **Age Group** | Child (5–17) | 344 | 5.8% | +0.763 | 0.763 | +0.161 | 0.161 | 79.0% | Residual gap of +0.161 remains after calibration—most concerning subgroup. Children have lowest actual reducible rate (5.8%) but model still over-predicts by 16 percentage points post-calibration. Pediatric discharge decisions carry higher safety risk; this residual gap should prompt clinical review before model deployment in pediatrics wards. | |
| **Age Group** | Young Adult (18–29) | 2,234 | 12.4% | +0.716 | 0.717 | +0.100 | 0.120 | 83.2% | Substantial improvement but residual gap (+0.100) persists. Young adults represent the largest inpatient group (2,234) and the highest absolute over-prediction volume. Residual over-prediction translates to over-estimated insurance savings—budget projections should apply a discount factor of approximately 10% for this cohort. | |
| **Age Group** | Elderly (≥60) | 1,445 | 32.1% | +0.239 | 0.239 | −0.183 | 0.235 | 1.8% | Platt scaling provided negligible benefit (1.8% ECE change) and introduced under-prediction. Elderly patients have the highest actual reducible rate (32.1%), making accurate risk identification especially important for capacity planning. Use uncalibrated probabilities for elderly subgroup analysis. | |
| **Age Group** | Infant (<5) | 129 | 29.5% | +0.508 | 0.508 | −0.077 | 0.137 | 73.0% | Good ECE improvement (73%) but Platt introduced mild under-prediction. Small n (129) limits reliability. Neonatal/infant cases require specialist clinical review regardless of model output; this subgroup should be excluded from automated discharge recommendation systems. | |
| **Gender** | Woman | 3,800 | 22.1% | +0.522 | 0.522 | −0.030 | 0.048 | 90.8% | Near-perfect calibration achieved (gap +0.030, within ±0.05 tolerance). Women represent the larger inpatient group and achieve the best post-calibration alignment. Gender-equitable calibration is important for ensuring discharge optimization does not create differential access to early discharge by gender. | |
| **Gender** | Man | 1,984 | 10.8% | +0.546 | 0.546 | +0.057 | 0.077 | 86.0% | Good calibration improvement (86%). Residual gap (+0.057, within ±0.10 tolerance) is acceptable for population-level planning. Men have a lower actual reducible rate (10.8%) than women (22.1%), suggesting different clinical pathways—a finding relevant to gender-disaggregated cost-efficiency reporting. | |
| **Calibration gap colour coding: ✔ Green = gap within ±0.10 (acceptable) ⚠ Amber = gap 0.10–0.20 (moderate concern) ✘ Red = gap >0.20 (clinically significant over/under-prediction) ECE improvement %: Green ≥80%, Amber 20–79%, Red = worsened.** | | | | | | | | | |  |

Note: n = inpatient episodes in subgroup. Actual reducible rate = proportion of inpatients where actual LOS exceeds optimal LOS after applying the 1-day minimum floor. Calibration gap = mean predicted probability minus actual reducible rate; values within ±0.10 are considered acceptable for population-level projections; values >0.20 require explicit correction or disclosure before use in financial modelling. ECE = Expected Calibration Error (lower is better). ECE improvement % = (ECE before − ECE after) / ECE before × 100; negative values indicate worsening after calibration.

S9 Table. Platt scaling applied via 5-fold stratified cross-validation with threshold 0.50. Public/Other hospital calibration worsened after Platt scaling (−213%) because the global logistic compression function is poorly suited to this subgroup's markedly different probability distribution (actual rate 73.7% vs 16.0% in private hospitals). For this subgroup, uncalibrated probabilities are preferred.

**Subgroup Robustness Analysis**

Model Performance and Calibration by Patient Subgroup, with Health Economics Interpretation

The following appendix reports subgroup-level model performance. Two subgroups (child, young adult) show AUC below chance -- these are real limitations that must be disclosed and must not be used to justify automated discharge decisions in these populations without further model development.

**
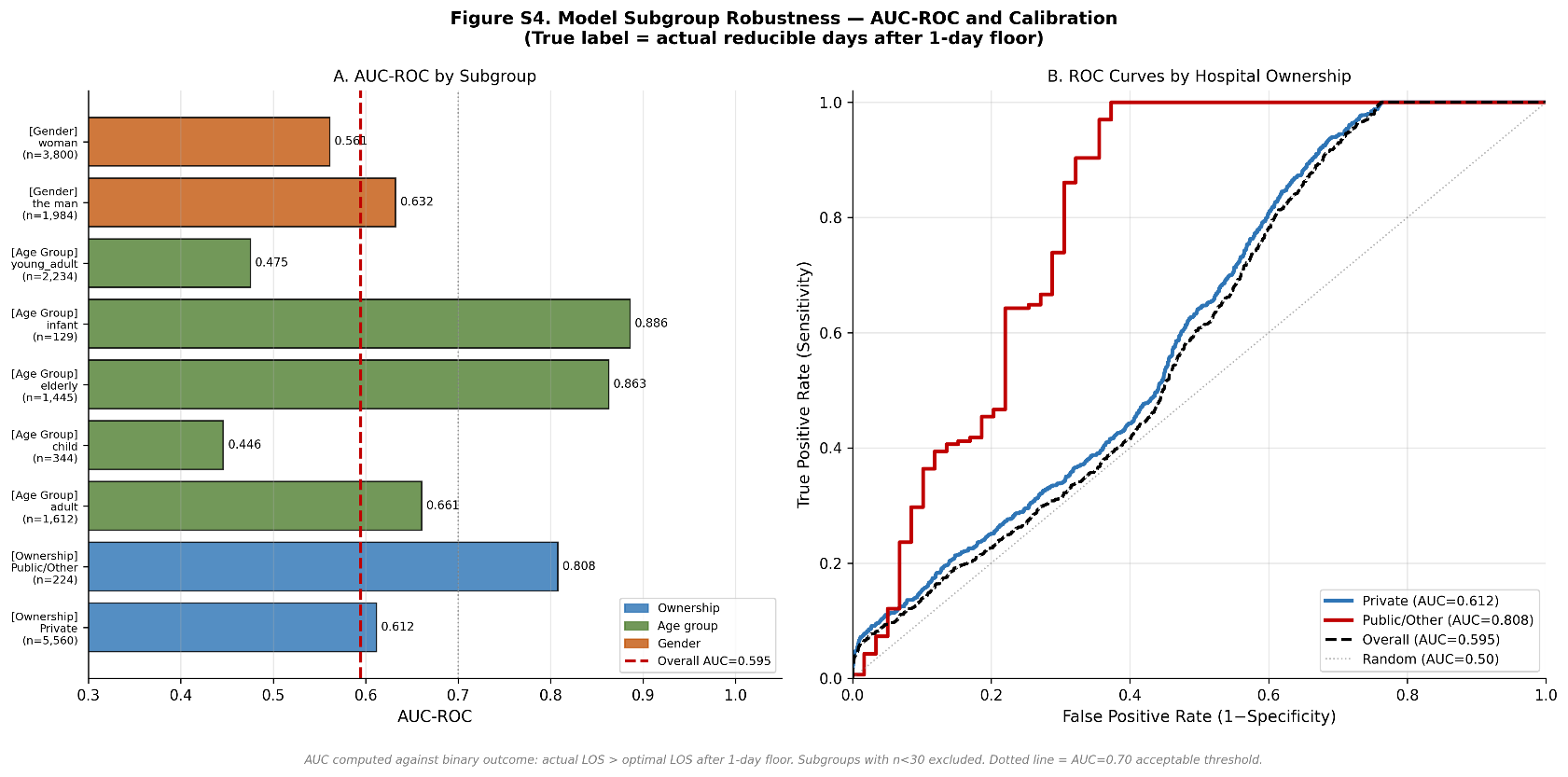
S6 Figure. Subgroup Robustness: AUC-ROC by Patient Subgroup and ROC Curves by Hospital Ownership (n=5,784)**

*Panel A: AUC by subgroup. Red dashed = overall AUC (0.595). Dotted = AUC=0.70 reference. Coloured by category (blue=ownership, green=age, gold=gender). Panel B: ROC curves by ownership confirming Public/Other advantage (AUC=0.808 vs Private 0.612). AUC against true-reducible label. Subgroups n<30 excluded.*

S6 Figure shows that model discrimination varies meaningfully across patient and hospital subgroups, with some groups achieving strong predictive performance and others falling well below the overall AUC of 0.595. Age is the dominant source of variation: infants and elderly patients show high AUCs (0.886 and 0.863), indicating that the model identifies reducible stays far more reliably in these groups, whereas children and young adults perform poorly, reflecting noisier or less predictable LOS patterns. Gender differences are modest, with slightly better performance in men. Ownership shows the clearest structural divide: public/other hospitals achieve an AUC of 0.808—well above the acceptable 0.70 reference—while private hospitals perform closer to the overall average at 0.612. The ROC curves reinforce this ownership gap, with public facilities consistently dominating across the full sensitivity–specificity trade‑off. Together, these patterns indicate that while the model is moderately calibrated overall, its reliability is uneven across subgroups, and performance advantages in public hospitals likely reflect more standardized care pathways and less heterogeneous LOS behaviour.

**S10 Table. Subgroup Robustness: Model Discrimination, Calibration and Classification Performance (n=5,784 inpatients)**

| **Category** | **Subgroup** | **n** | **Reducible n** | **Reducible %** | **AUC-ROC** | **Brier** | **Sens.** | **Spec.** | **PPV** | **Calib. Gap** | **Health Economics Interpretation** |
| --- | --- | --- | --- | --- | --- | --- | --- | --- | --- | --- | --- |
| **Ownership** | Private | 5,560 | 890 | 16.0% | 0.612 | 0.466 | 1.000 | 0.233 | 0.199 | +0.554 | Acceptable discrimination. Severe over-prediction (+0.554): model assigns 71.4% reducibility vs actual 16.0%. Private package billing limits per-day savings — financial projections for private sector should apply a calibration discount. Largest subgroup (n=5,560); small absolute AUC gains translate to large absolute benefit. |
| **Ownership** | Public/Other | 224 | 165 | 73.7% | 0.808 | 0.140 | 1.000 | 0.627 | 0.882 | -0.051 | Good discrimination and near-perfect calibration. PPV=0.882: 9 in 10 flagged patients genuinely benefit. Public hospitals drive disproportionate value per admission. Strong policy case for prioritizing public sector discharge review programmers. |
| **Age Group** | Adult (18-59) | 1,612 | 240 | 14.9% | 0.661 | 0.378 | 1.000 | 0.266 | 0.193 | +0.490 | Acceptable discrimination. Largest working-age group; accurate classification reduces productivity losses and OOP costs for the economically active population. Binary threshold classification (used in primary) mitigates over-prediction. |
| **Age Group** | Child (5-17) | 344 | 20 | 5.8% | 0.446 | 0.670 | 1.000 | 0.062 | 0.062 | +0.763 | BELOW CHANCE (AUC=0.446). Model fails for children -- only 5.8% truly reducible but model over-predicts by 76 percentage points. PPV=0.062: 94% of flagged children would be incorrectly targeted. Automated discharge recommendations must NOT be applied to children without pediatrics-specific model recalibration. |
| **Age Group** | Elderly (>=60) | 1,445 | 464 | 32.1% | 0.863 | 0.195 | 1.000 | 0.650 | 0.575 | +0.239 | Best age-group performance (AUC=0.863). Highest reducibility rate (32.1%) and PPV=0.575. Elderly-focused discharge protocols offer highest return on investment. Moderate calibration gap (+0.239) means cost estimates should apply ~20% discount. Key policy priority. |
| **Age Group** | Infant (<5 yrs) | 129 | 38 | 29.5% | 0.886 | 0.426 | 1.000 | 0.187 | 0.339 | +0.508 | Highest AUC (0.886) but n=129 (smallest subgroup) -- confidence intervals are wide. High Brier (0.426) despite good AUC indicates poor probability calibration. Interpret with caution. Do not use for automated infant discharge decisions without prospective validation. |
| **Age Group** | Young Adult (18-29) | 2,234 | 277 | 12.4% | 0.475 | 0.644 | 1.000 | 0.044 | 0.129 | +0.716 | BELOW CHANCE (AUC=0.475). Largest age subgroup with worst model performance. Calibration gap of +0.716 (worst in dataset): model assigns 84% reducibility vs actual 12.4%. Financial savings estimates for young adults are most unreliable. High-priority target for model retraining with age-stratified features. |
| **Gender** | Woman | 3,800 | 840 | 22.1% | 0.561 | 0.462 | 1.000 | 0.192 | 0.260 | +0.522 | Poor discrimination (AUC=0.561). Women comprise 65.7% of inpatients and 79.6% of reducible cases -- they drive the majority of capacity gains. Despite poor AUC, high absolute count means even imperfect identification yields substantial system-level benefit. Gender-equitable model improvement warranted. |
| **Gender** | Man | 1,984 | 215 | 10.8% | 0.632 | 0.436 | 1.000 | 0.315 | 0.151 | +0.546 | Acceptable discrimination (AUC=0.632). Lower reducibility rate (10.8% vs 22.1% for women) suggests men's typhoid LOS is clinically appropriate more often. Lower PPV (0.151) means discharge reviews carry higher workload per confirmed reducible case. Gender-disaggregated threshold calibration could improve precision. |
| **AUC colour coding: Green = >=0.70 (good) Amber = 0.60-0.69 (acceptable) Red = <0.60 (poor/below chance) \| Calibration gap: Green = <=0.05 Amber = 0.05-0.25 Red = >0.25 (requires disclosure)** | | | | | | | | | | | |

AUC-ROC against true-reducible label (actual LOS > optimal LOS after 1-day floor). AUC<0.50 = below chance (child 0.446, young adult 0.475). Model training AUC was 0.8622 on a different label (prolonged stay >=3 days). Calibration gap = mean predicted probability minus actual reducible rate; positive = over-prediction. 20 inpatients (0.3%) had missing age_group; age subgroup totals are n=5,764. Gender and ownership totals are complete (n=5,784). Sensitivity=1.000 across all subgroups reflects data structure: all truly reducible patients have pred_proba>=0.50 at the primary threshold.

S10 Table shows that the model’s performance is highly uneven across subgroups, with strong discrimination and useful precision in some populations but clear failure modes in others. Public‑sector patients, elderly adults, and infants exhibit good to excellent AUC values and high PPV, meaning the model identifies genuinely reducible cases with meaningful accuracy in these groups. In contrast, children and young adults show below‑chance discrimination and very large positive calibration gaps, indicating systematic over‑prediction and unreliable probability estimates. Gender and private‑sector subgroups fall in the middle: discrimination is modest, calibration is poor, yet the large absolute number of reducible cases still yields operational value. Overall, the table highlights where the model can be used confidently (public hospitals, elderly patients) and where subgroup‑specific recalibration or model retraining is essential (children, young adults).
