# Supplementary material for: Machine learning–optimized discharge timing in typhoid care: Implications for clinical outcomes, cost efficiency, and health system performance
Source: PLoS One. 2026 Jul 23;21(7):e0354148. doi: 10.1371/journal.pone.0354148 (PMC13395383; doi:10.1371/journal.pone.0354148)
Supplement: Attachment — Submitted filename: Response to Reviewers.pdf [file pone.0354148.s003.pdf]

Dear Editor and Reviewers,

Thank you very much for the thoughtful and constructive review of our manuscript (PONE-D-26-09410) entitled *"Machine Learning–Optimized Discharge Timing in Typhoid Care: Implications for Clinical Outcomes, Cost Efficiency, and Health System Performance."* We greatly appreciate the reviewers' time and expertise.

We have carefully revised the manuscript to address all mandatory journal requirements, statistical concerns, clinical validity questions, and formatting issues. Below we provide a point-by-point response. All line numbers refer to the **Revised and Marked up Manuscripts**.

Response to Journal Mandatory Requirements

1. Ethics statement location

Comment: Move ethics statement from end of paper into Methods section.

Response: Done. The ethics approval statement has been moved from its previous location after the Conclusion to the **end of the Methods section** (lines 245–255). The original location has been deleted.

2. Data availability statement

Comment: Justify restrictions and provide a formal statement.

Response: We have revised the Data Availability statement in both the manuscript and the submission system to read:

*The data used in this study contain sensitive national health insurance records governed by Iranian data protection regulations. Access is controlled by the Iran Health Insurance Organization (IHIO) and restricted under ethical approval IR.TUMS.NIHR.REC.1403.015. Data cannot be publicly shared due to legal and ethical constraints. Requests for data access may be directed to the Iran Health Insurance Organization or to the corresponding author (shekoufehmomahhed@gmail.com), who serves as the designated data access coordinator. The analytical code is openly available at:*https://github.com/Shekoofeh31/Machine-Learning-Optimized-Discharge-Timing-in-Typhoid-Care. *A **README file** has been added to the repository to document the code structure, dependencies, execution steps, and instructions for reproducing the analyses reported in this manuscript.*

This statement now appears in the manuscript (lines 1230–1240) and has been entered into the journal’s online submission form.

3. Supporting Information captions

Comment: Add formal captions for supplementary figures and tables.

Response: Done. We have added a complete **Supporting Information section** at the end of each steps of the results section inside the main manuscript with numbered captions for S1–S10 Tables and S1–S6 Figures. All in-text citations have been checked and corrected to match.

Response to Reviewer 2 (Statistical Concerns)

2a. Add 95% confidence intervals for AUROC values

Comment: Report 95% CIs for LightGBM and logistic regression AUCs in Table 4 and Results.

Response: We agree completely. Table 4 has been revised to include **bootstrap 95% confidence intervals** for both AUC-ROC and AUC-PR for all models. These were computed using 2,000 resamples (percentile method) on the out-of-fold prediction vectors. In the Results section (lines 520–535), we now state:

LightGBM achieved a mean AUC-ROC of 0.8622 (95% CI: 0.8558–0.8682) compared to logistic regression (0.8311, 95% CI: 0.8237–0.8384). The non-overlapping confidence intervals provide strong evidence that LightGBM’s advantage is not attributable to sampling variability.

2b. Perform a formal DeLong test

Comment: Run a DeLong test comparing the two correlated ROC curves and report p-value.

Response: We have now performed a **DeLong test** on the paired out-of-fold predictions (n = 13,105). The results are reported in a new **Table 5** (line 580) and in the text (lines 575–595):

LightGBM achieved a significantly higher AUC-ROC than logistic regression (0.8619 vs. 0.8310; AUC difference = +0.0309, 95% CI: [0.0264, 0.0355]; DeLong z = 13.298, $p < 0.0001$, two-sided). The 95% CI for the difference excludes zero entirely, confirming statistical significance.

Methods section has been updated to describe the DeLong test procedure (lines 410–420).

2c. Discuss practical/clinical significance of the AUROC difference

Comment: Address whether the 0.031 difference is clinically meaningful, not just statistically significant.

Response: We have added a new subsection to the Discussion titled **“Practical and Clinical Significance of the AUC-ROC Difference”** (lines 1050–1090). We explicitly argue:

In a low-harm administrative screening application — where the model triggers a clinical review rather than an autonomous discharge order — a 3.1 percentage-point improvement in AUC-ROC translates directly into a meaningfully larger proportion of patients being correctly ranked by discharge risk. This enables reviewers to prioritize attention more efficiently. Furthermore, LightGBM’s advantage extends consistently across AUC-PR, F1-score, and Brier score, providing a stronger justification than any single metric alone.

We also compare our findings to published benchmarks (Zeleke et al., Jain et al., Zeng et al.) to contextualize the magnitude.

Response to Reviewer 2 (Clinical Validity Concerns)

3a. Reframe language throughout the paper

Comment: Soften language implying direct clinical recommendations. Avoid “eligible for earlier discharge.”

Response: We have systematically revised the manuscript to replace over-claiming phrases. Examples:

Summary Table for Quick Reference

	Location	Original phrase	Replace with
1	Abstract Results	"eligible for earlier discharge"	"identified as potentially having reducible length of stay"
2	Abstract Results	"projecting 6,459 freed bed-days"	"projecting 6,459 potentially recoverable bed-days pending clinical review"
3	Abstract Conclusion	"identify discharge candidates"	"flag patients for clinical discharge review"
4	Table 7 note	"Eligible for early discharge"	"Flagged for discharge review"
5	Table 7 narrative	"classified as eligible for early discharge"	"flagged for clinical discharge review, identified as potentially having reducible length of stay"

6	Table 7 narrative	"stays could be shortened without compromising safety"	"stays may warrant clinical review for potential earlier discharge, subject to physician assessment"
7	Figure 2 note	"eligible for early discharge"	"flagged for discharge review"
8	Table 5 note	"eligible for earlier discharge"	"flagged for clinical review" / "potentially having reducible length of stay"
9	Table 5 note	"unnecessarily recommended for earlier discharge review"	"flagged for discharge review without a clinically reducible stay"
10	Table 5 narrative	"who would benefit from early discharge"	"whose length of stay may be reducible"
11	Table 5 narrative	end of false-positive sentence	add: "given that the model output triggers review rather than discharge"
12	Tables 8,11,12,13,14,16 notes	"Eligible for early discharge"	"Flagged for discharge review" (all instances)
13	Table 16 interpretation	"enables treatment of 1,328 additional patients"	"could theoretically accommodate 1,328 additional admissions if discharge review results in earlier discharge following clinical confirmation"
15	Discussion opening	"eligible for earlier discharge"	"flagged as potentially having reducible length of stay"
17	Calibration discussion	"any discharge recommendation is issued"	"any patient is flagged for discharge review"
18	Conclusion	"guide smarter…inpatient care decisions"	"support smarter…clinical discharge review processes"

These changes appear throughout the Abstract, Results, and Discussion (see lines 45, 310, 680, 1150).

3b. Strengthen the Limitations section

Comment: Explicitly acknowledge no chart review, no prospective trial, no external validation.

Response: The Limitations section (lines 1180–1220) has been substantially expanded to include:

"No clinical chart review was performed to verify whether patients would have been medically stable at the model-predicted discharge time. As a result, the optimization framework should be

interpreted as an administrative decision-support model rather than a clinically validated discharge protocol."
"No prospective interventional or randomized implementation trial was conducted. All projected reductions represent counterfactual retrospective estimates and should not be interpreted as proven causal effects."
"The study lacks external validation across independent hospital systems, insurance organizations, or later time periods. Transportability beyond the analyzed IHIO cohort remains uncertain."
"Real-world implementation would require governance structures including clinically validated discharge criteria, physician oversight, escalation pathways, audit mechanisms, and institutional accountability frameworks."

3c. Expand Future Research on clinical validation

Comment: Explicitly mention prospective RCT, clinical record validation, external validation.

Response: The Future Research section (lines 1225–1260) now includes three dedicated paragraphs on:

1. **Prospective clinical validation** – randomized controlled trial or pilot implementation study comparing model-guided vs. standard discharge.
2. **Clinical chart review** – structured adjudication of a patient subset to verify clinical stability at predicted discharge time.
3. **External validation** – testing across Social Security Organization hospitals, private facilities, armed forces hospitals, and future time periods.

Response to Reviewer 1

4a. Generalization beyond Iran IHIO

Comment: Discuss generalizability to other Iranian payers and other LMIC settings.

Response: We have added a new subsection to the Discussion: **"Generalizability to Other Payers and Settings"** (lines 1110–1145). This addresses:

- **Other Iranian payers:** Social Security Organization, Armed Forces Medical Services Fund — note that parallel retraining and recalibration would be needed due to different contractual LOS norms.
- **Other LMIC settings:** South Asia, sub-Saharan Africa, Latin America — adaptation requires local retraining on claims data, recalibration of LOS thresholds, and validation of payer-classification predictors.
- **Key message:** The core analytical pipeline requires no new data collection infrastructure and is transferable wherever administrative claims exist.

4b. Deeper interpretation of top predictors and policy implications

Comment: For the preferential currency flag (top predictor), explicitly discuss what intervention this implies.

Response: We have expanded the **Policy Implications** section (lines 1095–1110) to state:

"The payer-classification signal exists because hospitals apply systematically different discharge protocols to patients under different insurance categories, not because those patients have meaningfully different clinical trajectories. This directly implies that harmonization of discharge criteria and review procedures across citizen and non-citizen insurance pathways would attenuate the structural driver of avoidable prolonged stay — without requiring any technological intervention at all. The model is therefore not only a prediction tool but a diagnostic instrument that localizes the policy lever."

We also added parallel interpretations for age group and department type predictors.

Response to Formatting Corrections

5a. Table and figure captions

Comment: Ensure each has a number, a title, and explanatory notes below (not above), consistent with PLOS ONE style.

Response: We have reviewed and reformatted all **18 tables** and **9 main figures** plus **6 supplementary figures**. Each now follows the structure:

Table X. [Title sentence case]
[Table body]
Note. [Explanatory notes, abbreviations, CI definitions, bootstrap details]

All in-text citations have been verified.

5b. References

Comment: Fix reference 28 (stray period) and check overall formatting.

Response: Reference 28 has been corrected. The entire reference list has been re-checked against PLOS ONE style (journal name abbreviations, DOI inclusion where available, consistent punctuation). No other errors were found.

5c. PLOS ONE style template

Comment: Verify abstract word limits, section order, heading styles.

Response: We have checked the manuscript against the PLOS ONE template:

- Abstract: **298 words** (within 300-word limit)
- Section order: Abstract → Introduction → Methods → Results → Discussion → Conclusion → References → Supporting Information (correct)
- Headings: Hierarchical formatting verified (no orphan headings, consistent leveling)

We believe the revised manuscript now addresses all mandatory journal requirements, the statistical concerns raised by Reviewer 2 (including the DeLong test and confidence intervals), the clinical validity concerns (through reframed language and expanded limitations), and the formatting issues. We are grateful for the reviewers' careful reading and believe the paper is substantially stronger as a result.

We look forward to your decision.

Yours sincerely,
Shekoofeh Sadat Momahhed, PhD
Corresponding author
