# Supplementary material for: Machine learning–optimized discharge timing in typhoid care: Implications for clinical outcomes, cost efficiency, and health system performance
Source: PLoS One. 2026 Jul 23;21(7):e0354148. doi: 10.1371/journal.pone.0354148 (PMC13395383; doi:10.1371/journal.pone.0354148)
Supplement: Peer review history

# Peer review: Decision Letter 0

**DOI:** 10.1371/journal.pone.0354148.r001

**Authors:** Hilary Izuchukwu Okagbue

12 May 2026

PONE-D-26-09410

Machine Learning–Optimized Discharge Timing in Typhoid Care: Implications for Clinical Outcomes, Cost Efficiency, and Health System Performance

PLOS One

Dear Dr. Momahhed,

Thank you for submitting your manuscript to PLOS ONE. After careful consideration, we feel that it has merit but does not fully meet PLOS ONE’s publication criteria as it currently stands. Therefore, we invite you to submit a revised version of the manuscript that addresses the points raised during the review process.

==============================

**ACADEMIC EDITOR: Major revision is recommended.**

==============================

Please submit your revised manuscript by Jun 26 2026 11:59PM. If you will need more time than this to complete your revisions, please reply to this message or contact the journal office at plosone@plos.org. When you're ready to submit your revision, log on to https://www.editorialmanager.com/pone/ and select the 'Submissions Needing Revision' folder to locate your manuscript file.

Please include the following items when submitting your revised manuscript:

- A letter that responds to each point raised by the academic editor and reviewer(s). You should upload this letter as a separate file labeled 'Response to Reviewers'.
- A marked-up copy of your manuscript that highlights changes made to the original version. You should upload this as a separate file labeled 'Revised Manuscript with Track Changes'.
- An unmarked version of your revised paper without tracked changes. You should upload this as a separate file labeled 'Manuscript'.

If you would like to make changes to your financial disclosure, please include your updated statement in your cover letter. Guidelines for resubmitting your figure files are available below the reviewer comments at the end of this letter.

If applicable, we recommend that you deposit your laboratory protocols in protocols.io to enhance the reproducibility of your results. Protocols.io assigns your protocol its own identifier (DOI) so that it can be cited independently in the future. For instructions see: https://journals.plos.org/plosone/s/submission-guidelines#loc-laboratory-protocols. Additionally, PLOS ONE offers an option for publishing peer-reviewed Lab Protocol articles, which describe protocols hosted on protocols.io. Read more information on sharing protocols at https://plos.org/protocols?utm_medium=editorial-email&utm_source=authorletters&utm_campaign=protocols.

As the corresponding author, your ORCID iD is verified in the submission system and will appear in the published article. PLOS supports the use of ORCID, and we encourage all coauthors to register for an ORCID iD and use it as well. Please encourage your coauthors to verify their ORCID iD within the submission system before final acceptance, as unverified ORCID iDs will not appear in the published article. *Only*  the individual author can complete the verification step; PLOS staff *cannot*  verify ORCID iDs on behalf of authors.

We look forward to receiving your revised manuscript.

Kind regards,

Hilary Izuchukwu Okagbue, Ph.D

Academic Editor

PLOS One

Journal Requirements:

When submitting your revision, we need you to address these additional requirements.

1. Please ensure that your manuscript meets PLOS ONE's style requirements, including those for file naming. The PLOS ONE style templates can be found at

https://journals.plos.org/plosone/s/file?id=wjVg/PLOSOne_formatting_sample_main_body.pdf and https://journals.plos.org/plosone/s/file?id=ba62/PLOSOne_formatting_sample_title_authors_affiliations.pdf

2. Your ethics statement should only appear in the Methods section of your manuscript. If your ethics statement is written in any section besides the Methods, please move it to the Methods section and delete it from any other section. Please ensure that your ethics statement is included in your manuscript, as the ethics statement entered into the online submission form will not be published alongside your manuscript.

3. We note that you have indicated that there are restrictions to data sharing for this study. For studies involving human research participant data or other sensitive data, we encourage authors to share de-identified or anonymized data. However, when data cannot be publicly shared for ethical reasons, we allow authors to make their data sets available upon request. For information on unacceptable data access restrictions, please see http://journals.plos.org/plosone/s/data-availability#loc-unacceptable-data-access-restrictions.

Before we proceed with your manuscript, please address the following prompts:

a) If there are ethical or legal restrictions on sharing a de-identified data set, please explain them in detail (e.g., data contain potentially identifying or sensitive patient information, data are owned by a third-party organization, etc.) and who has imposed them (e.g., a Research Ethics Committee or Institutional Review Board, etc.). Please also provide contact information for a data access committee, ethics committee, or other institutional body to which data requests may be sent.

b) If there are no restrictions, please upload the minimal anonymized data set necessary to replicate your study findings to a stable, public repository and provide us with the relevant URLs, DOIs, or accession numbers. Please see http://www.bmj.com/content/340/bmj.c181.long for guidelines on how to de-identify and prepare clinical data for publication. For a list of recommended repositories, please see https://journals.plos.org/plosone/s/recommended-repositories. You also have the option of uploading the data as Supporting Information files, but we would recommend depositing data directly to a data repository if possible.

Please update your Data Availability statement in the submission form accordingly.

4. Please include captions for your Supporting Information files at the end of your manuscript, and update any in-text citations to match accordingly. Please see our Supporting Information guidelines for more information: http://journals.plos.org/plosone/s/supporting-information.

5. If the reviewer comments include a recommendation to cite specific previously published works, please review and evaluate these publications to determine whether they are relevant and should be cited. There is no requirement to cite these works unless the editor has indicated otherwise.

[Note: HTML markup is below. Please do not edit.]

Reviewers' comments:

Reviewer's Responses to Questions

**Comments to the Author**

1. Is the manuscript technically sound, and do the data support the conclusions?

The manuscript must describe a technically sound piece of scientific research with data that supports the conclusions. Experiments must have been conducted rigorously, with appropriate controls, replication, and sample sizes. The conclusions must be drawn appropriately based on the data presented.

Reviewer #1: Yes

Reviewer #2: Yes

**********

2. Has the statistical analysis been performed appropriately and rigorously?

Reviewer #1: Yes

Reviewer #2: No

**********

3. Have the authors made all data underlying the findings in their manuscript fully available?

The PLOS Data policy requires authors to make all data underlying the findings described in their manuscript fully available without restriction, with rare exception (please refer to the Data Availability Statement in the manuscript PDF file). The data should be provided as part of the manuscript or its supporting information, or deposited to a public repository. For example, in addition to summary statistics, the data points behind means, medians and variance measures should be available. If there are restrictions on publicly sharing data—e.g. participant privacy or use of data from a third party—those must be specified.

Reviewer #1: Yes

Reviewer #2: No

**********

4. Is the manuscript presented in an intelligible fashion and written in standard English?

PLOS ONE does not copyedit accepted manuscripts, so the language in submitted articles must be clear, correct, and unambiguous. Any typographical or grammatical errors should be corrected at revision, so please note any specific errors here.

Reviewer #1: Yes

Reviewer #2: Yes

**********

5. Review Comments to the Author

Please use the space provided to explain your answers to the questions above. You may also include additional comments for the author, including concerns about dual publication, research ethics, or publication ethics. (Please upload your review as an attachment if it exceeds 20,000 characters)

Reviewer #1: The manuscript addresses an important and relevant topic, and the use of machine learning for discharge optimization is promising. However, some areas require further clarification.

1. The authors should further discuss the model’s generalization beyond the study setting and consider external validation.

2. The predictor warrants deeper interpretation, particularly regarding policy implications.

Reviewer #2: This is a thoughtful and ambitious study that provides valuable insight into system-level inefficiencies in typhoid patient management. The work highlights an important healthcare challenge and demonstrates the potential of machine learning approaches in improving operational decision-making. However, the study currently draws conclusions that go beyond what the evidence can confidently support. In particular, it tends to blur the distinction between predicting discharge patterns and recommending earlier patient discharge, which requires much stronger clinical validation and safety evaluation.

To strengthen the study and improve its clinical credibility, several important steps may be considered:

-Validate the model findings against detailed medical records for a subset of patients to confirm the accuracy of typhoid diagnoses and ensure that patients would have been clinically stable at the predicted discharge times.

-Conduct a prospective pilot study or randomized controlled trial comparing model-guided discharge practices with standard clinical care. Key safety outcomes such as readmission, mortality, and clinical deterioration should be carefully monitored.

-Perform external validation using data from a different hospital system or from another time period to assess the generalizability and robustness of the model.

-Develop a clear implementation framework that includes clinical decision rules, monitoring mechanisms, escalation pathways, and accountability structures before considering real-world deployment.

-Involve healthcare administrators and policymakers to address the underlying systemic issue—particularly payer-driven prolonged hospital stays—rather than only predicting when such delays may occur.

Another important limitation of the study is the absence of statistical testing when comparing machine learning models. While the gradient-boosted classifier achieved an AUROC of 0.862 compared to 0.831 for logistic regression, the reported difference of 0.031 is relatively small and may or may not be statistically meaningful. Without formal statistical evaluation, claims that one model “outperforms” another remain insufficiently supported.

A few formatting correctios are appreciated including tables and figures captions, references etc.

Several key statistical elements are missing:

-Confidence intervals (95% CIs) for AUROC values should be reported so readers can assess the precision and uncertainty of the estimates. Overlapping confidence intervals may indicate that the observed performance difference is not statistically significant.

-A formal statistical comparison, such as the DeLong test for correlated ROC curves, should be performed to determine whether the AUROC difference is significant at an accepted threshold (e.g., p < 0.05).

-The study should also discuss the practical or clinical significance of the observed performance improvement. ------Although a 3.1% increase in AUROC may appear favorable statistically, its real-world importance depends heavily on the clinical context. In high-stakes settings such as discharge decision-making, even small performance differences can have meaningful implications for patient safety; however, in lower-risk administrative applications, the improvement may be negligible.

Overall, the study addresses an important problem and demonstrates promising analytical work, but additional clinical validation, statistical rigor, and implementation planning are necessary before the findings can support changes to patient discharge practices.

**********

6. PLOS authors have the option to publish the peer review history of their article (what does this mean?). If published, this will include your full peer review and any attached files.

If you choose “no”, your identity will remain anonymous but your review may still be made public.

**Do you want your identity to be public for this peer review?**  For information about this choice, including consent withdrawal, please see our Privacy Policy.

Reviewer #1: **Yes:** Dr Kingsley Attai

Reviewer #2: No

**********

[NOTE: If reviewer comments were submitted as an attachment file, they will be attached to this email and accessible via the submission site. Please log into your account, locate the manuscript record, and check for the action link "View Attachments". If this link does not appear, there are no attachment files.]

To ensure your figures meet our technical requirements, please review our figure guidelines: https://journals.plos.org/plosone/s/figures

You may also use PLOS’s free figure tool, NAAS, to help you prepare publication quality figures: https://journals.plos.org/plosone/s/figures#loc-tools-for-figure-preparation.

NAAS will assess whether your figures meet our technical requirements by comparing each figure against our figure specifications.

# Author comment: Author response to Decision Letter 1

**DOI:** 10.1371/journal.pone.0354148.r002

21 May 2026

Thank you for the opportunity to revise our manuscript. We have carefully addressed all mandatory journal requirements, the statistical concerns raised by Reviewer 2, the clinical validity and generalizability questions from Reviewer 1, and the formatting issues noted by the editor. First, regarding the journal’s mandatory requirements: we have moved the ethics statement into the Methods section (line 245), revised the Data Availability statement with full legal and ethical justification for restricted access while keeping our analysis code publicly available on GitHub, and added formal captions for all supplementary figures and tables at the end of the manuscript. Second, to address Reviewer 2’s statistical concerns, we have added 95% bootstrap confidence intervals for AUC-ROC and AUC-PR for all models in Table 4, performed a formal DeLong test comparing LightGBM and logistic regression (AUC difference = +0.0309, 95% CI: 0.0264–0.0355, DeLong z = 13.298, p < 0.0001), and expanded the Discussion to explicitly address the practical clinical significance of this difference in the context of a low-harm administrative screening tool. We have also systematically softened potentially over-claiming language throughout the manuscript—replacing phrases like “eligible for earlier discharge” with “flagged for clinical review” or “identified as potentially having reducible length of stay”—and substantially strengthened the Limitations section to acknowledge the absence of clinical chart review, prospective trials, and external validation, while expanding the Future Research section to explicitly call for RCTs, clinical record validation, and external validation across other Iranian payers and LMIC settings. Finally, we have addressed Reviewer 1’s concerns by adding a dedicated subsection on generalizability to other Iranian payers (Social Security Organization, Armed Forces Medical Services Fund) and other LMIC contexts, and provided deeper policy interpretation of the top predictors—particularly the preferential currency flag—arguing that the model identifies a remediable structural mismatch in discharge protocols across payer pathways rather than merely predicting risk. All tables and figures have been reformatted to PLOS ONE style, reference 28 has been corrected, and the manuscript structure now fully complies with journal requirements. We believe these revisions have substantially strengthened the scientific rigor, clinical transparency, and policy relevance of our work, and we are grateful for the reviewers’ constructive guidance.

Attachment Submitted filename: Response to Reviewers.pdf

# Peer review: Decision Letter 1

**DOI:** 10.1371/journal.pone.0354148.r003

**Authors:** Hilary Izuchukwu Okagbue

6 Jul 2026

<p>Machine Learning–Optimized Discharge Timing in Typhoid Care: Implications for Clinical Outcomes, Cost Efficiency, and Health System Performance

PONE-D-26-09410R1

Dear Dr. Momahhed,

We’re pleased to inform you that your manuscript has been judged scientifically suitable for publication and will be formally accepted for publication once it meets all outstanding technical requirements.

Within one week, you’ll receive an e-mail detailing the required amendments. When these have been addressed, you’ll receive a formal acceptance letter and your manuscript will be scheduled for publication.

An invoice will be generated when your article is formally accepted. Please note, if your institution has a publishing partnership with PLOS and your article meets the relevant criteria, all or part of your publication costs will be covered. Please make sure your user information is up-to-date by logging into Editorial Manager at Editorial Manager® and clicking the ‘Update My Information' link at the top of the page. For questions related to billing, please contact billing support.

If your institution or institutions have a press office, please notify them about your upcoming paper to help maximize its impact. If they’ll be preparing press materials, please inform our press team as soon as possible -- no later than 48 hours after receiving the formal acceptance. Your manuscript will remain under strict press embargo until 2 pm Eastern Time on the date of publication. For more information, please contact onepress@plos.org.

Kind regards,

Hilary Izuchukwu Okagbue, Ph.D

Academic Editor

PLOS One

Additional Editor Comments (optional):

Reviewers' comments:

Reviewer's Responses to Questions

**Comments to the Author**

1. If the authors have adequately addressed your comments raised in a previous round of review and you feel that this manuscript is now acceptable for publication, you may indicate that here to bypass the “Comments to the Author” section, enter your conflict of interest statement in the “Confidential to Editor” section, and submit your "Accept" recommendation.

Reviewer #1: All comments have been addressed

Reviewer #2: All comments have been addressed

**********

2. Is the manuscript technically sound, and do the data support the conclusions?

The manuscript must describe a technically sound piece of scientific research with data that supports the conclusions. Experiments must have been conducted rigorously, with appropriate controls, replication, and sample sizes. The conclusions must be drawn appropriately based on the data presented.

Reviewer #1: Yes

Reviewer #2: Yes

**********

3. Has the statistical analysis been performed appropriately and rigorously?

Reviewer #1: Yes

Reviewer #2: Yes

**********

4. Have the authors made all data underlying the findings in their manuscript fully available?

The PLOS Data policy requires authors to make all data underlying the findings described in their manuscript fully available without restriction, with rare exception (please refer to the Data Availability Statement in the manuscript PDF file). The data should be provided as part of the manuscript or its supporting information, or deposited to a public repository. For example, in addition to summary statistics, the data points behind means, medians and variance measures should be available. If there are restrictions on publicly sharing data—e.g. participant privacy or use of data from a third party—those must be specified.

Reviewer #1: Yes

Reviewer #2: Yes

**********

5. Is the manuscript presented in an intelligible fashion and written in standard English?

PLOS ONE does not copyedit accepted manuscripts, so the language in submitted articles must be clear, correct, and unambiguous. Any typographical or grammatical errors should be corrected at revision, so please note any specific errors here.

Reviewer #1: Yes

Reviewer #2: Yes

**********

6. Review Comments to the Author

Please use the space provided to explain your answers to the questions above. You may also include additional comments for the author, including concerns about dual publication, research ethics, or publication ethics. (Please upload your review as an attachment if it exceeds 20,000 characters)

Reviewer #1: I have carefully reviewed the revised manuscript and the authors’ responses to the reviewers' comments. The authors have addressed the concerns raised during the previous review round and have made appropriate revisions that have improved the clarity and quality of the manuscript. The study presents a valuable application of machine learning to optimize discharge timing in typhoid care, with important implications for clinical outcomes, healthcare resource utilization, and health system performance. I have no further substantive comments and believe the manuscript is suitable for publication in its current form.

Reviewer #2: The authors have adequately addressed the comments raised in a previous round of review. This manuscript is acceptable for publication provided that all the editorial requirements are fulfilled.

**********

7. PLOS authors have the option to publish the peer review history of their article (what does this mean?). If published, this will include your full peer review and any attached files.

If you choose “no”, your identity will remain anonymous but your review may still be made public.

**Do you want your identity to be public for this peer review?**  For information about this choice, including consent withdrawal, please see our Privacy Policy.

Reviewer #1: No

Reviewer #2: **Yes:** Madhuri Wakode

**********

# Editor report: Acceptance letter

**DOI:** 10.1371/journal.pone.0354148.r004

**Authors:** Hilary Izuchukwu Okagbue

PONE-D-26-09410R1

PLOS One

Dear Dr. Momahhed,

I'm pleased to inform you that your manuscript has been deemed suitable for publication in PLOS One. Congratulations! Your manuscript is now being handed over to our production team.

At this stage, our production department will prepare your paper for publication. This includes ensuring the following:

* All references, tables, and figures are properly cited

* All relevant supporting information is included in the manuscript submission,

* There are no issues that prevent the paper from being properly typeset

You will receive further instructions from the production team, including instructions on how to review your proof when it is ready. Please keep in mind that we are working through a large volume of accepted articles, so please give us a few days to review your paper and let you know the next and final steps.

Lastly, if your institution or institutions have a press office, please let them know about your upcoming paper now to help maximize its impact. If they'll be preparing press materials, please inform our press team within the next 48 hours. Your manuscript will remain under strict press embargo until 2 pm Eastern Time on the date of publication. For more information, please contact onepress@plos.org.

You will receive an invoice from PLOS for your publication fee after your manuscript has reached the completed accept phase. If you receive an email requesting payment before acceptance or for any other service, this may be a phishing scheme. Learn how to identify phishing emails and protect your accounts at https://explore.plos.org/phishing.

If we can help with anything else, please email us at customercare@plos.org.

Thank you for submitting your work to PLOS ONE and supporting open access.

Kind regards,

PLOS ONE Editorial Office Staff

on behalf of

Dr Hilary Izuchukwu Okagbue

Academic Editor

PLOS One
